# Evaluation of tropical–temperate transitions: An example of climatic characterization in the Asian Palmate group of Araliaceae

**DOI:** 10.1002/ajb2.16059

**Published:** 2022-09-23

**Authors:** Marina Coca‐de‐la‐Iglesia, Nagore G. Medina, Jun Wen, Virginia Valcárcel

**Affiliations:** ^1^ Departamento de Biología Universidad Autónoma de Madrid Madrid 28049 Spain; ^2^ Centro de Investigación en Biodiversidad y Cambio Global (CIBC‐UAM) Universidad Autónoma de Madrid Madrid 28049 Spain; ^3^ Department of Botany/MRC 166 Smithsonian Institution Washington, DC USA

**Keywords:** Araliaceae, Asian Palmate Group, data cleaning, ecoregions, GEnS classification, Holdridge classification, Köppen–Geiger classification, latitudinal zonation, online biodiversity databases, world climatic regionalization

## Abstract

**Premise:**

There has been a great increase in using climatic data in phylogenetic studies over the past decades. However, compiling the high‐quality spatial data needed to perform accurate climatic reconstructions is time‐consuming and can result in poor geographical coverage. Therefore, researchers often resort to qualitative approximations. Our aim was to evaluate the climatic characterization of the genera of the Asian Palmate Group (AsPG) of Araliaceae as an exemplar lineage of plants showing tropical–temperate transitions.

**Methods:**

We compiled a curated worldwide spatial database of the AsPG genera and created five raster layers representing bioclimatic regionalizations of the world. Then, we crossed the database with the layers to climatically characterize the AsPG genera.

**Results:**

We found large disagreement in the climatic characterization of genera among regionalizations and little support for the climatic nature of the tropical–temperate distribution of the AsPG. Both results are attributed to the complexity of delimiting tropical, subtropical, and temperate climates in the world and to the distribution of the study group in regions with transitional climatic conditions.

**Conclusions:**

The complexity in the climatic classification of this example of the tropical–temperate transitions calls for a general climatic revision of other tropical–temperate lineages. In fact, we argue that, to properly evaluate tropical–temperate transitions across the tree of life, we cannot ignore the complexity of distribution ranges.

The use of climatic data in phylogenetic studies has become routine since it often provides insightful information to understand better the evolutionary history of lineages (e.g., Pyron and Wiens, 2013; Edwards et al., 2017; Nürk et al., 2018; Albaladejo et al., 2021). To do so, both robust phylogenies and large amounts of good quality climatic data are needed (Budic and Dormann, 2015). However, to obtain accurate climatic data and conduct robust phylogenetic reconstructions, high‐quality geographical information of the study group is advocated as the starting input data (Hortal et al., 2007). Because of this, the compilation of high‐quality spatial databases is a cornerstone in these integrative approaches.

The huge amount of biodiversity data that has been collected by naturalists and scientists during the last centuries has been compiled in online databases in the last decades, and it is now generally available with just one click (GBIF: GBIF.org, 2021; TRY: Fraser, 2020; Specieslink: SpeciesLink, 2021). However, the process from the initial download‐click to the final compilation of high‐quality spatial databases is long and complex. Indeed, large volumes of data often require intense data cleaning (data parsing and homogenization) before they are ready for analyses. Dealing with typographical and other frequent errors, such as inaccurate georeferences and incorrect taxonomic names (due to faulty identifications or use of outdated nomenclature), represents a very time‐consuming aspect of data cleaning (Soberón and Peterson, 2004), even for databases such as GBIF, which contain categories to facilitate the cleaning of invalidly georeferenced records (Yesson et al., 2007). Data homogenization is another time‐consuming step in data cleaning and may also become a serious limitation when different online data sources are merged (Turnhout and Boonman‐Berson, 2011).

Once the data cleaning is done, a geographical coverage testing is needed to evaluate the representativeness and accuracy of the spatial database before performing any further analysis. This geographical coverage testing is done by comparing the spatial database to other sources of geographical information (e.g., monographs, checklists, and local or regional floras) and is an essential step since online databases reflect incomplete knowledge of species distributions that may be uneven across the world and the tree of life (Hortal et al., 2015; Meyer et al., 2016). For example, well‐developed regions, legally protected areas, and temperate deciduous woodlands have traditionally been better sampled (Martin et al., 2012). This sampling bias can be explained by different factors that may have nothing to do with biodiversity itself (e.g., economic wealth, use of a modern or an extinct language, geographical accessibility, and security of the region), but these factors can have a strong impact on data gathering (Amano and Sutherland, 2013). Therefore, to take full advantage of the enormous potential of online spatial databases, we need to assess and consider gaps in biodiversity knowledge when making conclusions (Hortal et al., 2008; Boakes et al., 2010).

As a result, even after extensive data cleaning, the spatial database may poorly represent the distribution of the study group, and so it should not be used to describe its climatic preferences. In this context, qualitative approaches offer a synthetic and easy‐to‐apply approach to test evolutionary climatic hypotheses (e.g., Triplett et al., 2014; Spriggs et al., 2015; Jezovit et al., 2017; Campbell et al., 2019). Indeed, a very frequent strategy is to classify taxa according to the categories of a given climatic classification system (e.g., Köppen and Geiger, 1936) and to use this categorization as the input data for the phylogenetic reconstructions (e.g., Antonelli et al., 2015; Gagnon et al., 2019; Silva et al., 2021). Besides, the use of categories facilitates comparisons and allows questions to be posed as testable hypotheses (e.g., that tropical lineages are more diverse than temperate ones; e.g., Erwin, 2009).

The use of categorical classifications in the study of lineage evolution has a long history of successful applications. For example, a seminal study in the 1990s discovered that tropical and temperate family pairs were common in flowering plants using the categorical distinction between tropical and temperate regions (Araliaceae‐Apiaceae, Apocinaceae‐Asclepediaceae, Brassicaceae‐Capparaceae, Moraceae‐Uricaceae, Sapindaceae‐Hippocastanaceae; Judd et al., 1994). Later, advances in phylogenetics unveiled a rich variety of phylogenetic and diversification patterns between tropical and temperate lineages in plants (e.g., Bambusseae, Kelchner and Clark, 1997; Magnoliaceae, Azuma et al., 2001; Liu et al., 2015; Vitaceae, Nie et al., 2010; Pooideae, Schubert et al., 2019; *Prunus* of Rosaceae, Zhao et al., 2018) and animals (e.g., ants, Moreau and Bell, 2013; salamanders, Wiens et al., 2007; fishes, Winger et al., 2014; birds, Price et al., 2009). These findings have stimulated fruitful discussions surrounding the differential ability of lineages to overcome the tropical–temperate transition and their in situ evolutionary success and the importance of niche conservatism for the latitudinal diversity gradient (e.g., Ricklefs, 2004; Wiens and Donoghue, 2004; Jablonski et al., 2006; Mittelbach et al., 2007; Donoghue, 2008; Jansson et al., 2013). For most of the early studies, the distinction between tropical and temperate was purely latitudinal (e.g., Judd et al., 1994). Therefore, the early documentation of this tropical–temperate pattern was probably not intended to reflect climate differences. However, the notion that climate drives latitudinal diversity patterns has a long history (Humboldt, 1808) and has deeply influenced the interpretation of the causes that drive the transitions between tropical and temperate climates. Therefore, it is not surprising that as the number of cases increased across the whole tree of life, this broad‐scale geographical pattern of tropical–temperate lineages was interpreted in terms of niche shifts of the taxa involved (e.g., Wiens and Donoghue, 2004; Mittelbach et al., 2007; Donoghue, 2008; Jansson et al., 2013). The fact that multiple delineations of the tropical and the temperate regions have been based on climatic variables (e.g., temperature in relation to freezing point, Wright et al., 2009; relation between seasonality and daily temperature, Laing and Evans, 2015) has no doubt contributed to these kinds of interpretations. The use of quantitative approaches to characterize climatic preferences and analyze the transitions between tropical and temperate regions (e.g., Pyron and Wiens, 2013; Kerkhoff et al., 2014; Edwards et al., 2017) has, to some extent, overcome the problems derived from using categorization to characterize taxa. Yet, categorical classifications remain essential because they offer an integrative perspective of climate that allows the main trends throughout the world to be effectively summarized, rather than quantitative approaches using just the climatic preferences of taxa. Further, classifications provide information about the context in which the species distributions are embedded. Two taxa with distributions restricted to a similar climate can be surrounded by very different climatic conditions. Finally, classifications allow comparisons with early ground‐breaking literature in which the study of the tropical–temperate transition was only possible through qualitative characterizations. Indeed, many studies have continued to resort to simplifications such as temperate and tropical categories to interpret and discuss results based on quantitative climatic data (e.g., Jansson et al., 2013; Antonelli et al., 2015; Egan et al., 2018; Ávila‐Lovera and Garcillán, 2021). Quantitative studies refer to such simplifications because, the study of tropical–temperate transitions began with hypotheses that used categories, and, thus, comparisons with early studies require translating the quantitative results to a qualitative context. In summary, we still use early systematic works and their categorical perspectives to formulate evolutionary hypotheses (e.g., Liede‐Schumann et al., 2012; Preston and Sandve, 2013; Valcárcel et al., 2014), interpret our results, and make comparisons among studies that used different approaches. However, early works on the study of tropical–temperate lineages rarely stated the criteria used to identify taxa within one or the other category; thus, determining whether the categories were intended to reflect geography, biome regionalizations, or pure climatic classes is not straightforward. Nonetheless, these categorical studies frequently used climatic differences or similarities between the categories to explain the results (e.g., Azuma et al., 2001; Liede‐Schumann et al., 2012; Preston and Sandve, 2013), which implies that although the categorization was purely geographical or biogeographical, the authors considered that the categories reflected differences in climate. This situation has been common in evolutionary studies, which limits the robustness of the assessments and the comparisons to other studies (Drescher et al., 2013).

A good example of tropical–temperate plant lineages is the Asian Palmate group of the Araliaceae (hereafter AsPG), which represents the largest named clade within the family Araliaceae. The distribution of the AsPG extends through vast regions in East Asia, Southeast Asia, Europe, North Africa, the Americas, and North Oceania. Across this broad range, most genera are concentrated in tropical and subtropical latitudes (70% of the genera [16], 94% of the species [ca 900]), in contrast with the scarce representation of the group in temperate latitudes (30% of genera [7], 6% of the species [ca 55]; Plunkett et al., 1996; Wen et al., 2001). Consequently, the AsPG had been considered to be mostly tropical (Plunkett et al., 1996, Wen et al., 2001). Indeed, the numerous biogeographic studies of this group place the origin of the AsPG in different areas within the tropics (Mitchell et al., 2012; Li and Wen, 2014; Valcárcel and Wen, 2019; Plunkett et al., 2020). The inferred tropical origin, together with the fact that the temperate genera evolved independently, each sister to a different tropical or subtropical lineage (Valcárcel and Wen, 2019), implies that the transition between tropical and temperate regions has occurred multiple times in the AsPG. Moreover, it seems that these transitions occurred early in the evolution of the group and during a short time because the divergence of these temperate–tropical sister lineages took place during the ancient radiation of the AsPG in the Eocene (Valcárcel and Wen, 2019). The emergence of the tropical and temperate lineages during this early radiation has been attributed to recurrent latitudinal range shifts. These latitudinal migrations along with episodes of lineages isolation and connection were linked to the alternation of the warm and cool periods during the Eocene (Valcárcel and Wen, 2019). Based on these previous studies, we hypothesized that the divergence of the tropical–temperate lineages in the AsPG was mainly driven by climatic forces. Given that soon after the origin of the AspG in the tropics, all the main tropical and temperate generic lineages diverged during an explosion of diversification, we also hypothesized that the acquisition of the temperate climatic niche may have acted as an early evolutionary driver at the origin of the AsPG (Valcárcel et al., 2014; Valcárcel and Wen, 2019). These hypotheses can only be envisioned if the tropical–temperate distribution of the AsPG genera correlates with contrasting tropical–temperate climatic preferences. However, there has not been any explicit climatic characterization of the AsPG genera, and thus, neither the tropical and temperate climatic nature of its genera, nor the tropicality of the whole clade have been critically evaluated.

Our main objective was to characterize the climatic preferences of the 23 AsPG genera to evaluate the climatic nature of the tropical–temperate distribution in the largest named clade of Araliaceae. To do so, we designed a semiquantitative approach that uses online biodiversity repositories to compile georeferenced databases and extract qualitative climatic data. To extract qualitative climatic data, we used five layers that represent the climatic regionalizations of the world according to five bioclimatic classifications that are frequently used in evolutionary studies. The spatial layers representing the five climatic regionalizations of the world selected were also compared to evaluate background patterns in world bioclimatic regionalizations. The specific objectives were to (1) analyze the efficiency of the qualitative regionalizations for capturing transitional climates across the world, (2) compile a worldwide high‐quality point‐occurrence database that provides an accurate representativeness of the geographical distribution of the AsPG genera, (3) compile a high‐quality climatic database of the AsPG genera, (4) characterize the geographical range and climatic preferences of the AsPG genera, and (5) evaluate the impact of using different climatic classifications on the climatic characterization of the AsPG.

## MATERIALS AND METHODS

### Spatial characterization of the AsPG

#### Spatial data collection

Eight online databases were used to download the spatial records of the AsPG from March 2018 to April 2020 (Table 1). Downloads were done either through the websites associated with these databases (Neotropical Plant Portal: https://serv.biokic.asu.edu/neotrop/plantae/, Neotropical Plant Portal, 2021; NBN: nbnatlas.org/, NBN Atlas, 2021; Tropicos: www.tropicos.org/, Tropicos.org, 2021; TRY: www.try-db.org/, Fraser, 2020; WPKorea: florakorea.myspecies.info/, Chang and Kim, 2015), or using the available R packages (rgibf from GBIF, Chamberlain et al., 2020a; DOI references for the original downloads are available in Zenodo from Coca‐de‐la‐Iglesia et al., 2021a, BIEN from BIEN (Maitner et al., 2018), and spocc from iNaturalist (Chamberlain et al., 2020b). Since our aim was to provide a geographical and climatic characterization at the genus level, searches for downloads were done by genus instead of species, except for six genera (*Cephalopanax* G.M. Plunkett, Lowry & D.A.Neill; *Crepinella* Marchal; *Didymopanax* Decne. & Planch.; *Frodinia* Lowry & G.M. Plunkett; *Heptapleurum* Gaertn.; and *Sciadophyllum* P. Browne). These six newly described or reinstated genera are the result of a major taxonomic rearrangement of the genus *Schefflera* J.R. Forst. & G. Forst. (Fiaschi et al., 2020; Lowry and Plunkett, 2020; Lowry et al., 2019, 2020; Plunkett et al., 2021; see also Plunkett et al., 2020). Because of the polyphyly of *Schefflera* s.l. (i.e., as it had previously been circumscribed), the searches for these six genera were completed using species names rather than by genus. Also, because these taxonomic rearrangements were being published during our downloading process, the nomenclature found on online databases had not yet been updated, and therefore searches for these species were completed using the respective synonyms for *Schefflera* species. To identify which of the former *Schefflera* species belong to the AsPG, we matched the species distribution of all species of *Schefflera* s.l. (as recorded in the World Checklist of Araliaceae; Frodin and Govaerts, 2003), with the geographical ranges of the main linages of the former circumscription of *Schefflera* s.l. as described in Plunkett et al. (2005) and later checked with the synonyms included in the recent taxonomic rearrangements (Fiaschi et al., 2020; Lowry and Plunkett, 2020; Lowry et al., 2019, 2020; Plunkett et al., 2020, 2021) and the list of species available from the Araliaceae Central website (http://legacy.tropicos.org/Project/Araliaceae; Lowry and Plunkett, 2022).

**Table 1:** Summary of the compilation and cleaning information of the spatial database of the Asian Palmate Group of Araliaceae (AsPG). Name of herbaria are provided by their abbreviation in the Index Herbarioum (Thiers, 2016) except for virtual herbaria.

Original source	No. records		% of records
	Before cleaning	After cleaning	
**Online databases**	**679,857**	**471,811**	**99.89**
GBIF^a^	633,315	448,846	94.17
BIEN	30,558	16,793	3.52
NBN	8871	4870	1.02
iNaturalist	6212	3739	0.78
Neotropical Plant Portal	1560	1119	0.23
Tropicos	690	643	0.13
WPKorea	143	132	0.03
TRY	68	52	0.01
**Herbaria** b	**426**	**379**	**0.08**
AVH^c^	11	7	–
CVH^d^	134	111	–
E	79	68	–
US	121	120	–
MO	58	52	–
NY	22	20	–
K	1	1	–
**Literature** b	**145**	**131**	**0.03**

^a^ DOI references for the original downloads are available in Zenodo (Coca‐de‐la‐Iglesia et al., 2021a).

^b^ Records not uploaded in any of the online databases analyzed.

^c^ Australasian Virtual Herbarium, website https://avh.chah.org.au/.

^d^ Chinese Virtual Herbarium, website https://www.cvh.ac.cn/index.php.

To increase the number of records for poorly represented genera at a global or regional scale in the online databases used, we did a targeted search of georeferenced specimens in eight herbaria (Table 1). Gaps in distributional knowledge persisted for certain genera and/or regions and were fulfilled by targeted searches of localities in systematic studies (*Trevesia* Vis., *Hedera* L., *Macropanax* Miq., *Sciodaphyllum*, *Gamblea* C.B. Clarke, and *Heteropanax* Seem.; Jebb, 1998; Shang et al., 2000; Jamir and Pandey, 2003; Heriyanto and Sawitri, 2007; Prabhu et al., 2010; Tagane et al., 2017; Jiménez‐Montoya and Idárraga‐Piedrahíta, 2018; Ong, 2018; Amini et al., 2020). Because most of these localities lacked longitude and latitude coordinates, coordinates were determined using GeoLocate Web Application (Rios and Bart, 2010). A position point was marked in zones with the highest probability of appearance of the species, according to the description of the locality provided in each record. Google Earth and Google Street View were also used to improve precision.

Finally, we extracted the elevation of each record because multiple records lacked elevation or provide a range in elevational information. To do so, we grouped the 60 elevational files available in WorldClim (30 secs; Fick and Hijmans, 2017) to form a new layer named ElevationWorldClim30sec that can be obtained from Zenodo (Coca‐de‐la‐Iglesia et al., 2021b).

#### Data cleaning

To clean the spatial data that was compiled, we developed a script in R (R Core Team, 2018) that automates the process to decrease the processing time (Coca‐de‐la‐Iglesia et al., 2022). This cleaning step was designed to achieve two main objectives: (1) to homogenize data and remove duplicates and (2) to reduce the effect of spatial uncertainty and non‐natural records on the climatic characterization of genera.

For the first objective, we homogenized the administrative information across all records according to two types of standardized country codes and using vector layers in QGIS 3.4.3‐Madeira (QGIS Development Team, 2021). We used the coordinates of records to extract the countries for the third level of the geographical codes of the Taxonomic Databases Working Group (TDWG; Brummitt, 2001) from the layer available in the GitHub repository (Desmet and Page, 2007) and the 2‐letter ISO‐3166‐1 code for each record as obtained from Admin‐0 Countries layer of Natural Earth 4.1.0 (Patterson and Kelso, 2020). This procedure also allowed us to correct country typographical errors in the original data. We then proceeded to remove the replicate records (i.e., records represented in two or more of the online data sources). Online data sources had different degree of incompleteness regarding voucher information (collector name and number). Because of this, we selected a combination of eight fields from the database to identify replicate records (species name: “Spp”; collection year: “Year”; country code: “CountryCode”; locality: “Locality”; longitude: “Longitude”; latitude: “Latitude”; elevation; “Elevation”; Collector number or herbarium number: “catalogNumber”; and type of record: “basisOfRecord”). Once identified, only one of the replicate records was kept, and the remaining were removed.

For the second objective of the data cleaning, we first removed all records that contained spatial uncertainty, including records with none or less than two decimals in their coordinates and those with erroneous coordinates. We defined a threshold of 10 km from the coastal line to distinguish between erroneous records (>10 km) from misplaced records (≤10 km). Because of the variability in the land surface limits of the different layers, we used one of the 19 Worldclim bioclimatic layers (bio1: Annual Mean Temperature; Fick and Hijmans, 2017; https://www.worldclim.org/) to establish the limits of land surface. Using this layer as a template, we removed all erroneous records (i.e., those at >10 km from coastal line). We decided to keep misplaced records (i.e., those ≤10 km from coastal line; hereafter “coastal line records”) to avoid the loss of seaboard environments that might be potentially informative for the climatic characterization of genera. To keep these coastal line records, their original coordinates were recalculated to meet the nearest climatic cell of the template.

Finally, to avoid the noise resulting from the inclusion of cultivars and records outside the natural ranges of genera we identified and removed all non‐natural wild records. We identified cultivated records as those in which the locality description included any of the following words: cultivated, cultivado, park, parque, garden, jardín, castel, castillo, golf, cementerio, zoo, farm. Each record identified as cultivated was manually checked before removal to ensure that native stands (e.g., from National Parks) were not erroneously deleted. To remove non‐native records, we first created a vector to represent the natural range of each genus using the botanical countries of the TDWG geographical standard (Brummitt, 2001) as the spatial unit. To do so, we used the third level of TDWG code of the botanical countries of the genus native range as in the World Checklist of Selected Plant Families (WCSP; Govaerts et al., 2008). These vectors were cross‐checked with the country‐code field that included the third level of TDWG code in the database (“CountryCodeTDWG”) to remove records from non‐matching countries (i.e., records outside the native range).

#### Spatial data analyses

To represent the native distribution of the AsPG as a whole, a point‐occurrence map was created using all records from the cleaned database. To evaluate the spatial biodiversity trends at a global scale, we built a bubble map by using the botanical countries of the clade as the spatial units and the number of genera per spatial unit as the value to estimate the size of bubbles. Then, we built heat maps as a proxy to evaluate sampling effort in the AsPG across the world. Because global patterns in the sampling effort may impact other patterns at a finer scale, we decided to build three heat maps: Asia (including Oceania), Europe (including North Africa), and the Americas. To build each heat map, we used a cell of one geographical degree as the spatial unit and the number of records per spatial unit to estimate the sampling effort in each cell. To categorize cells according to the number of records within a gradient, we used Jenks natural breaks. To identify sampling hotspots in each region (areas including cells with high levels of sampling effort), we first estimated the minimum and maximum sampling effort in each cell category for each heat map. To do so, we compared the minimum and maximum number of records of each cell category in each region with the maximum number of records per cell detected in that given region. As a result, we calculated two sampling effort values for each cell category in each region that represent the range of sampling effort. Cell categories including 25% of sampling effort within their ranges were considered as sampling hotspots. Finally, to evaluate temporal sampling patterns across the world in the AsPG, we plotted the accumulated number of occurrences over time in each region using the ggplot2 package (Wickham et al., 2021) in R version 3.5 (R Core Team, 2018) and the same three regions as for the heat maps. Due to the limited volume of data for years before 1900 (1479 records), occurrences recorded before that date were not used for the temporal series.

To represent the native ranges of the AsPG genera, 23 point maps were made (one per genus). To evaluate the sampling effort per genus, 23 world heat maps were built using the procedure described above for the entire AsPG clade. Finally, to identify range‐restricted vs. widespread genera, we estimated the area of occupancy (AOO) and the extent of occurrence (EOO) in 4‐km^2^ cells for each genus using a distance of 2 km as the buffer to estimate the AOO, as recommended by International Union for Conservation of Nature (IUCN, 2012). These estimates were completed using GeoCAT (Bachman et al., 2011) for all genera except for *Dendropanax* Decne. & Planch. (because of its large disjunction between Asia and the Neotropics) and *Hedera* (because of its numerous occurrences). Instead, AOO and EOO for these two genera were estimated using QGIS, calculating the total area of convex hull created around all occurrences as the estimate for the EOO. Finally, in characterizing genera as restricted, we applied the minimum AOO threshold of the B2 criterion of the IUCN for the vulnerable threatened category at the global scale (IUCN, 2012). Note that we had no intention of assessing risk. This procedure was only intended to minimize subjectivity in considering genera as widespread or restricted; thus, we only used IUCN criterion B2 to assess and categorize range size, without considering any of the other subcriteria needed for an IUCN risk assessment. We considered this proxy as highly conservative to identify restricted genera because the IUCN categories are intended for species classification and we were applying this threshold at the genus level.

All maps were elaborated with QGIS version 3.4.3‐Madeira (QGIS Development Team, 2021) using the shapefile that includes the third level of TDWG code of botanical countries.

### Climatic characterization of the AsPG

#### Climatic data collection and layer compilation

Climatic data were obtained for all the cleaned records in the AsPG database using two approaches: qualitative and semiquantitative. For the qualitative approach, all records from each genus were unequivocally classified as tropical or temperate according to the category assigned in Wen et al. (2001), which used the geographic range of each taxon, defined strictly using latitude. Given the broad‐scale relation between climate and latitude, we assume that these two categories (tropical and temperate), originally conceived as geographical, do actually reflect different climatic conditions as well.

For the semiquantitative approach, the records of the AsPG database were cross‐checked with five spatial layers representing five bioclimatic regionalizations of the world from analytical and synthetical classification systems that are frequently used in biology: (1) latitudinal zonation, that divides the world in four zones solely based on latitudes; (2) Köppen–Geiger classification (Köppen and Geiger, 1936), that recognizes six zones mainly based on temperature and precipitation; (3) Holdridge classification (Holdridge, 1967), that identifies seven world life zones based on biotemperature (BioT; the temperature at which plants grow efficiently ([between 0°C and 30°C]); (4) global environmental stratification system (GEnS; Metzger et al., 2012), that recognizes seven broad biomes based on 42 bioclimatic variables; and (5) ecoregion system (Olson et al., 2001), that divides the world in 14 biomes based on environmental conditions and the biogeographical information of the world's flora and fauna. To make the genera's climatic characterizations comparable between the most analytical classification systems (GEnS classification and ecoregion system) and the most synthetic ones (latitudinal zonation, Köppen–Geiger, and Holdridge classifications), we selected the broadest hierarchical category of each classification system as our analytical scale. To cross our database with each classification system, we needed to adapt three geospatial layers already available (ecoregion: Dinerstein et al., 2017; Köppen–Geiger: Beck et al., 2018; GEnS: Metzger et al., 2012) and to create two new layers for the remaining classification systems (latitudinal and Holdridge classifications).

To adapt the Köppen–Geiger layer, we used the improved version to 1‐km resolution of the traditional classification of Köppen–Geiger developed by Beck et al. (2018). We grouped the second and third levels of climatic categories to only consider the main classes: tropical (A), dry (B), temperate (C), continental (D), tundra (E), and polar (F) (available in GitHub: https://github.com/NiDEvA/Bioclimatic-classifications-AsPG.git; Coca‐de‐la‐Iglesia et al., 2021c). For GEnS classification, we used the layer including the global environmental zones provided by Metzger et al. (2012) to obtain the seven broad biomes and transformed the resulting layer to tif format (available in GitHub: https://github.com/NiDEvA/Bioclimatic-classifications-AsPG.git; Coca‐de‐la‐Iglesia et al., 2021c). In the case of the ecoregions system, we used the biomes delimitation of the shapefile provided by Dinerstein et al. (2017). Then, considering two new inclusive categories that gathered together some of the original categories recognized, we simplified this classification in R (hereafter “simplified ecoregions”) (Dinerstein et al., 2017). As a result, we created the tropical and subtropical category that unified four original biomes (tropical and subtropical dry broadleaf forests, tropical and subtropical moist broadleaf forests; tropical and subtropical coniferous forests; and tropical and subtropical grasslands, savannas, and shrublands) and the temperate category that included three original biomes (temperate broadleaf and mixed forests; temperate conifer forests; and temperate grasslands, savannas, and shrublands), and transformed the resulting layer to tif format (available in GitHub: https://github.com/NiDEvA/Bioclimatic-classifications-AsPG.git; Coca‐de‐la‐Iglesia et al., 2021c).

The new layer created to represent the latitudinal zonation was built using 23.5° as the geographical limit between tropical and subtropical zones, 40° for subtropical and temperate and 66.5° for temperate and polar, both in northern and southern hemispheres (available in GitHub: https://github.com/NiDEvA/Bioclimatic-classifications-AsPG.git; Coca‐de‐la‐Iglesia et al., 2021c). We chose 40° as the limit of the subtropics following the most common criterion among physical geographers (Corlett, 2013) and to be consistent with the delimitation of the remaining bioclimatic zones recognized by latitude. To build the new layer for the Holdridge classification, we estimated BioT from the 12 layers including the average monthly temperature from 1970 to 2000 at a 30‐sec resolution available in WorldClim (Fick and Hijmans, 2017; https://www.worldclim.org/). First, we reclassified each layer to represent BioT by setting to 0 all temperatures below 0°C and above 30°C and keeping the original temperature value between 0°C and 30°C. With the 12 reclassified monthly layers we created a new layer that contained the mean of BioT. Finally, we classified BioT values from the mean BioT layer according to the latitudinal World Life zones recognized by Holdridge (Holdridge, 1967): tropical (30–24° BioT), subtropical (24–17° BioT), warm temperate (17–12° BioT), cool temperate (12–6° BioT), boreal (6–3° BioT), subpolar (3.5–1.5° BioT), and polar (1.5–0° BioT) available in GitHub: https://github.com/NiDEvA/Bioclimatic-classifications-AsPG.git; Coca‐de‐la‐Iglesia et al., 2021c).

Once the five layers were prepared, they were crossed with the spatial database of the AsPG to assign each record of the database to the corresponding climatic category according to each classification system.

#### Climatic characterization of genera

We provided the characterization of the climatic preferences of the AsPG at two levels (genus and whole AsPG clade). For each level, the climatic characterization of taxa was done following a qualitative approach for the classification system that uses the two geographical categories recognized by Wen et al. (2001) and a semiquantitative approach for the remaining five bioclimatic classification systems. For the climatic characterization of genera in the qualitative approach, each genus was unequivocally considered as tropical or temperate in terms of climatic preferences, as herein inferred from their latitudinal range (see above, Wen et al., 2001). For the five classification systems analyzed with the semiquantitative approach, we computed the percentage of records classified within any given category. To avoid overestimations due to taxonomic sampling bias, percentages for the AsPG climatic characterization at the clade level were done using a reduced database. In this reduced database, all oversampled genera (>1500 records) were each represented by 1000 records regularly selected with the function spsample in the package sp (Pebesma et al., 2021) in R version 3.5 (R Core Team, 2018). Pie charts were then generated from this reduced database for each bioclimatic classification using ggplot2 package (Wickham et al., 2021) in R version 3.5 (R Core Team, 2018). To characterize the climatic preference of each genus, we estimated the percentage of occurrences classified in each category per genus per classification system. To avoid overestimations due to geographical sampling bias, we estimated percentages by only retaining one record per coordinate per genus.

## RESULTS

### Spatial representativeness of the AsPG database

**Figure 1:**
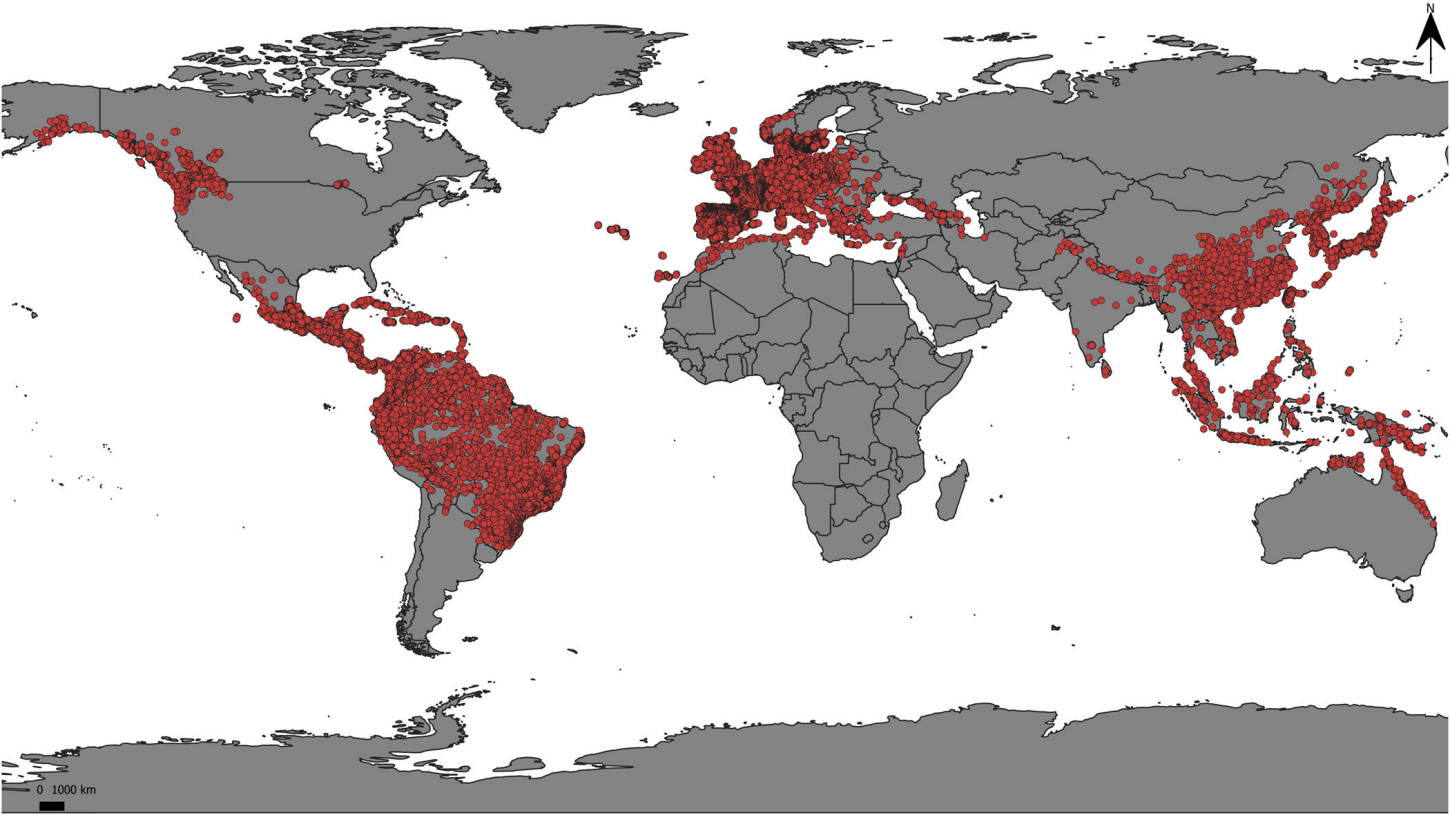
Point‐occurrence map of global distribution of Asian Palmate Group of Araliaceae.

The database represents all of the genera known to belong to the AsPG clade, including the six recently reinstated or newly described genera segregated from the polyphyletic genus *Schefflera*. The database contained 476,704 records resulting from the cleaning process of the initial database of 683,207 observations and covered the worldwide distribution range of the clade (Coca‐de‐la‐Iglesia et al., 2021a; Figure 1). The data cleaning process resulted in losses from 5% (*Metapanax* J. Wen & Frodin) to 71% (*Cephalopanax*) of records per genus (Table 2). *Hedera* is the genus with the most occurrences (401,252; Table 2), followed by *Oreopanax* Decne. & Planch. with 20,108 (4%; Table 2), and *Dendropanax* with 13,600 records (3%; Table 2). The genera with the fewest records are *Cephalopanax* (28 occurrences; Table 2) and *Frodinia* (48 occurrences; Table 2). The genus with the highest sampling effort is *Hedera* with a maximum of 8093 records per cell, while the genera with the lowest sampling effort are *Cephalopanax* and *Heteropanax* with a maximum of six records per cell in both cases (Appendix S1). Generic point maps encompassed the distributional range of each genus (Appendix S2) and revealed that, in 12 genera, all the botanical countries of their native range were represented, while the sampling incompleteness of the remaining 11 genera varied between 3% (in *Didymopanax*) and 50% (in *Metapanax*, for which half of the botanical countries of the natural range were missing).

**Table 2:** Spatial information for genera of the Asian Palmate Group (AsPG) of Araliaceae. NSpp: number of accepted species for each genus. Range: distribution range. Ndow: number of records downloaded. Nred: number of records kept after the data cleaning. Loss: percentage of data lost during data cleaning. RepGen: percentage of records per genus in the AsPG database. RepSpp: percentage of species recorded per genus in the AsPG database. NBcountry: number of botanical countries of the natural range of each genus. SamInc: sampling incompleteness, estimated as the percentage of botanical countries of the natural distribution of each genus not represented in the AsPG database. AOO: area of occupancy in km^2^. EOO: extent of occurrence in km^2^.

Genus	NSpp	Range	Ndow	Nred	Loss (%)	RepGen (%)	RepSpp (%)	NBcountry	SamInc (%)	AOO	EOO
*Brassaiopsis*	48	Asia	1062	953	10.26	0.20	64.58	21	9.52	1108	9.85E+06
*Cephalopanax*	3	South America	98	28	71.43	0.01	100.00	2	0.00	88	1.74E+05
*Chengiopanax*	2	Asia	1178	1072	9.00	0.22	100.00	3	0.00	2864	3.77E+06
*Crepinella*	33	South America	1115	788	29.33	0.17	96.97	7	0.00	1292	5.71E+06
*Dendropanax*	103	Asia; Central, South Americas	21,914	13,600	37.94	2.85	91.26	51	7.84	22,132	2.32E+08
*Didymopanax*	37	Central, South Americas	10,483	8788	16.17	1.84	100.00	32	3.13	17,552	2.11E+07
*Eleutherococcus*	38	Asia	5704	4704	17.53	0.99	76.32	21	4.76	6796	1.35E+07
*Fatsia*	3	Asia	2826	877	68.97	0.18	66.67	6	0.00	2068	1.28E+06
*Frodinia*	2	Central America	54	48	11.11	0.01	100.00	3	0.00	92	4.50E+04
*Gamblea*	4	Asia	1112	955	14.12	0.20	100.00	13	23.08	2188	1.06E+07
*Hedera*	12	Europe, North Africa, Asia	57,6291	40,1252	30.37	84.17	100.00	68	7.35	190,564	4.95+07
*Heptapleurum*	317	Asia, North Oceania	8201	6371	22.31	1.34	58.68	34	8.82	9632	4.03E+07
*Heteropanax*	10	Asia	228	168	26.32	0.04	70.00	15	33.33	328	3.22E+06
*Kalopanax*	1	Asia	2117	1611	23.90	0.34	100.00	10	0.00	3808	6.30E+06
*Macropanax*	17	Asia	471	392	16.77	0.08	70.59	17	23.53	908	1.02E+07
*Merrilliopanax*	3	Asia	170	158	7.06	0.03	100.00	6	33.33	184	3.02E+05
*Metapanax*	2	Asia	938	894	4.69	0.19	100.00	6	50.00	568	1.20E+06
*Oplopanax*	3	Asia, North America	4289	3941	8.11	0.83	100.00	14	0.00	6200	1.29E+07
*Oreopanax*	148	Central, South Americas	30,168	20,108	33.35	4.22	97.30	35	2.86	18,980	2.58E+07
*Sciodaphyllum*	138	Central, South Americas	12,614	8874	29.65	1.86	92.75	14	0.00	8512	8.32E+06
*Sinopanax*	1	Asia	119	111	6.72	0.02	100.00	1	0.00	288	1.30E+04
*Tetrapanax*	1	Asia	1676	716	57.28	0.15	100.00	4	0.00	880	2.26E+06
*Trevesia*	11	Asia	367	293	20.16	0.06	90.91	17	23.53	688	9.30E+06

### Spatial patterns in the AsPG

**Figure 2:**
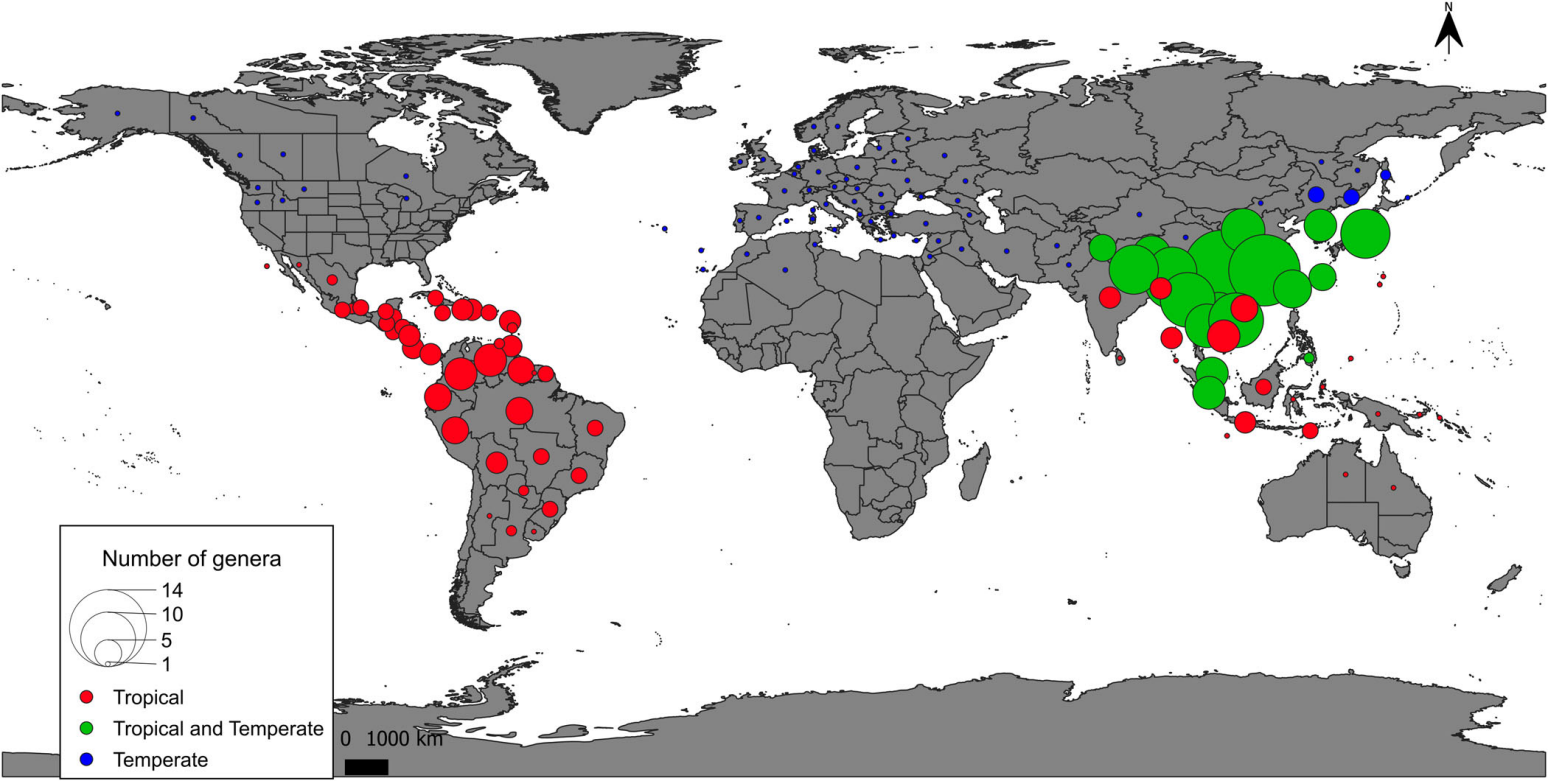
Biodiversity map of the Asian Palmate Group of Araliaceae as estimated from the number of genera per botanical country. Colors represent the climatic preferences of the genera recorded in each botanical country and according to latitudinal ranges recognized by Wen et al. (2001): Blue represents botanical countries where only temperate genera have been recorded, red where only tropical genera have been recorded, and green where temperate and tropical genera have been recorded.

The analysis of spatial diversity in the AsPG revealed that the number of genera is unevenly distributed across the world (Figure 2) with Southeast Asia as the area with the highest number of genera per botanical country, followed by Central and South America. Europe and North America have the lowest diversity with only one genus per botanical country (Figure 2).

**Figure 3:**
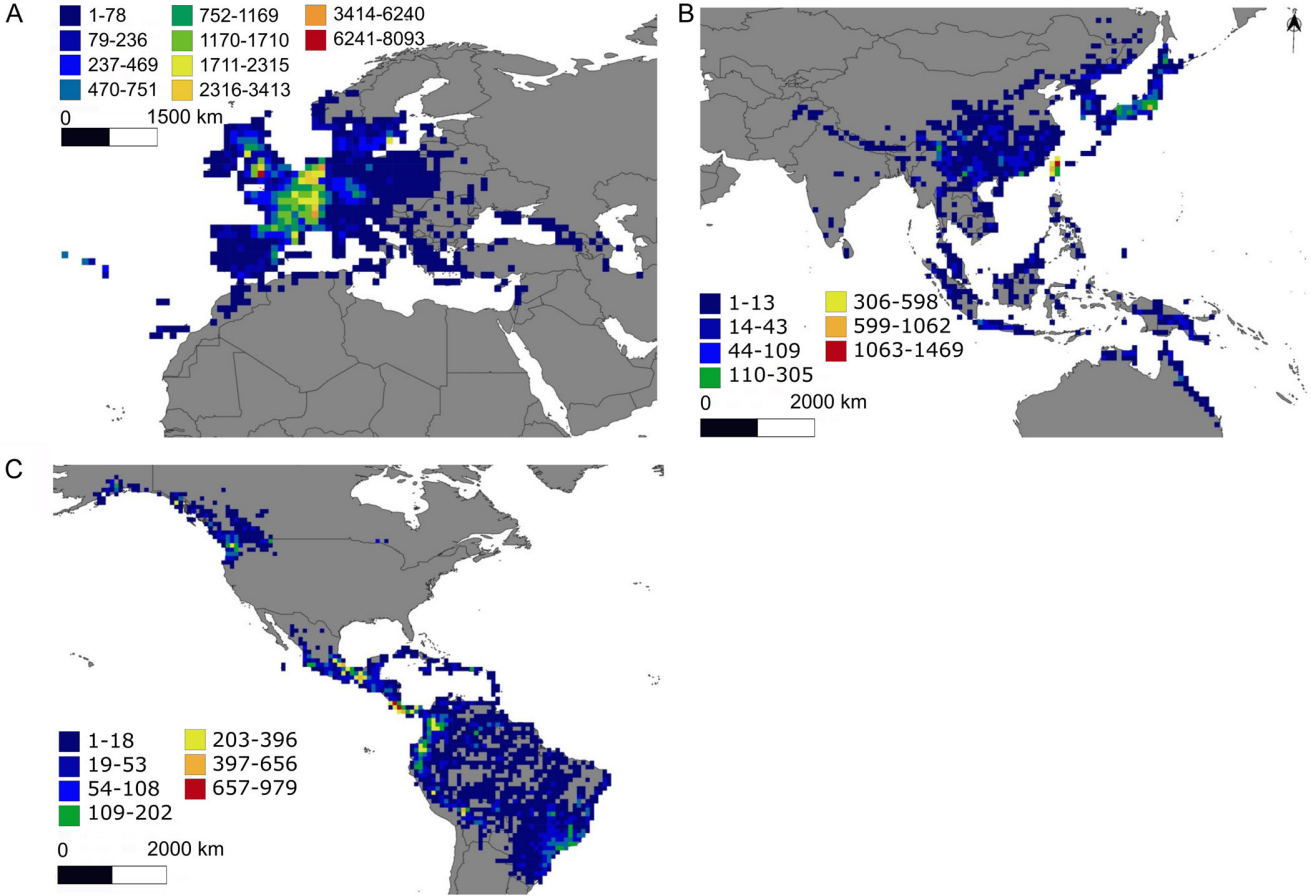
Heat maps of the Asian Palmate Group of Araliaceae based on the total number of point occurrences per cell (one geographical degree of longitude and latitude). Cell colors are based on a gradient scale established by Jenks natural breaks, ranging from blue (fewest occurrences per area) to red (most occurrences per area). (A) Europe (including North Africa), with a hotspot area in the center of the continent. The last four cell categories (from pale yellow with 1710 to 2315 records, to red with 6240 to 8093 records) were identified as sampling hotspots (cells with 25% of the maximum sampling effort per cell per region, see Materials and Methods). (B) Asia (including Oceania), with two main hotspot areas on islands (Taiwan and Honshu [Japan]). The last three cell categories (from yellow with 305–598 records, to red with 1620–1469 records) were identified as sampling hotspots. (C) Americas, with four main hotspot areas from North America to Colombia. The last cell three categories (from yellow with 202–396 records, to red with 656–979 records) awee identified as sampling hotspots.

Also, the number of AsPG observations was unevenly distributed across the world (Figure 3). The region of the world with the highest number of observations per area was Europe (only represented by *Hedera*) with a maximum number of observations per cell of 8093. Most of the European sampling hotspots (i.e., cells with at least 2024 records, that is, 25% of the maximum sampling effort per cell in that area), are from central Europe (western England, France, Belgium, and the Netherlands; Figure 3A). The second region in terms of observations per area was Asia (maximum number of observations per cell: 1469) where two sampling hotspots were identified (Taiwan and central Japan, Figure 3B). The regions of the world with the fewest records were North and South America, which combined had a maximum number of observations per cell of 979 (Figure 3C). The American sampling hotspots were concentrated in four main areas within the continent. Two of these hotspot areas were in tropical North America, one in southern Mexico and the other one in Panama to Costa Rica. The third main hotspot area was in South America, extending from western Ecuador to western Colombia. The fourth American hotspot area was in North America (western Washington State), where there is only one genus [*Oplopanax* (Torr. & A. Gray) Miq.] belonging to the AsPG clade.

**Figure 4:**
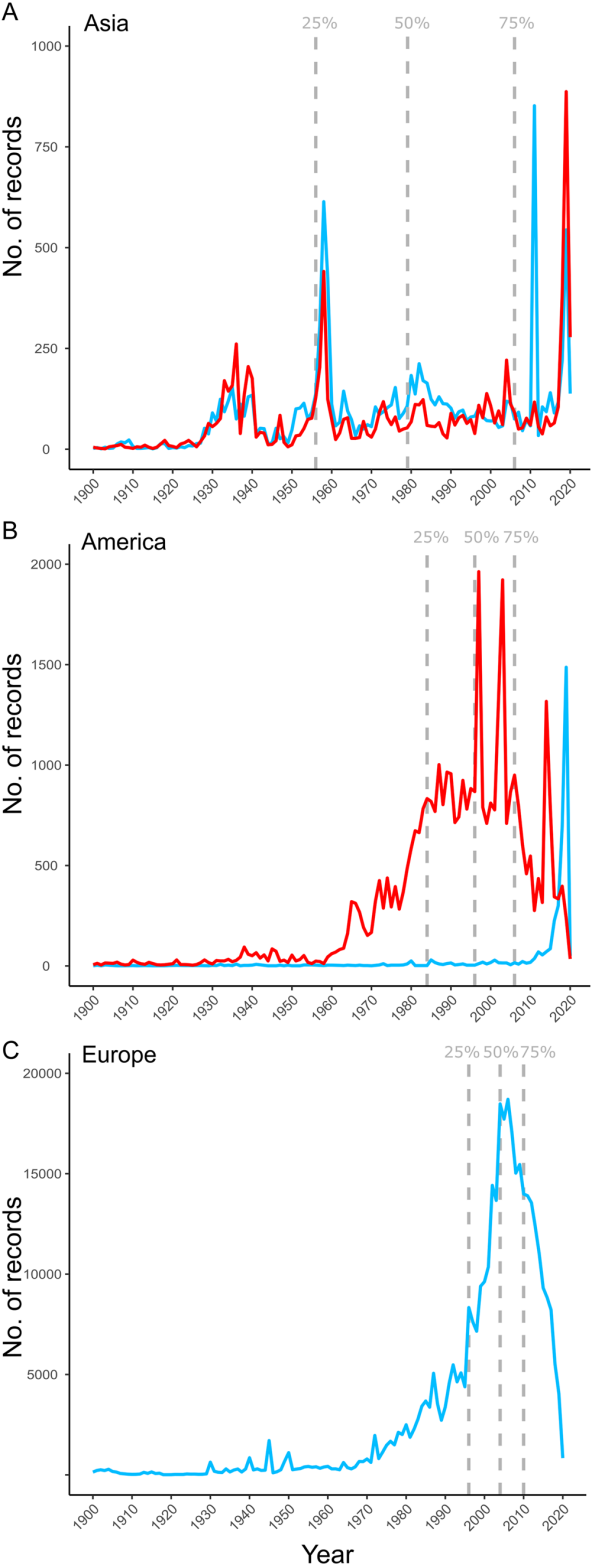
Temporal changes in the number of records (occurrences) of the Asian Palmate Group of Araliaceae database between 1900 and 2020 by regions. Colors represent the temporal tendencies detected in the sampling records of temperate (blue) or tropical (red) genera according to Wen et al. (2001). Dashed vertical lines indicate the cumulative frequencies of 25, 50, and 75 percentages of the total data. (A) Cumulative temporal series in Asia (including Oceania). (B) Cumulative temporal series in the Americas. (C) Cumulative temporal series in Europe (including northern Africa), where only the temperate genus Hedera occurs.

Finally, the analyses of sampling patterns across time and space revealed differences in the number of collections per year between temperate and tropical environments and among regions of the world (Figure 4). In Asia, the number of baseline collections per year remained constant through time, with three increases both for tropical and temperate genera according to the latitudinal simplification of Wen et al. (2001; Figure 4A). In the Americas, the number of collections across time had very different temporal patterns depending on whether the genera are tropical or temperate (Figure 4B). Most of the temperate collections (only represented by *Oplopanax*) were concentrated in the second decade of the 21st century (2015–2020), whereas a relatively constant high baseline was observed for collections of the tropical genera during the last decades of the 20th century, with isolated increases concentrated in the first decades of the 20th century. Finally, in Europe, where only one temperate genus (*Hedera*) occurs, there was an increase of collections since 1990 with a peak in the first decades of the 21st century (Figure 4C).

### Comparison of world bioclimatic regionalizations

The regionalization of the world is very different depending on the bioclimatic classification system used (Appendix S3). These differences are not evenly distributed across the world or the climatic categories. Indeed, the regionalization of certain areas, such as the low latitudes of the northern hemisphere are very similar among classifications, whereas the areas of the world that are classified as subtropical (when this category is recognized; Holdridge, GEnS, and latitudinal classifications) are either considered tropical, temperate or dry in the remaining classification systems, which do not include the subtropical category (Appendix S3). Besides, the delimitation of the areas considered as tropical or temperate shows major differences even when classifications that do not use the subtropical category are compared. Despite these major differences, there are two main spatial patterns in the regionalizations of the world across regions that emerge in all the classification systems used. First, the bioclimatic regions of the New World (particularly North America) and Australia extend over large continuous geographical areas describing a clear spatial pattern, either latitudinal or a combination of latitudinal and longitudinal in the classifications that recognize a dry category (Appendix S3). Secondly, for the rest of the world, the bioclimatic regions are patchy and extend over discontinuous geographical areas with no clear latitudinal or longitudinal pattern (Appendix S3).

### Spatial and climatic characterization of the AsPG

The spatial characterization of the generic distributions resulted in the identification of 11 restricted genera [*Brassaiopsis* Decne. & Planch., *Cephalopanax*, *Crepinella*, *Frodinia*, *Heteropanax*, *Macropanax*, *Merrilliopanax* H.L. Li, *Metapanax*, *Sinopanax* H.L.Li, *Tetrapanax* (K. Koch) K. Koch, and *Trevesia*; Table 2; Appendix S2] and four widespread genera with areas of occupancy larger than 10,000 km^2^ (*Dendropanax*, *Didymopanax*, *Hedera*, and *Oreopanax*; Table 2).

**Figure 5:**
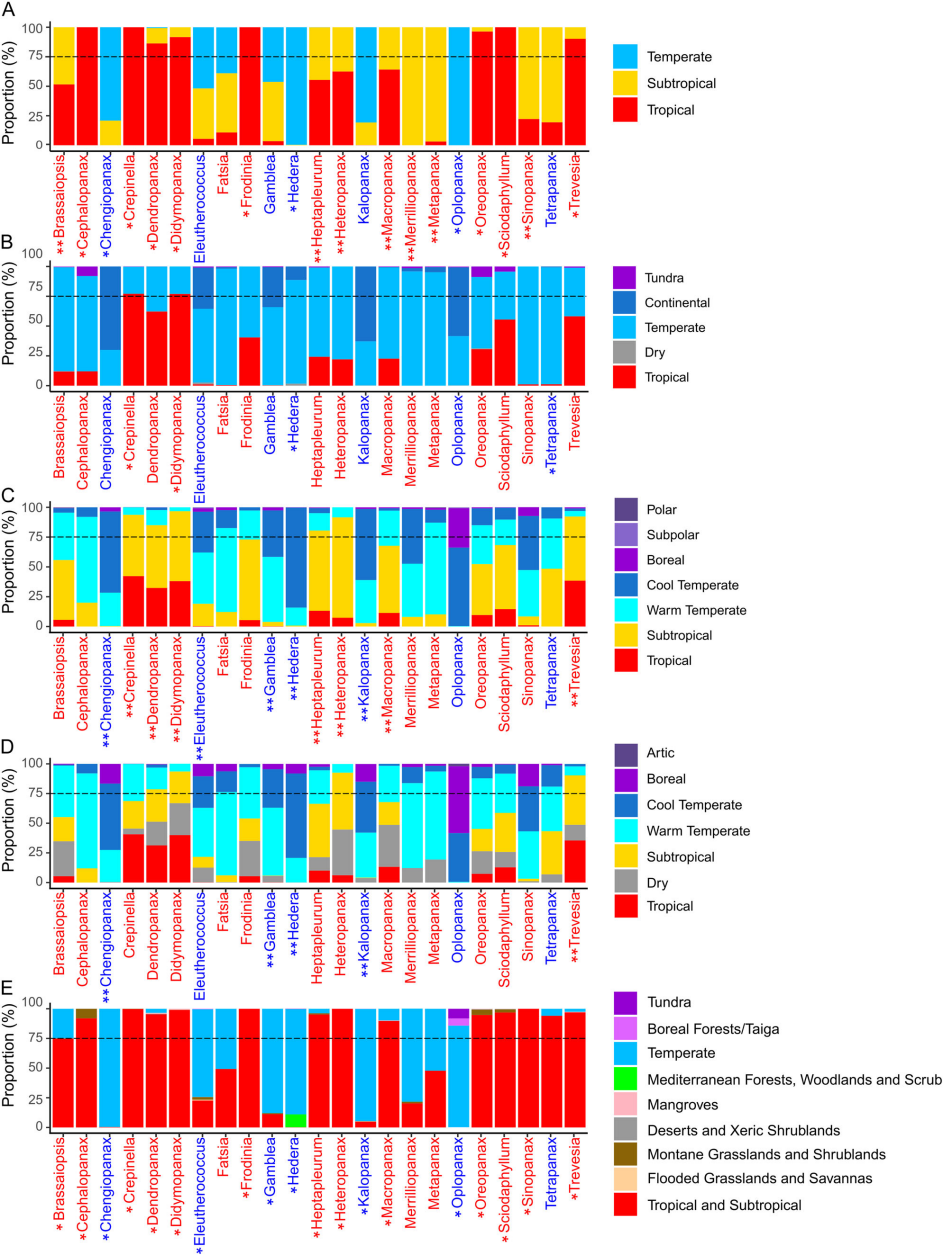
Climatic characterization of the 23 genera of the Asian Palmate Group (AsPG) of Araliaceae according to five bioclimatic regionalization systems of the World. The names of AsPG genera in the first axis are colored as done by Wen et al. (2001; tropical in red and temperate in blue). One asterisk indicates the genera for which the classification system assigned a climatic categorization was congruent with the one based on the geographical distinction by Wen et al. (2001). Two asterisks indicate congruence with the tropical or temperate climatic categories inferred from the results of Wen et al. (2001) when unifying the subtropical and the tropical proportions or the cold temperate and warm temperate proportions in the classification system. The 75% line is a visual guide to identify when a genus is considered as unequivocally assigned to one category or not. (A) Latitudinal zonation. (B) Köppen–Geiger classification based on temperature and precipitation (Köppen and Geiger, 1936; Beck et al., 2018). (C) Holdridge classification based on biotemperature (Holdridge, 1967). Congruence with the temperate category of Wen et al. (2001) was determined by considering the cold and warm temperate categories together and is denoted with two asterisks. (D) Global environmental stratification (GEnS) classification based on 42 bioclimatic variables (Metzger et al., 2012). Congruence with the temperate category of Wen et al. (2001) was determined by considering the cold and warm temperate categories together and is denoted with two asterisks. (E) Simplified ecoregion classification based on environmental conditions and the biogeographical information of the world's flora and fauna (modified from Dinerstein et al., 2017; see text for details).

The semiquantitative approach to characterize the climatic preferences of the AsPG genera revealed a general tendency for each genus to be classified in more than one climatic category in most of the classification systems analyzed (Figure 5). According to the most synthetic regionalization (latitudinal classification; Figure 5A), 15 genera had more than 75% of their occurrences classified in one category (hereafter “unequivocally assigned genera”): four as temperate (*Chengiopanax* C.B. Shang & J.Y. Huang, *Hedera*, *Kalopanax* Miq., and *Oplopanax*), eight as tropical (*Cephalopanax*, *Crepinella*, *Dendropanax*, *Didymopanax*, *Frodinia*, *Oreopanax*, *Sciadophyllum*, and *Trevesia*), and four as subtropical (*Merrilliopanax*, *Metapanax*, *Sinopanax*, and *Tetrapanax*). The remaining seven genera (*Brassaiopsis*, *Eleutherococcus* Maxim., *Fatsia* Decne. & Planch., *Gamblea*, *Heptapleurum*, *Heteropanax*, and *Macropanax*) had a large proportion of records assigned to two categories (four as tropical and subtropical, three as temperate and subtropical; Figure 5A). As the classification became more analytical, the number of genera unequivocally assigned to one category decreased to 10 in the Köppen–Geiger classification or to none in the Holdridge and the GEnS classifications. The only exception is the simplified ecoregions classification, in which 20 genera were unequivocally assigned to one category (Figure 5E). For the classifications that do not include the subtropical category, most of the equivocally classified genera were assigned to both the tropical and temperate categories (Köppen–Geiger: five, Simplified ecoregions: three; Figure 5B, E). For the Holdridge and GEnS classifications, which include the subtropical category, most of the genera that were classified in more than one category were indeed classified in the subtropical category as one of categories (Figure 5C,D).

Major differences were detected in the climatic preferences assigned to genera among the five classifications compared, with a large discrepancy compared to the preferences that were based on the latitudinal characterization by Wen et al. (2001; Figure 5). The classification system that revealed the most similarity with the one based on the latitudinal characterization by Wen et al. (2001) is the simplified ecoregions (Figure 5E), where 19 genera are included in the same category (six temperate, 13 tropical; see asterisks in Figure 5E). The classifications that depart the most are the other two most analytical ones (Holdridge and GEnS classifications), where no genus is included in a similar category (see genus with one asterisk in Figure 5C, D), while 12 or five similarities are recovered, respectively, when the cold temperate and warm temperate categories are unified (see double asterisks in Figure 5C, D). Then, the more synthetic classification of Köppen–Geiger revealed four genera in categories comparable to the classifications of Wen et al. (2001) (two tropical, two temperate; see genera with one asterisk in Figure 5B). In between, the latitudinal classification recovers 11 similarities (three temperate, eight tropical; see genera with one asterisk in Figure 5A) or 18 if the subtropical category is unified with the tropical one (see double asterisks in Figure 5A).

**Figure 6:**
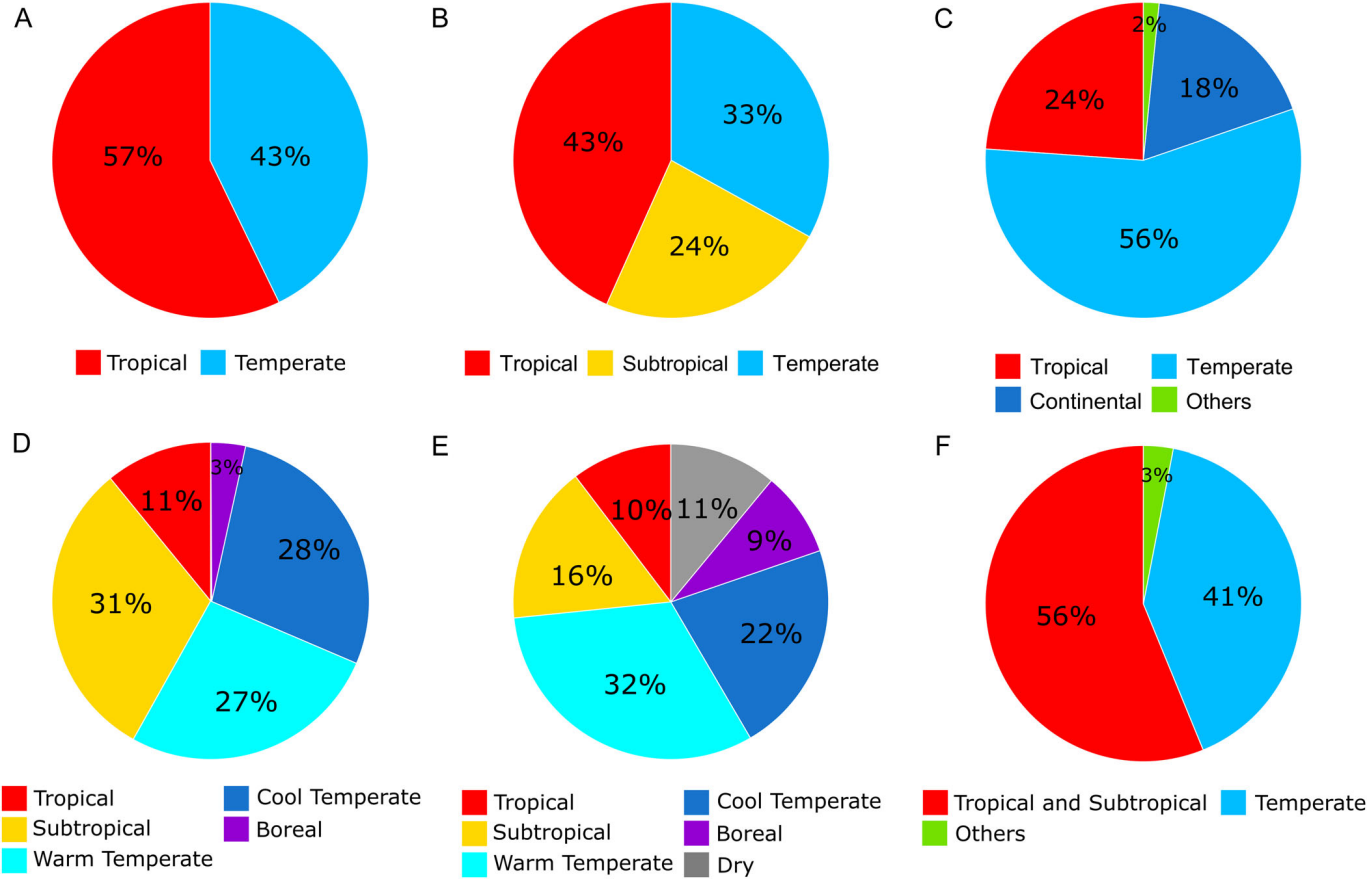
Climatic characterization of the Asian Palmate Group of Araliaceae according to five bioclimatic regionalization systems of the world and the one based on the geographical classification of Wen et al. (2001). Categories represented by less than 0.1% of the total records are not shown. Categories represented by less than 1% of the total records are grouped as the undefined category Others. (A) Latitudinal simplification of ranges (Wen et al., 2001). (B) Latitudinal zonation. (C) Köppen–Geiger classification based on temperature and precipitation (Köppen and Geiger, 1936; Beck et al., 2018). (D) Holdridge classification based on biotemperature (Holdridge, 1967). (E) Global environmental stratification (GEnS) classification based on 42 bioclimatic variables (Metzger et al., 2012). (F) Simplified ecoregion classification based on environmental conditions and the biogeographical information of the world's flora and fauna (modified from Dinerstein et al., 2017; see text for details).

When projected in a spatial context, the areas of the world where the AsPG occurs are recognized as different bioclimatic categories depending on the classification used (compare Figure 1 and Appendix S3). Indeed, the percentage of AsPG records assigned to each category varied largely based on the classification analyzed (Figure 6). For example, when classifications that recognize the subtropical category were compared, the percentage of occurrences assigned to the tropical category varied from only 10% according to GEnS classification (Figure 6E) to 43% in the latitudinal zonation (Figure 6B), or between 33% (latitudinal zonation, Figure 6B) and 55% (Holdridge classification, Figure 6D) for the temperate category. Also, the percentage of occurrences assigned to the subtropical category varied from 16% in GEnS classification (Figure 6E) to 31% in Holdridge (Figure 6D).

## DISCUSSION

### Worldwide background patterns in bioclimatic regionalizations as a tool to guide selection of the classification system

The huge amount of accessible digital information with the great advances in analytical models to perform ancestral reconstructions using phylogenies has made the use of quantitative climatic data more routine (e.g., Ogburn and Edwards, 2015; Albaladejo et al., 2021). However, to obtain accurate climatic characterization of taxa using quantitative approaches and to evaluate robustly evolutionary hypotheses resulting from such studies, high‐quality databases with high levels of spatial precision and good geographical coverage of the taxa are needed (Hortal et al., 2007, 2015; Meyer et al., 2016). Meeting these quality requirements is generally difficult due to the pervasive taxonomic and geographical gaps of knowledge that affect many lineages across the tree of life (Meyer et al., 2016). Thus, for these lineages, the use of qualitative climatic characterizations is still the best approach to minimize errors in reconstructions of the evolution of climatic niches. Of these qualitative approaches, the criterion or criteria used to categorize the taxa should be carefully selected because the application of different criteria may result in very different conclusions (Feeley and Stroud, 2018). However, the specific climatic classification selected and the procedure used to categorize the taxa are rarely stated in evolutionary studies (Feeley and Stroud, 2018, but see for example, Gagnon et al., 2019). Indeed, very often ad hoc qualitative characterizations are done based on undescribed procedures (Drescher et al., 2013) or on rough proxies, such as assigning latitudinal categories based on imprecise information on taxa distribution (Kozak et al., 2008). The comparison of the world regionalization maps herein compiled (Appendix S3) revealed large differences in the delimitation of the climatic regions in the world, which confirms previous findings for the tropics (Feeley and Stroud, 2018) and variability found in the delimitation of the subtropics (Corlett, 2013). We are aware that some part of the differences detected among regionalizations rests on the fact that each classification system is based on a different set of variables and recognizes a different number of categories. However, we argue that a sizeable part of these differences is due to the fact that we are using categorical classification systems for climate when the nature of the climate is dynamic and transitional. Therefore, if we are using categorical classifications, we should explicitly consider how well the available classification systems capture the transitional nature of climate for the area of the study group.

To do so, we can take advantage of the geographical patterns that emerge from an examination of the similarities and differences detected among classifications across the world (see maps in Appendix S3). These geographical patterns allow us to identify the regions of the world in which the transitional nature of climate results in the delimitation of highly heterogeneous bioclimatic regions regardless of the classification choose. Therefore, when a quantitative alternative is not possible for the study group, the study of these world geographical patterns is highly useful to select the regionalization system that minimizes the inherent error of qualitative proxies to capture the transitional nature of climate. First, the delimitations of the bioclimatic zones at high latitudes in the northern hemisphere were highly similar among classification systems (compare polar in latitudinal classification [Appendix S3.1] to tundra and frost in Köppen–Geiger [Appendix S3.2], subpolar and polar in Holdridge [Appendix S3.3], arctic in GEnS [Appendix S3.4], and tundra in simplified ecoregions [Appendix S3.5]). This similarity among classifications reflects that the transitional nature of climate at these latitudes is very well captured in all classifications irrespective of the variables used or the categories recognized. As a result, if the study group is distributed in such latitudes (which is not our case, see next section), the selection of one classification system or another may be irrelevant since it may not result in significant differences on the climactic affinities attributed to the taxa. Secondly, the regionalizations for the New World (particularly for North America) and Australia, result in regular shapes that describe a clear latitudinal or latitudinal + longitudinal pattern when a “dry” category is recognized (Appendix S3). The bioclimatic zones delimited in these areas of the world are different among classifications, but they are compatible and continuous, regardless of which regionalization is used (Appendix S3). Thus, classification systems can eventually capture the variation in climatic preferences of taxa from these areas. In such cases, however, selection of the best‐fitting regionalization is key in obtaining robust conclusions and ultimately depends on the range of the taxa, the geographical scale of the study and the question addressed. Third, bioclimatic zones in the Old World are irregularly shaped and do not follow any clear spatial pattern. The impact of irregularly shaped regions on climatic regionalizations has already been analyzed (Aydin et al., 2021) and, as in our case study (see next section), result in the delimitation of highly patchy regions that are incompatible across different classification systems (Appendix S3). As a result, the use of qualitative proxies to characterize the climatic preference of taxa occurring in the Old World may be more difficult and may also lead to discrepancies in conclusions depending on the classification system chosen. Therefore, we argue that depending on the geographical scale of the study and the distribution of the study group within the Old World, we would disregard the use of qualitative proxies.

The fact that the major background conflicts detected among climatic classification systems are particularly concentrated in the areas of the world that are recognized as subtropical may point to the limitation of categorical classification systems in capturing the transitional nature of climate in these regions. In fact, this question may be partly responsible for the major differences in perspectives between biogeographers and phylogeneticists on what and where the tropics are (Feeley and Stroud, 2018) or on how we define the subtropics (Corlett, 2013). Consequently, these background conflicts on the delimitation of the tropics and the subtropics may also affect our delimitation and understanding of the temperate zones as well. Since the tropics are often simply interpreted as being the opposite of temperate regions to explain large scale biogeographic and diversity patterns (e.g., Wiens and Donoghue, 2004; Silva et al., 2021), we should be more cautious in the climatic characterization step in evolutionary studies and evaluate the different classifications by considering the distribution ranges.

### Spatial gaps of knowledge in the AsPG distribution

The spatial database compiled provides good taxonomic, geographical, and temporal coverage of the AsPG at the genus level (Coca‐de‐la‐Iglesia et al., 2021a; Appendix S2). However, the sampling effort is not even across the worldwide AsPG distribution, with the highest number of sampling hotspots detected in Europe and the lowest in Asia and the Americas (Figure 3). Similar geographical patterns that are biased toward a greater documentation of biodiversity in Europe have already been reported in other studies using digital accessible information (Boakes et al., 2010; Meyer et al., 2016). Geographical biases like these need to be considered to avoid reaching misleading conclusions regarding patterns of biodiversity (Soberón and Peterson, 2004; Boakes et al., 2010) and to design targeted sampling strategies that can fill these spatial and taxonomic gaps. In the case of the AsPG, the sampling bias displays a spatial pattern that is just the opposite of the biodiversity pattern. Indeed, Asia, which is the richest region in terms of genera, is the region characterized by the lowest sampling effort, while Europe, which is the region with the lowest diversity in terms of genera, has had the greatest sampling effort (Figures 2 and 3). As with many other cases, the AsPG mismatch between biodiversity documentation and biodiversity pattern may be attributed to factors unrelated to biodiversity, such as economic wealth, geographical accessibility, and lack of access to local databases (Hortal et al., 2007; Yesson et al., 2007; Amano and Sutherland, 2013; Yang et al., 2013; Wen et al., 2015, 2017). Furthermore, other non‐biodiversity factors may be involved in the mismatch pattern seen in the AsPG, including research strategies or biases introduced by the traditional use of certain species against others. Interestingly, a similar mismatch pattern is observed for the AsPG when downsizing the scale both in Europe and in Asia. Indeed, all European sampling hotspots are in Central Europe, where only one species of *Hedera* occurs, whereas southwestern Europe, which harbors the greatest diversity (including all the continental European species of *Hedera*; Valcárcel et al., 2003, 2017), emerged as an undersampled area. Similarly, the lowest sampling effort detected in Asia is in continental southwestern China (Figure 3B), which is the area with the greatest AsPG diversity at the generic level (Figure 2). At a global scale, the areas that should be targeted to fill the AsPG geographical gaps are located at the boundaries of certain botanical countries in Asia (Bangladesh, Nepal, Bhutan, and India; see *Brassaiopsis*, *Dendropanax*, *Eleutherococcus* Maxim., *Gamblea*, *Heteropanax*, *Macropanax*, *Merrilliopanax*, *Metapanax*, and *Trevesia*) and the Americas (French Guiana: *Dendropanax*; Surinam: *Oreopanax*; Appendix S1). At a regional scale, our targeted areas are in southwestern Europe for which the geographical coverage of our database remains poor.

### The climatic preferences of the AsPG: neither tropical nor temperate

Despite the gaps of knowledge described above, our spatial database provides a good taxonomic and geographical coverage of the AsPG genera to evaluate whether their tropical–temperate distributions are correlated with contrasting climatic preferences. The AsPG has traditionally been considered as a tropical clade since, according to earlier latitudinal interpretations of taxon ranges, 16 genera occur between the tropics of Cancer and Capricorn, while only seven are found in temperate zones (Plunkett et al., 1996; Wen et al., 2001). Our results indicate that this tropical vs. temperate latitudinal distinction is not correlated with a tropical–temperate climatic duality because most of the genera are classified in a combination of climatic categories that may, or may not, include these categories (Figure 5). Moreover, for most of the classification systems analyzed, the genera of the AsPG are classified within transitional categories between the tropical and temperate ones. Consequently, the AsPG clade cannot be considered as a tropical lineage in terms of climatic preferences, nor as predominantly showing contrasting tropical–temperate climatic affinities. This result does not contradict the tropical–temperate latitudinal transitions of the AsPG (Wen et al., 2001), but does contradict our assumption that the broad‐scale correlation between latitude and climate is reflected in contrasting climatic preferences for the genera of the AsPG.

First, the idea that each genus in the AsPG can be characterized by a single climatic condition representing a single category, which is implicitly assumed when identifying genera as tropical or temperate as we did in our qualitative approach in this work, is rejected when using our semiquantitative approach because most genera in the AsPG are classified in two or more categories when using more fine‐scale categorizations (Figure 5). This result is not the mere reflection of the scale finesse of the different classifications. Indeed, the latitudinal classification, that only recognizes three categories, classified 30% of the genera in more than one category, whereas for the most analytical classification analyzed (simplified ecoregions with 19 categories), 87% of the genera were unequivocally assigned to one category. In terms of climatic characterization, the classification of one genus in more than one category can be interpreted as evidence of a broad climatic niche or a poor performance of the categories in reflecting the climatic preferences of the genus. Broad climatic preferences are expected for taxa with wide ranges since geographical extent is correlated to realized climatic niches (Slatyer et al., 2013). However, in our case study, most of the genera are classified in more than one category in most of the classification systems (Figure 5) irrespective of the extent of their geographical ranges (Table 2). Indeed, assigned to more than one climatic category are the most widespread genera (*Didymopanax*, *Dendropanax*, *Oreopanax*; Table 2, Figure 5B–D) and most of the genera with much narrower distributions (e.g., *Cephalopanax*, *Frodinia* and *Merrilliopanax*; Table 2, see the semi Figure 5B–D). By contrast, some of the most widespread genera are unequivocally assigned to only one climatic category (*Hedera* or *Oplopanax*; Table 2, Figure 5). Because of this, the classification of most of the AsPG genera into two or more climatic categories each cannot be explained by range extent; thus, the classification cannot be a reflection of broad climatic niches. Alternatively, classifying one genus in more than one category can also be interpreted as evidence that the categories established in the classification systems do not fully capture the variability of the climatic preferences of the genus, but that does not mean that the classification is not robust or accurate enough. Instead, the regionalization of the world based on a given classification might not capture the climatic variability that affects the geographical range of the genus as would be the case of a genus that is distributed in a small area at the edge between two contiguous climatic regions. In this case, the genus would be classified in two categories, but the actual climatic niche cannot be considered broad. Indeed, we interpret that this is the case of several genera in our case study including *Cephalopanax*, *Frodinia*, and *Merrilliopanax* (Appendices S2 and S3). Most classification systems characterized these three genera as having preferences for two categories: subtropical and tropical, or subtropical and temperate. Given the small range of these genera and the fact that these pairs of categories are climatically contiguous in the classifications and spatially connected in world regionalizations, we attribute the amplitude of their climatic characterizations as a reflection of a poor performance of the classifications to capture the climatic variability in the transitional areas where the three genera occurred (Appendix S2).

Second, in most of the AsPG genera that are unequivocally classified in one given category, the category assigned (subtropical, warm temperate, cool temperate, continental, or boreal) is different from the tropical or temperate climatic categories we assumed from the geographical categories recognized by Wen et al. (2001). We believe that not all these discrepancies are attributable to the different definition of the climatic categories or the scale fineness of the classification systems since large agreement is detected between the ecoregions classification and our tropical–temperate characterization, when the two have different grounds (biogeographic realms and latitude, respectively) and a different scale of fineness (19 versus two categories, respectively). Most of these incongruences are due to assigning these genera to a subtropical or warm temperate category in the classification systems that recognize these categories (Holdridge, 1967; Metzger et al., 2012; Figure 5A, C–D). These two categories reflect intermediate and progressive climatic conditions between the ones that characterize the tropical and temperate or cool temperate categories (Holdridge, 1967; Metzger et al., 2012). Indeed, the regions of the world that are classified within these two intermediate categories (subtropical and warm temperate) coincide with areas that are primarily recognized as tropical or temperate in the other classifications (Appendix S3). Thus, we interpret that part of the inconsistencies detected among classifications for the climatic characterization of the AsPG genera derived from the limitations of the available world regionalizations to capture the transitional and dynamic nature of climate for the areas where taxa of the AsPG occur (see above).

In light of our results, we reached two conclusions. First, the climatic characterization of the AsPG genera has an inherent complexity due to the climatic variability of the geographic regions where they occur and the extent of their ranges. Second, the application of any classification system to characterize the climatic preferences of the AsPG genera may lead to spurious conclusions mostly because some of these genera occur in transitional climatic regions and the performance of categorical classifications to capture climatic variability in transitional regions is poor (see above). As a result, qualitative approaches do not sufficiently capture the preferences of 91% of the AsPG genera, which is reflected in large incongruences in the characterization of 20 genera across classifications. Indeed, only three genera displayed similar climatic preferences according to all the regionalizations analyzed (*Cephalopanax*, *Chengiopanax*, and *Hedera*; Figure 5). Qualitative approaches thus do not seem appropriate to address the climatic characterization of the AsPG genera.

As a consequence, our evolutionary hypotheses based on assuming a correlation between climate and geography at a broad scale for the tropical–temperate transition in the AsPG should be re‐evaluated (Valcárcel et al., 2014; Valcárcel and Wen, 2019). In these earlier studies, we hypothesized that the shift between tropical and temperate niches was a driving force in the early radiation of the AsPG that gave rise to the main tropical and temperate generic lineages. Although the present study was not an evolutionary study, our climatic results already suggest that we cannot attribute the acquisition of the temperate niche as playing a central role in the early evolution of the AsPG, since the AsPG genera do not have vastly contrasting climatic preferences (Figure 5). Indeed, in our study, there was only one regionalization that supported the characterization of the AsPG as a mainly tropical clade with a few temperate genera, which is an essential part of this hypothesis (simplified ecoregions; Figure 5E). Besides, this regionalization is based on biogeographic information of the world's flora and fauna, and thus, the tropical and temperate categories cannot be interpreted as purely climatic. Finally, in the remaining classification systems analyzed, most of the AsPG genera are classified within categories that represent intermediate climatic conditions between tropical and temperate, which rejects the assumption that the tropical–temperate distribution is correlated with contrasting climatic preferences in this clade. Given the largest named clade of Araliaceae, the AsPG, does not show a pure climatic tropical affinity, we wonder whether the tropical distribution of the whole family can be correlated with a tropical climatic affinity at a broad scale. Linking the tropical distribution of Araliaceae with tropical climatic preferences is essential to decouple the effect of climate from other biotic and abiotic historical and current processes and interpret the tropical–temperate latitudinal transitions in Apiales. A similar argument also could be made in other examples of tropical–temperate plant lineages, such as Vitaceae (Wen et al., 2018; Ma et al., 2021), Altingiaceae (Ickert‐Bond and Wen, 2013), *Prunus* (Hodel et al., 2021), and *Rhaphiolepis* (Liu et al., 2020), or animal lineages (see references in the introduction). Because the distribution and range extent of the lineages with tropical and temperate representatives are different, the re‐evaluations of the above cases may render very different results and can provide insightful information on our understanding of the latitudinal diversity gradient and its relation to climatic variables.

## AUTHOR CONTRIBUTIONS

N.G.M. and V.V. designed the study. M.C. compiled and curated the database and layers and did all the analyses with contributions from N.G.M. and V.V. J.W. reviewed the curated database. All participated in the writing of the manuscript.

## Supporting information


**Appendix S1**. Heat maps of Asian Palmate Group genera.

**Appendix S2**. Distribution maps of Asian Palmate Group genera.

**Appendix S3**. World regionalizations.

## Data Availability

The AsPG database used in this paper is available at Zenodo Repository: https://doi.org/10.5281/zenodo.5578149. The elevation layer of the world is available in Zenodo Repository: https://doi.org/10.5281/zenodo.5578234. The five spatial layers of the bioclimatic classifications used in this paper are available in GitHub Repository: https://github.com/NiDEvA/Bioclimatic-classifications-AsPG.githttps://github.com/NiDEvA/Bioclimatic-classifications-AsPG.git.
