# Supplementary material for: Evaluation of tropical–temperate transitions: An example of climatic characterization in the Asian Palmate group of Araliaceae
Source: Am J Bot. 2022 Sep 23;109(9):1488–507. doi: 10.1002/ajb2.16059 (PMC9826302; doi:10.1002/ajb2.16059)
Supplement: Supplementary file 2 — Appendix S2. Distribution maps of Asian Palmate Group genera. [file AJB2-109-1488-s001.pdf]

Appendix S2. Distribution maps of Asian Palmate Group genera

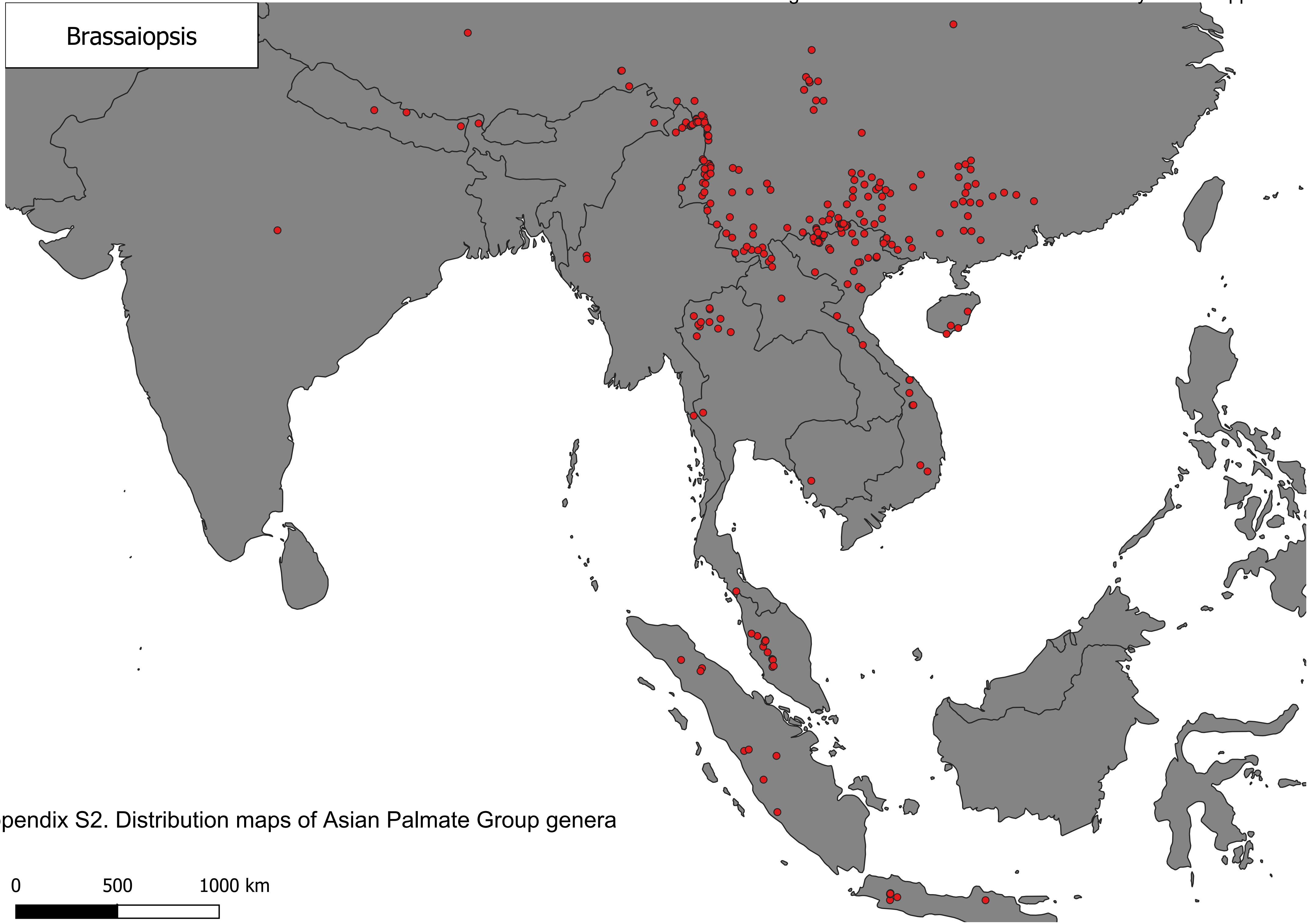

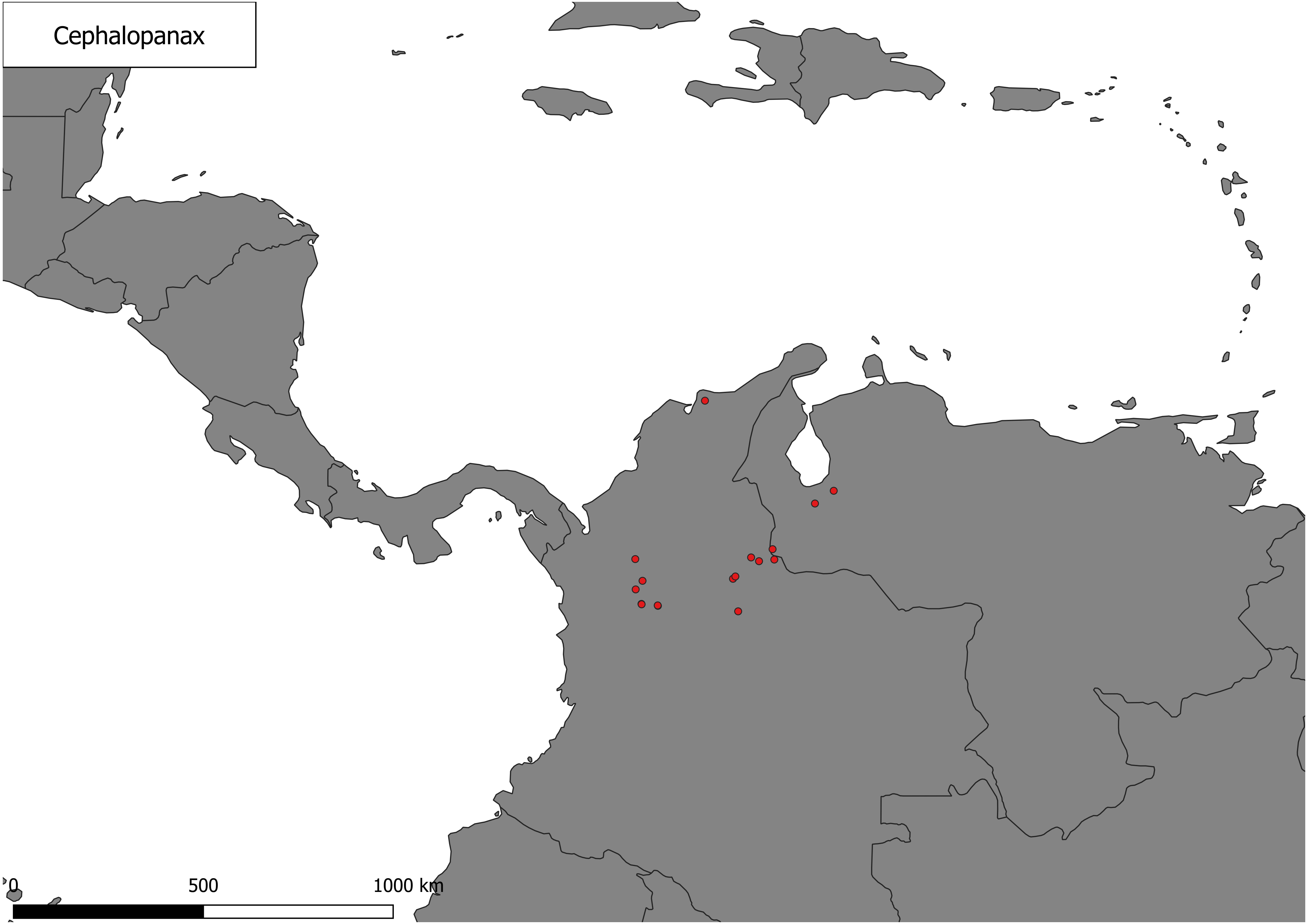
Cephalopanax
0
500
1000 km

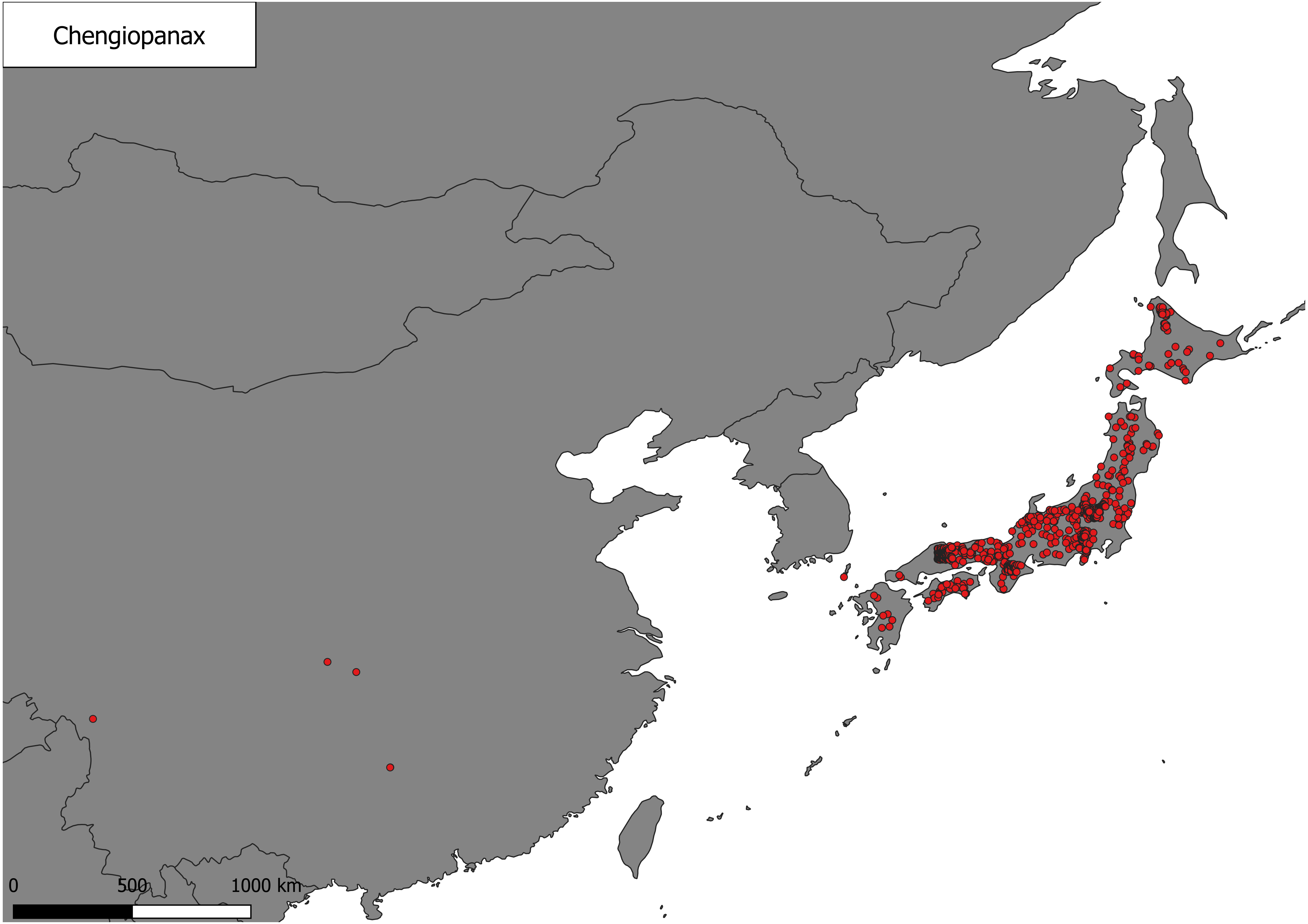
Chengiopanax
0
500
1000 km

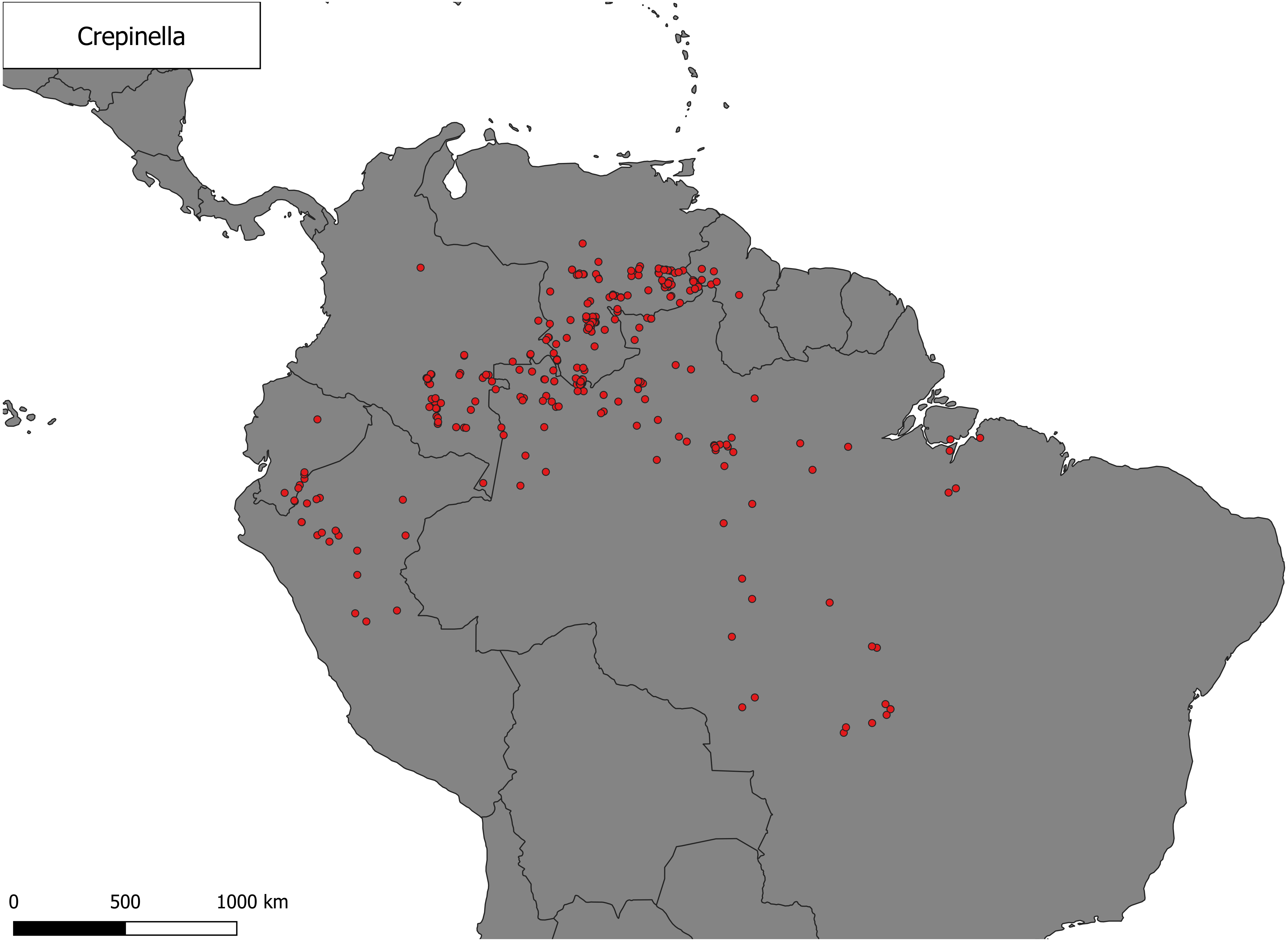
Crepinella
0
500
1000 km

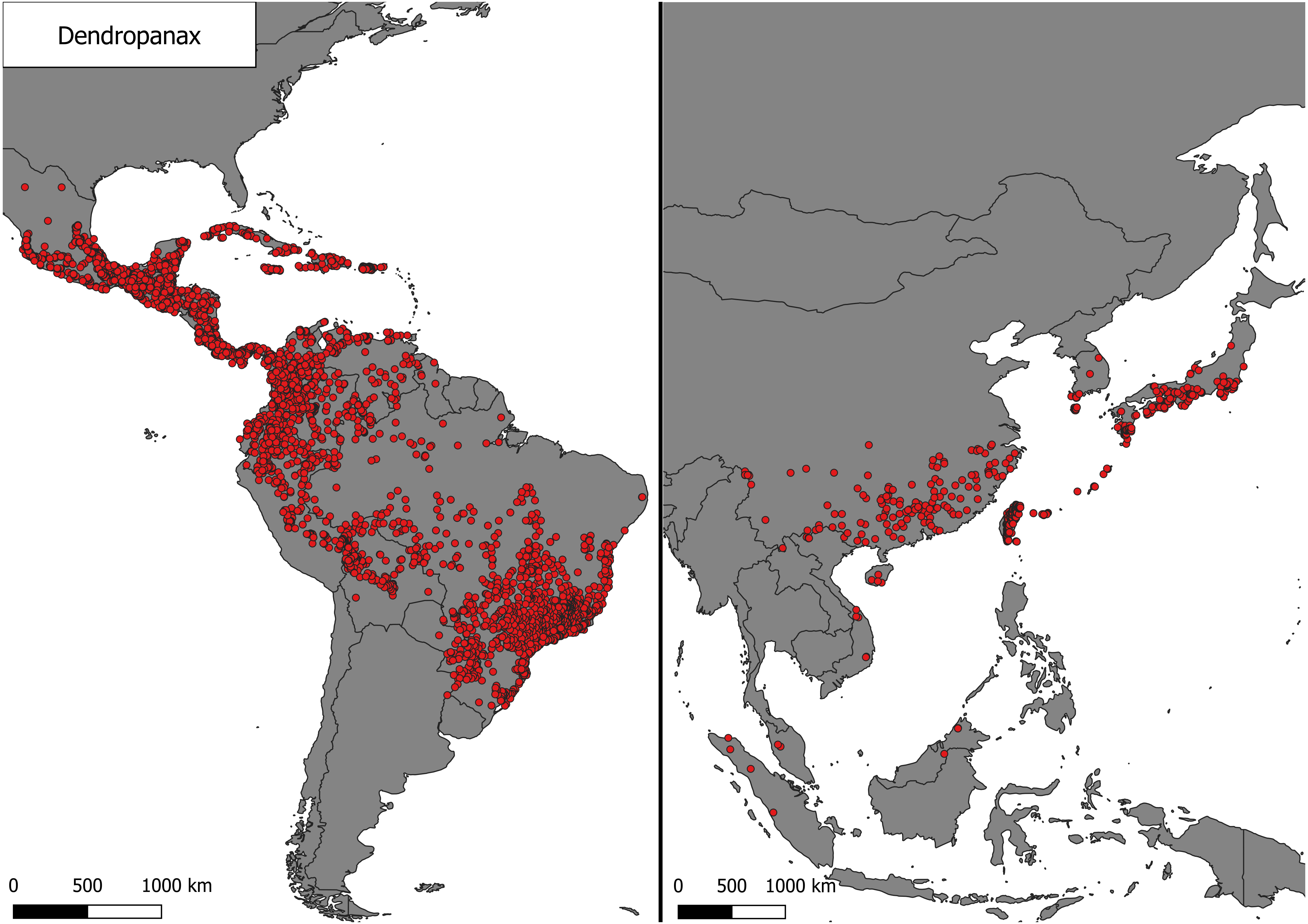

Dendropanax
0
500
1000 km
0
500
1000 km

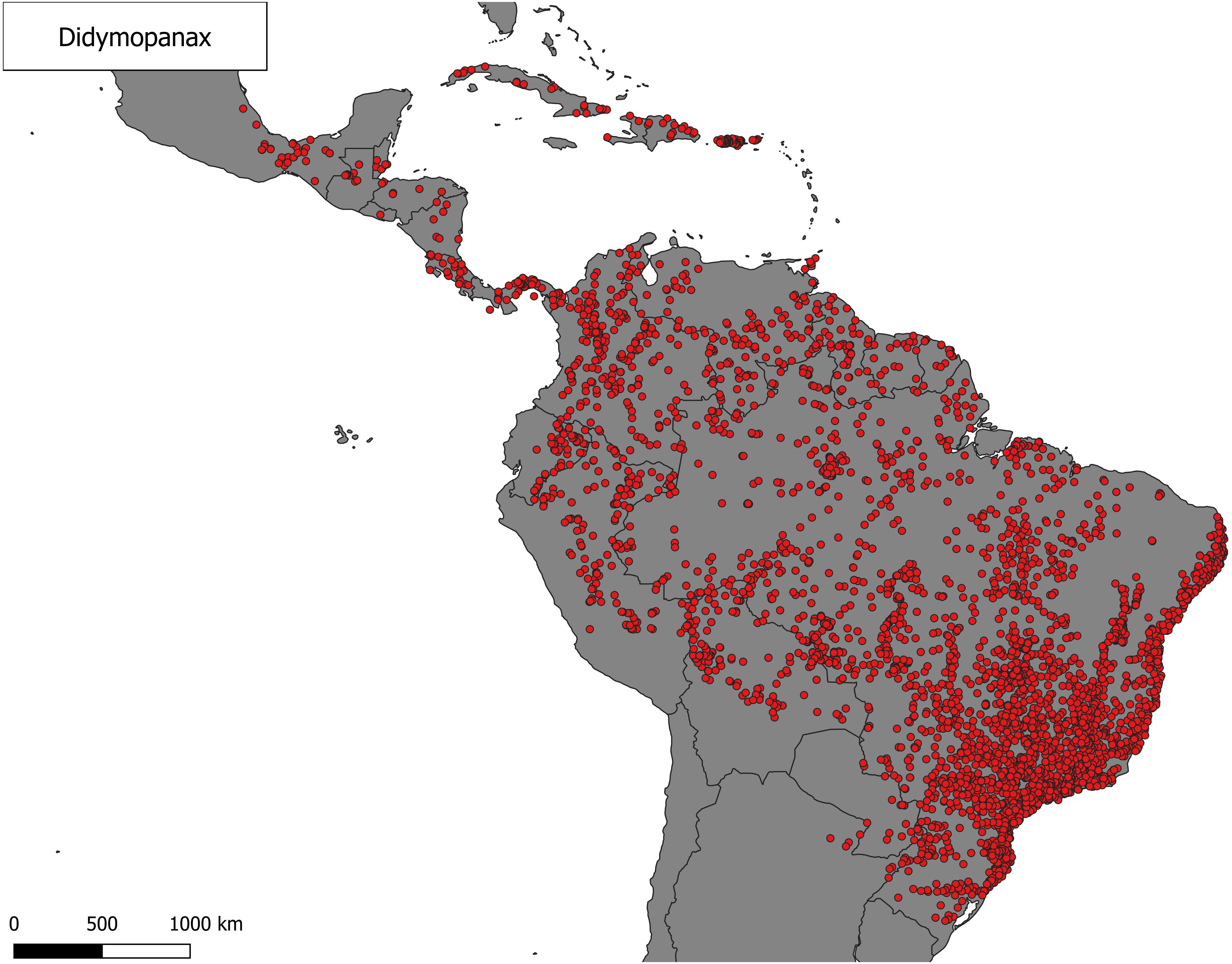
Didymopanax
0
500
1000 km

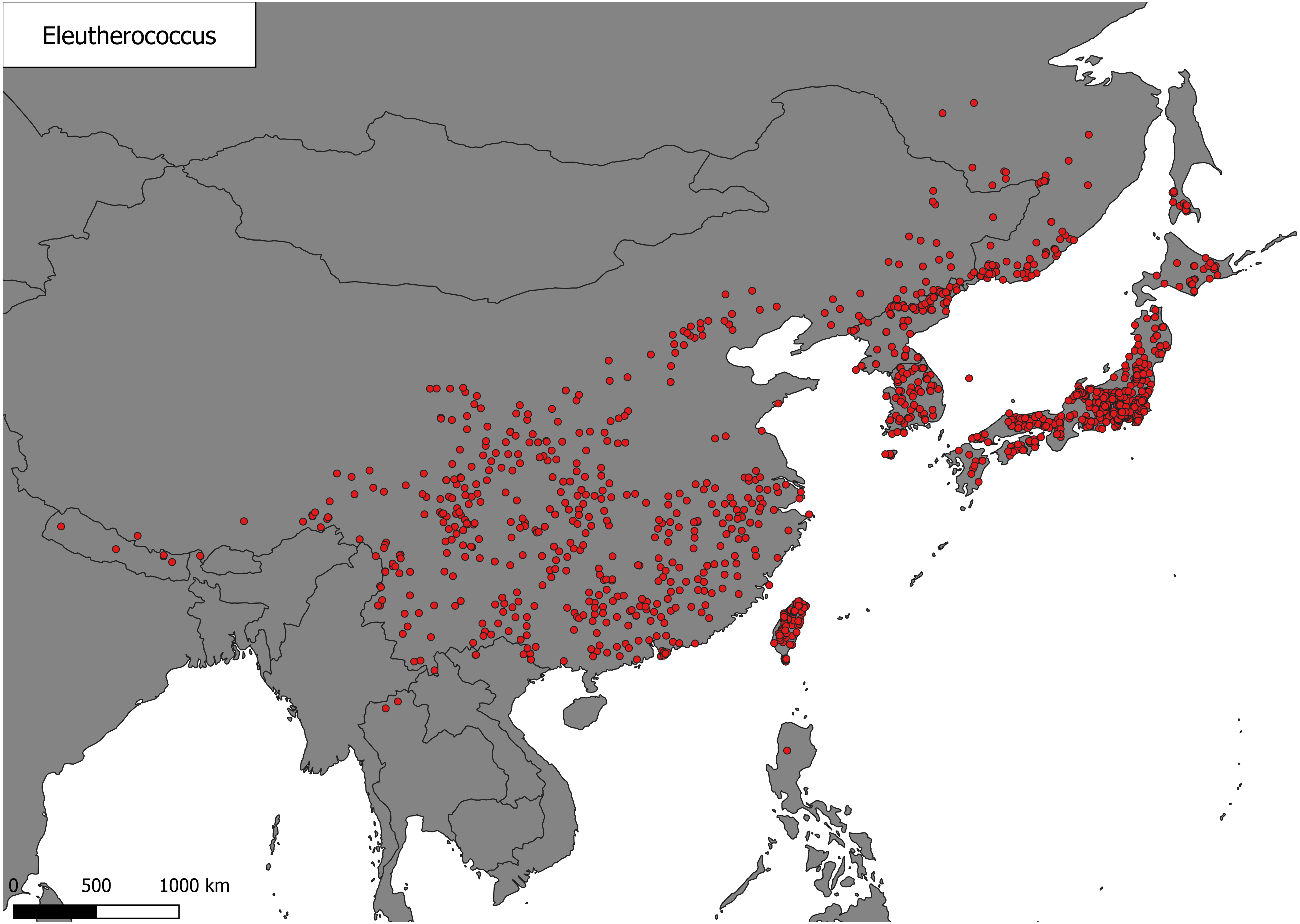
Eleutherococcus
0
500
1000 km

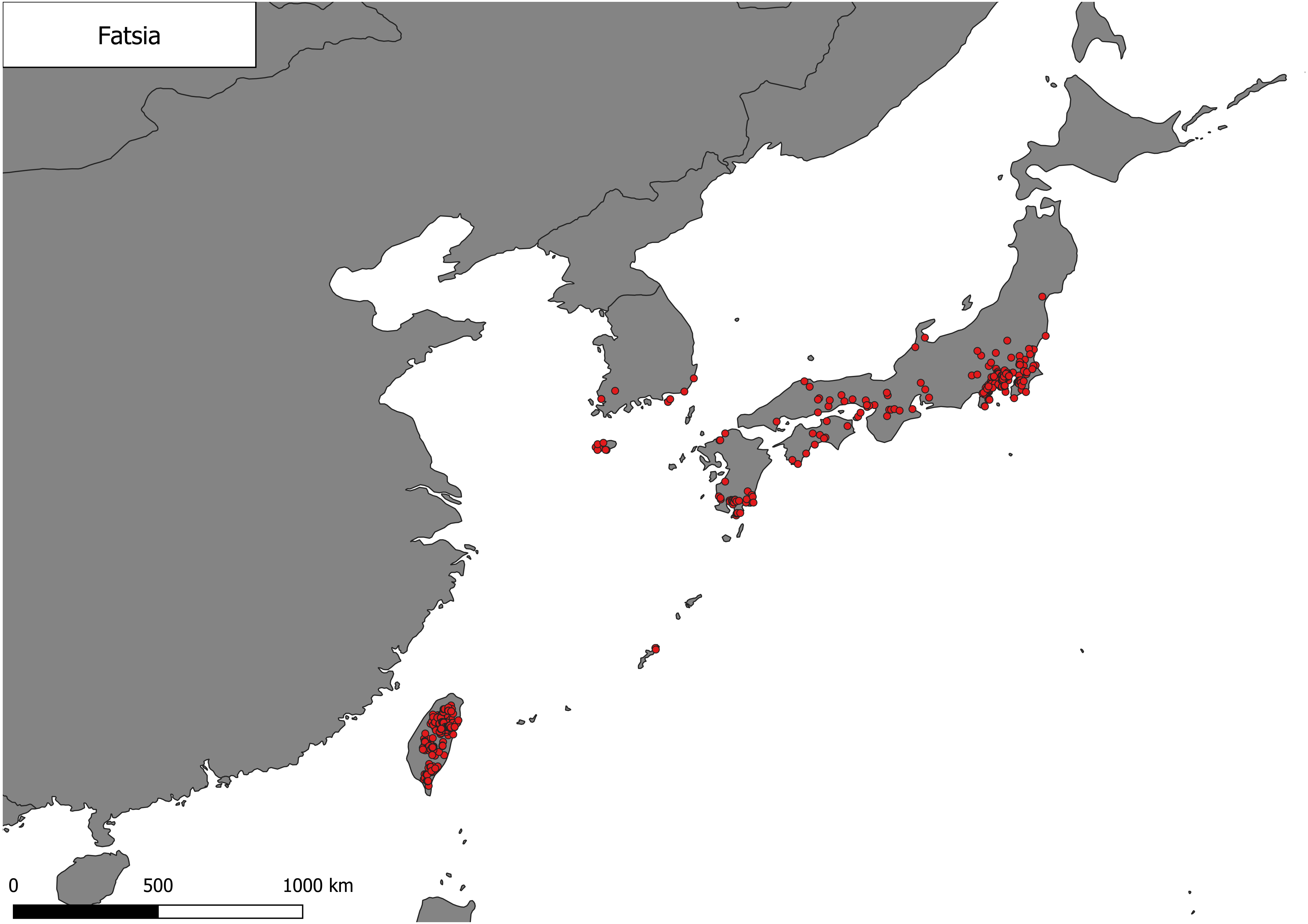
Fatsia
0
500
1000 km

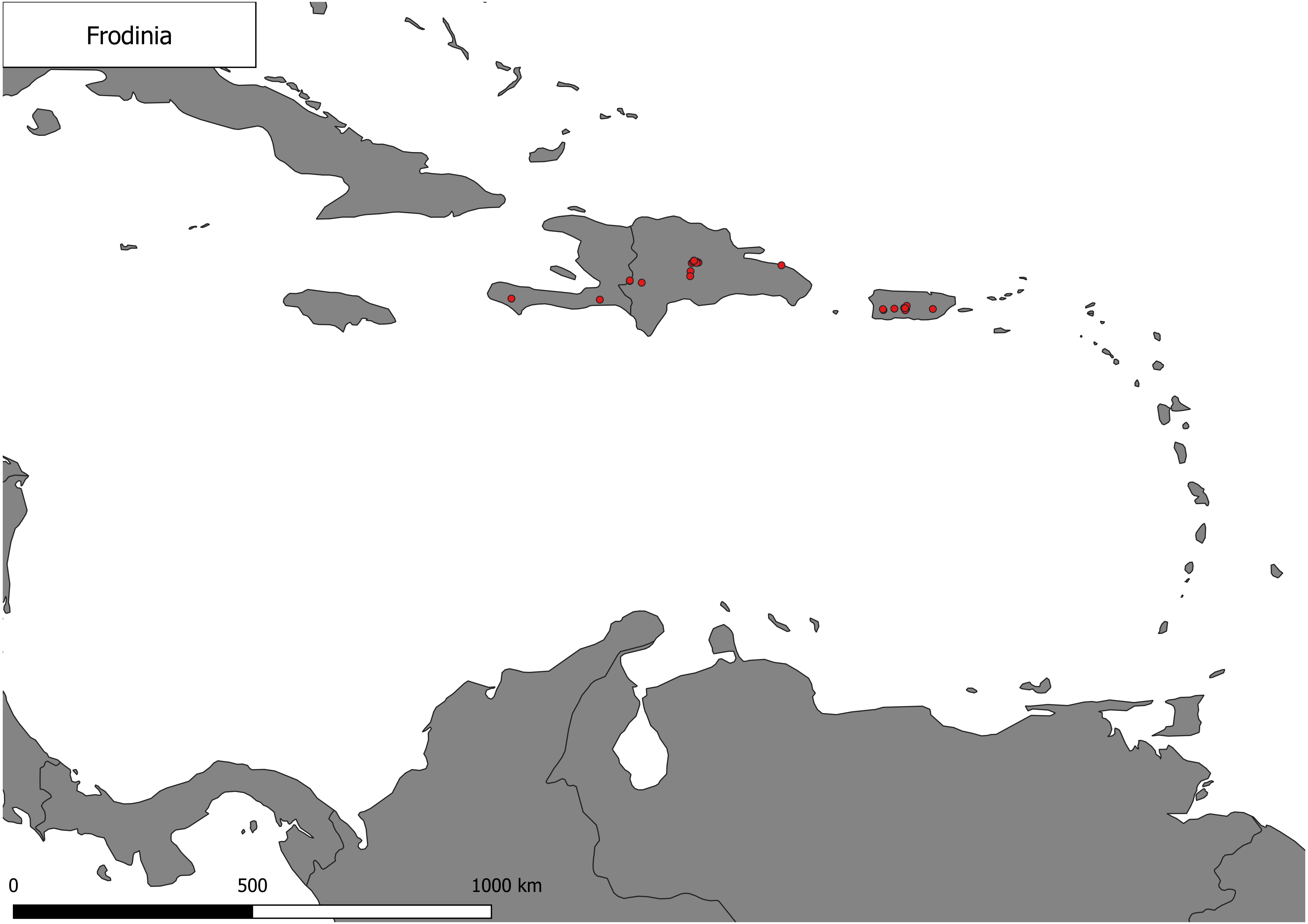

Frodinia
0
500
1000 km

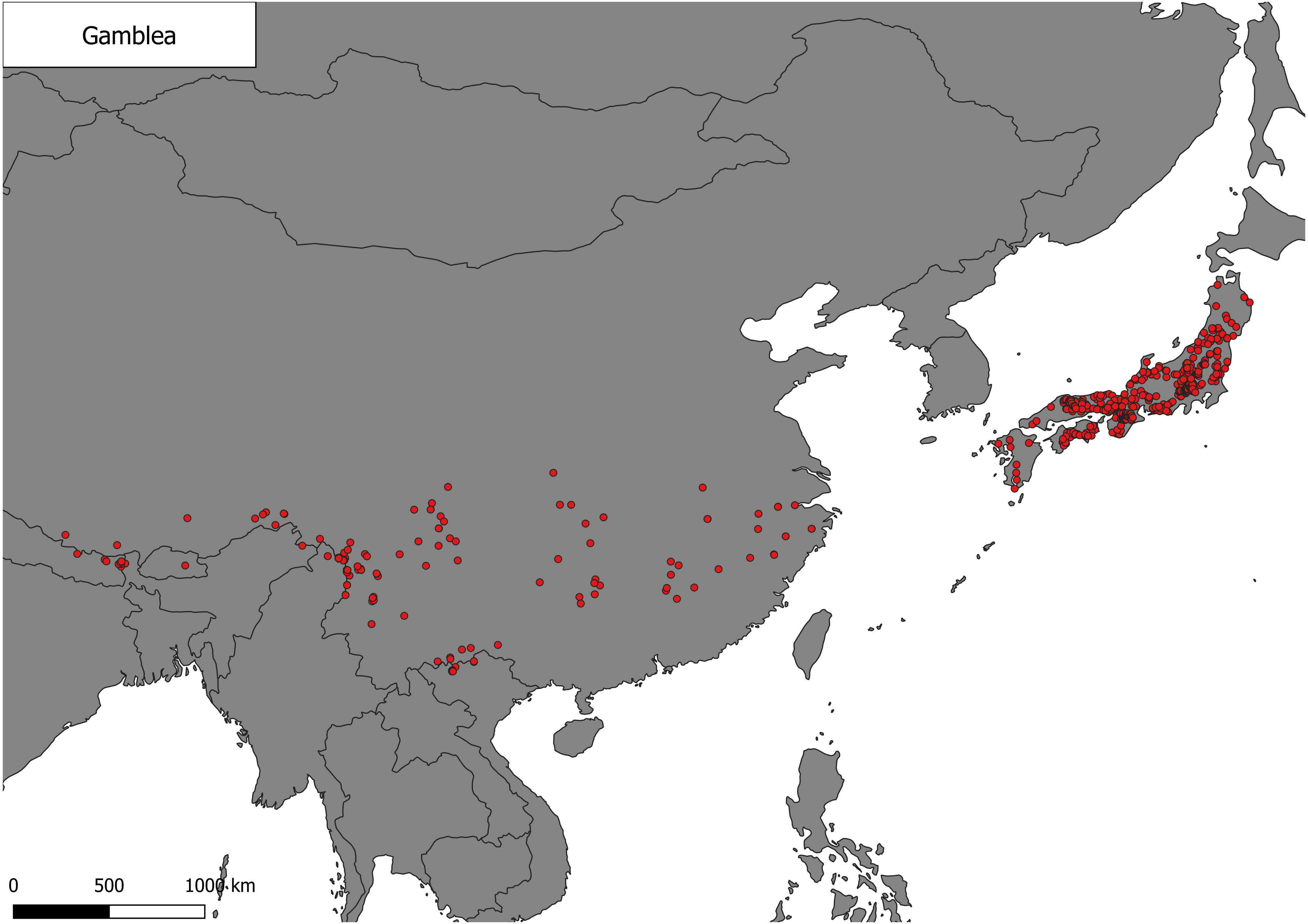
Gamblea
0
500
1000 km

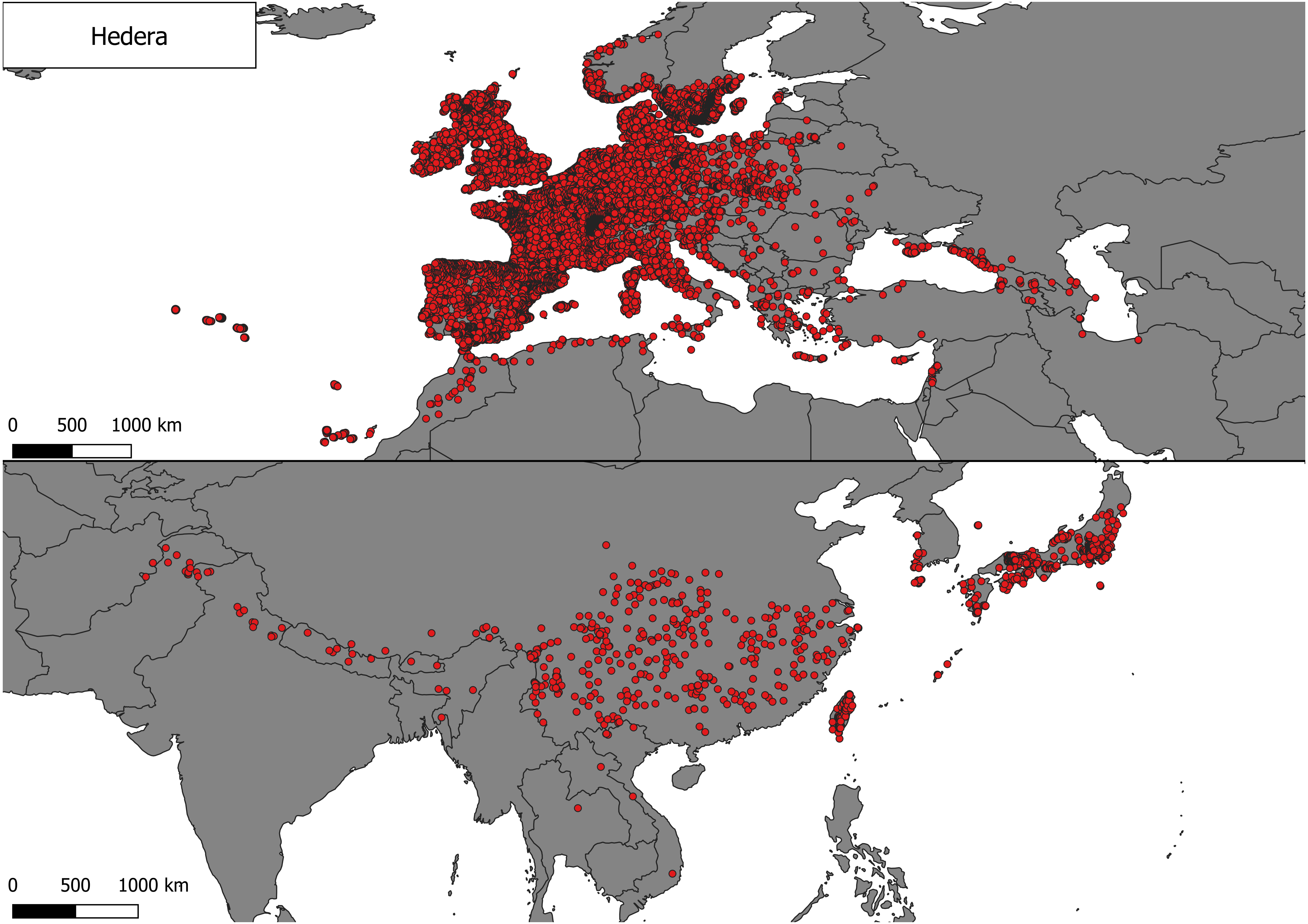

Hedera
0
500
1000 km
0
500
1000 km

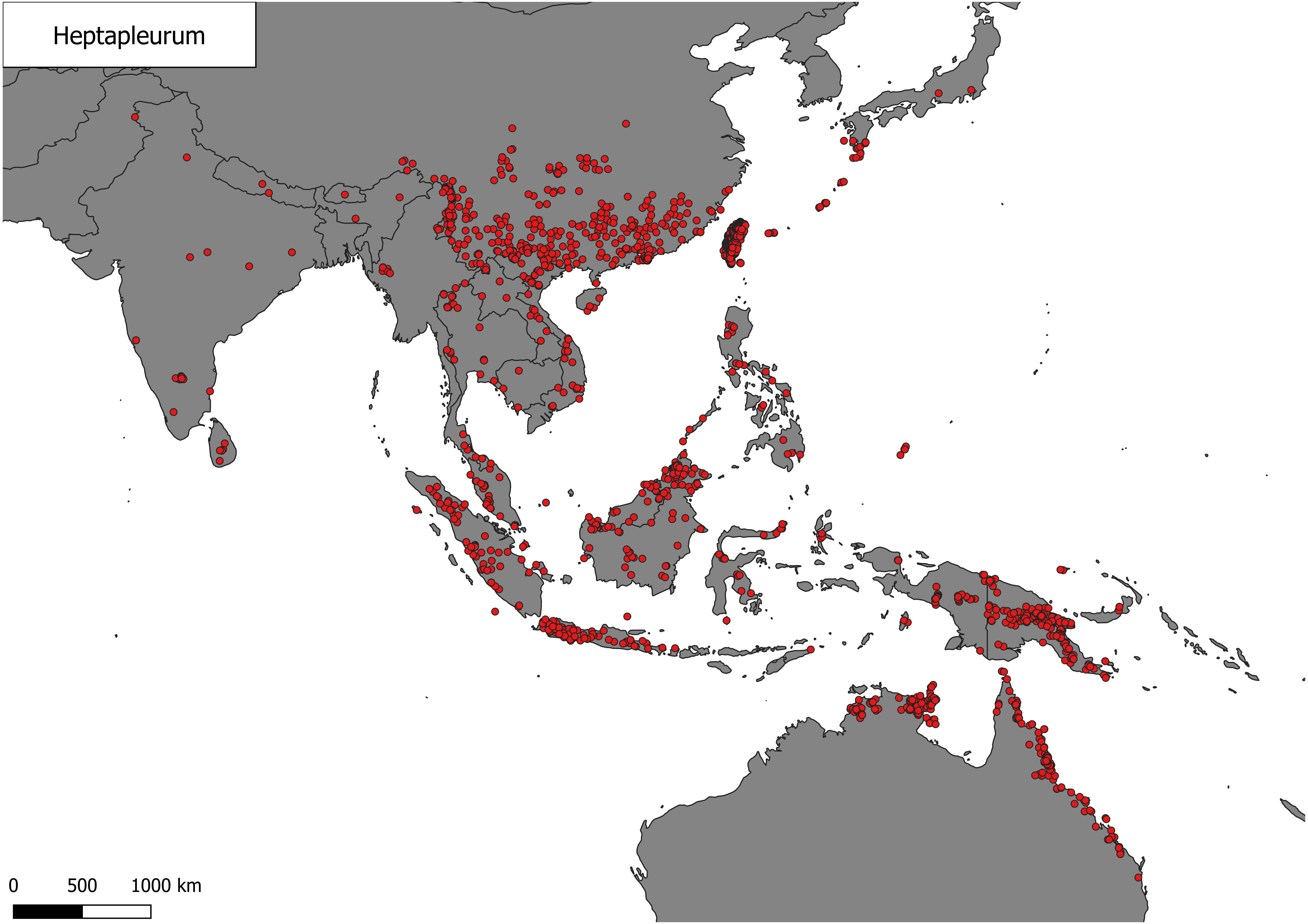
Heptapleurum
0
500
1000 km

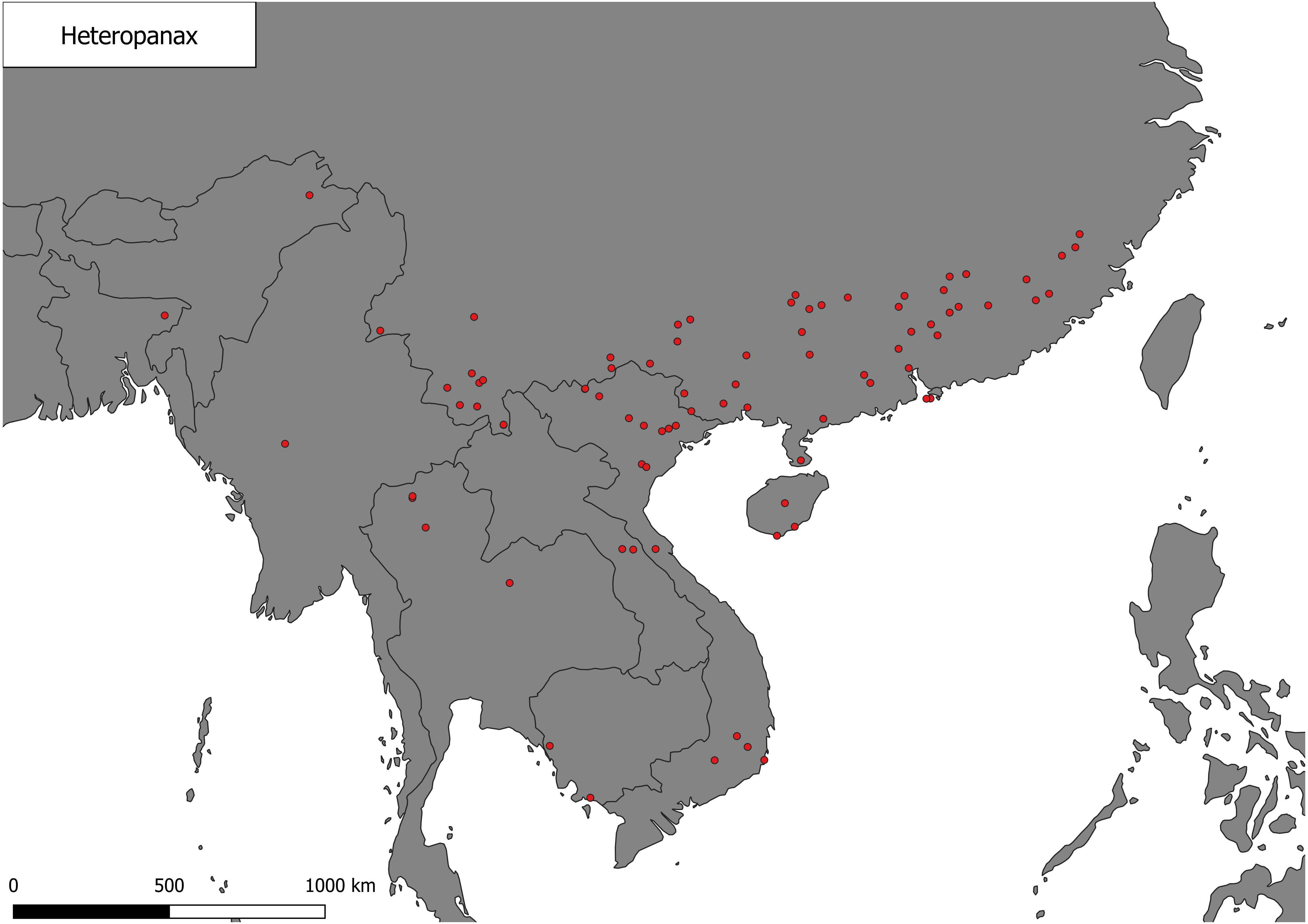
Heteropanax
0
500
1000 km

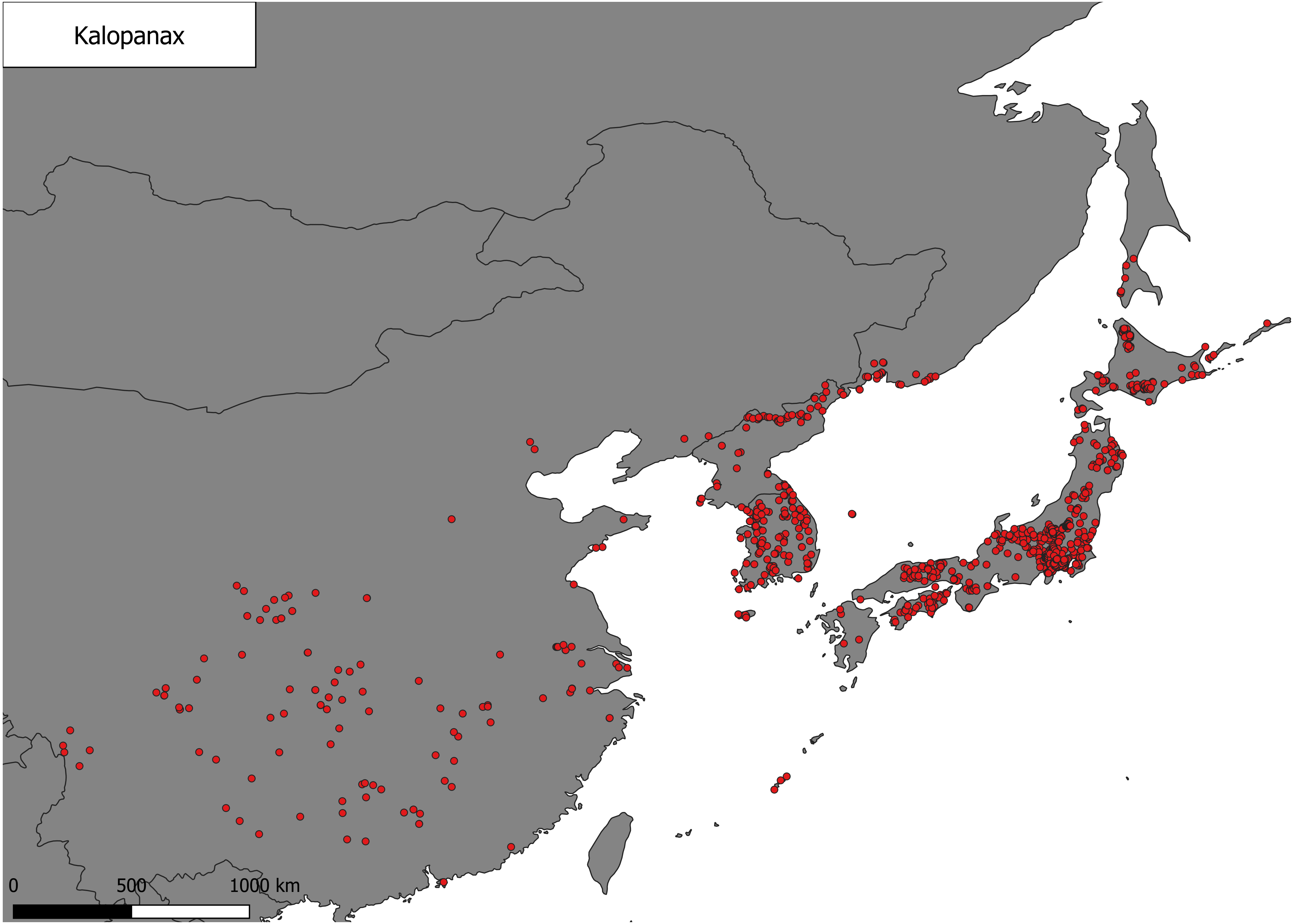
Kalopanax
0
500
1000 km

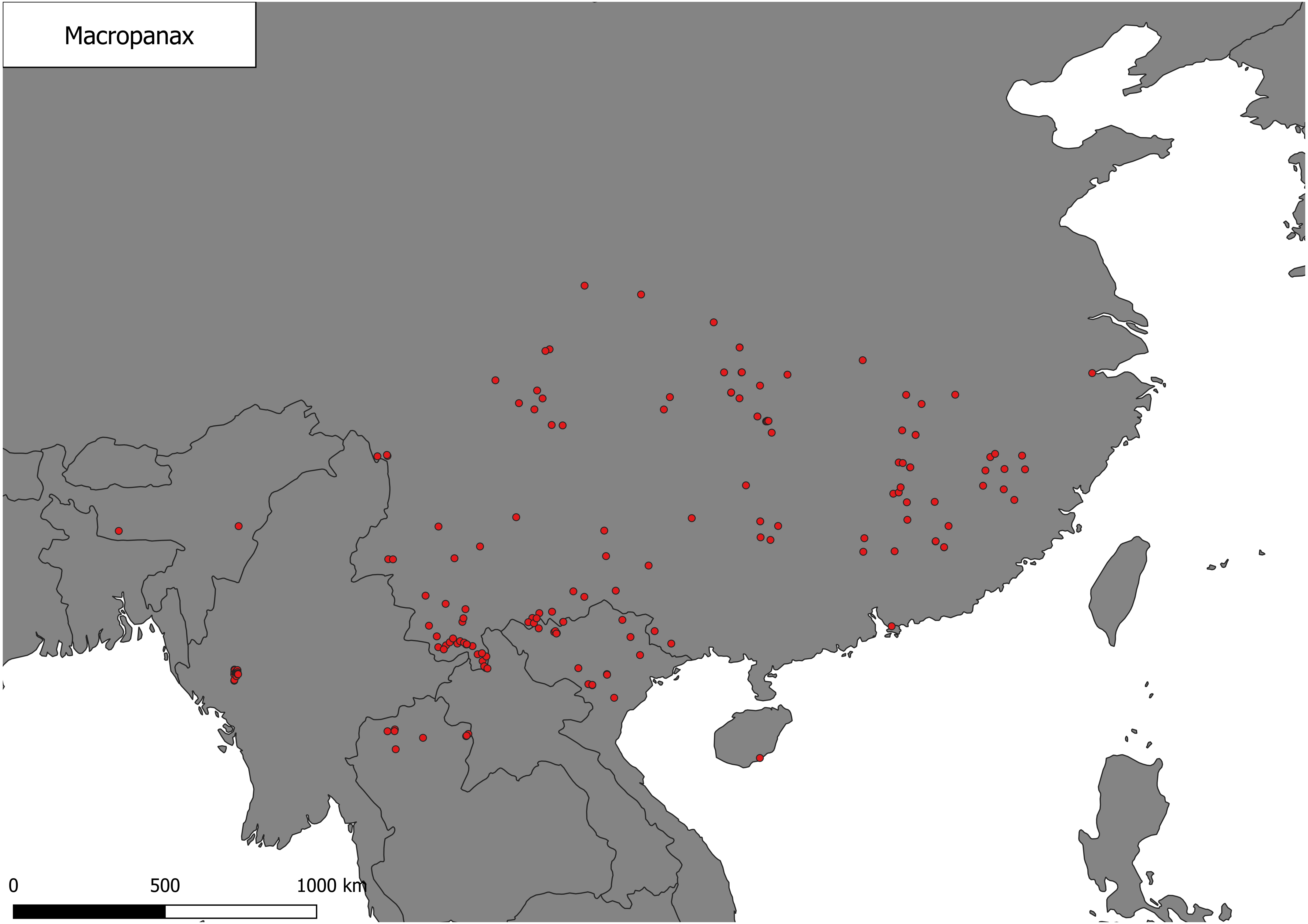
Macropanax
0
500
1000 km

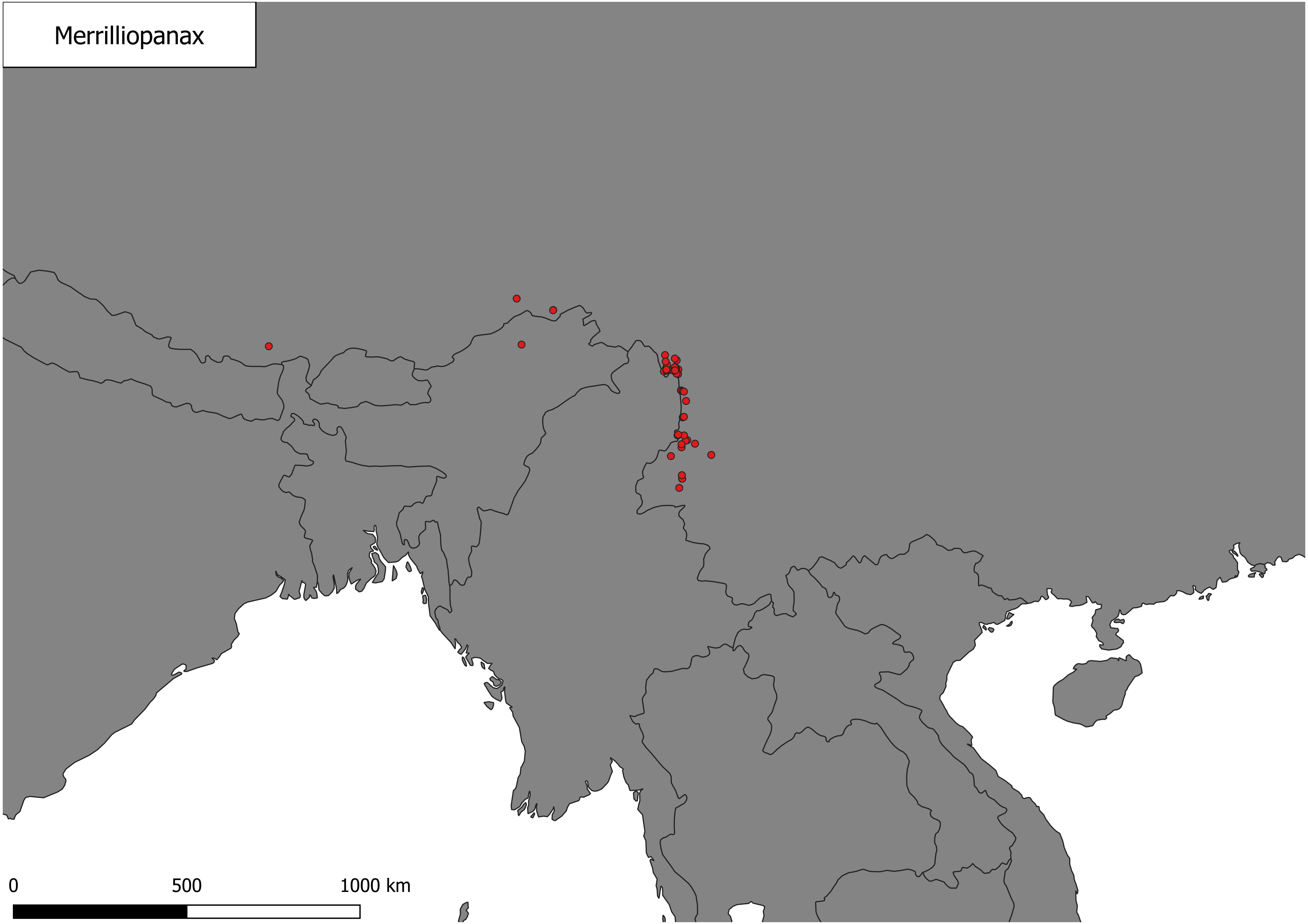
Merrilliopanax
0
500
1000 km

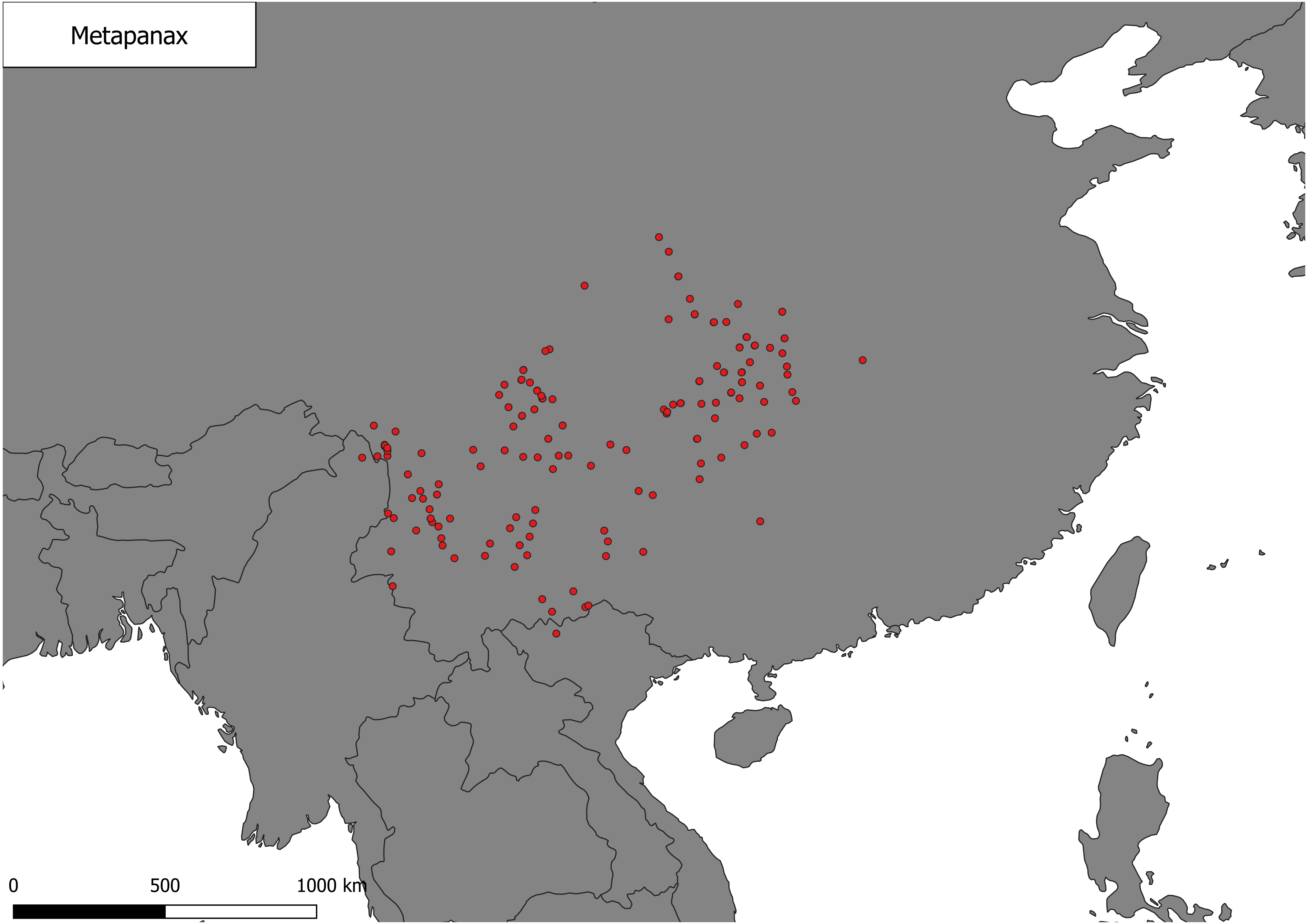
Metapanax
0
500
1000 km

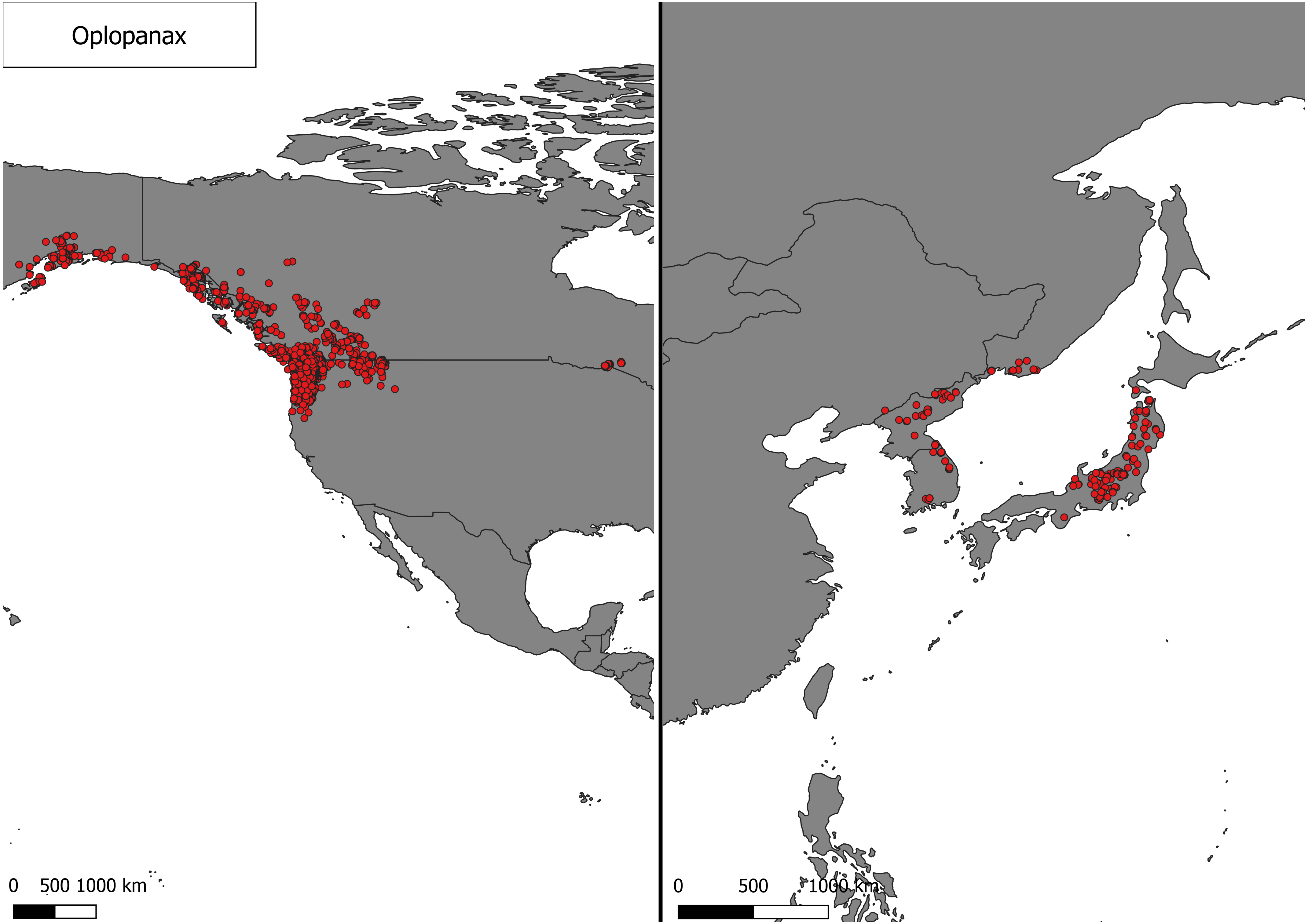
Oplopanax
0 500 1000 km
0 500 1000 km

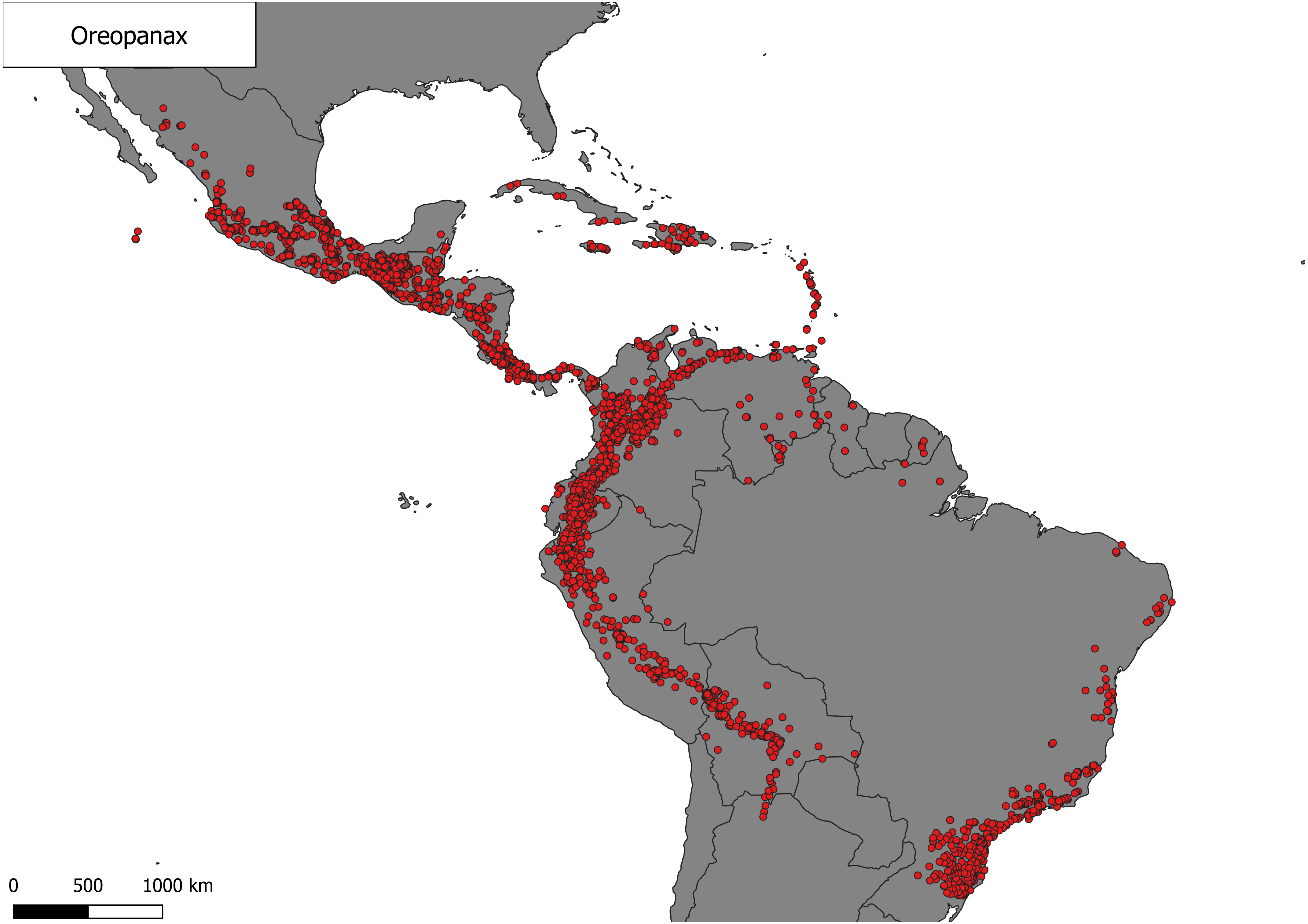
Oreopanax
0
500
1000 km

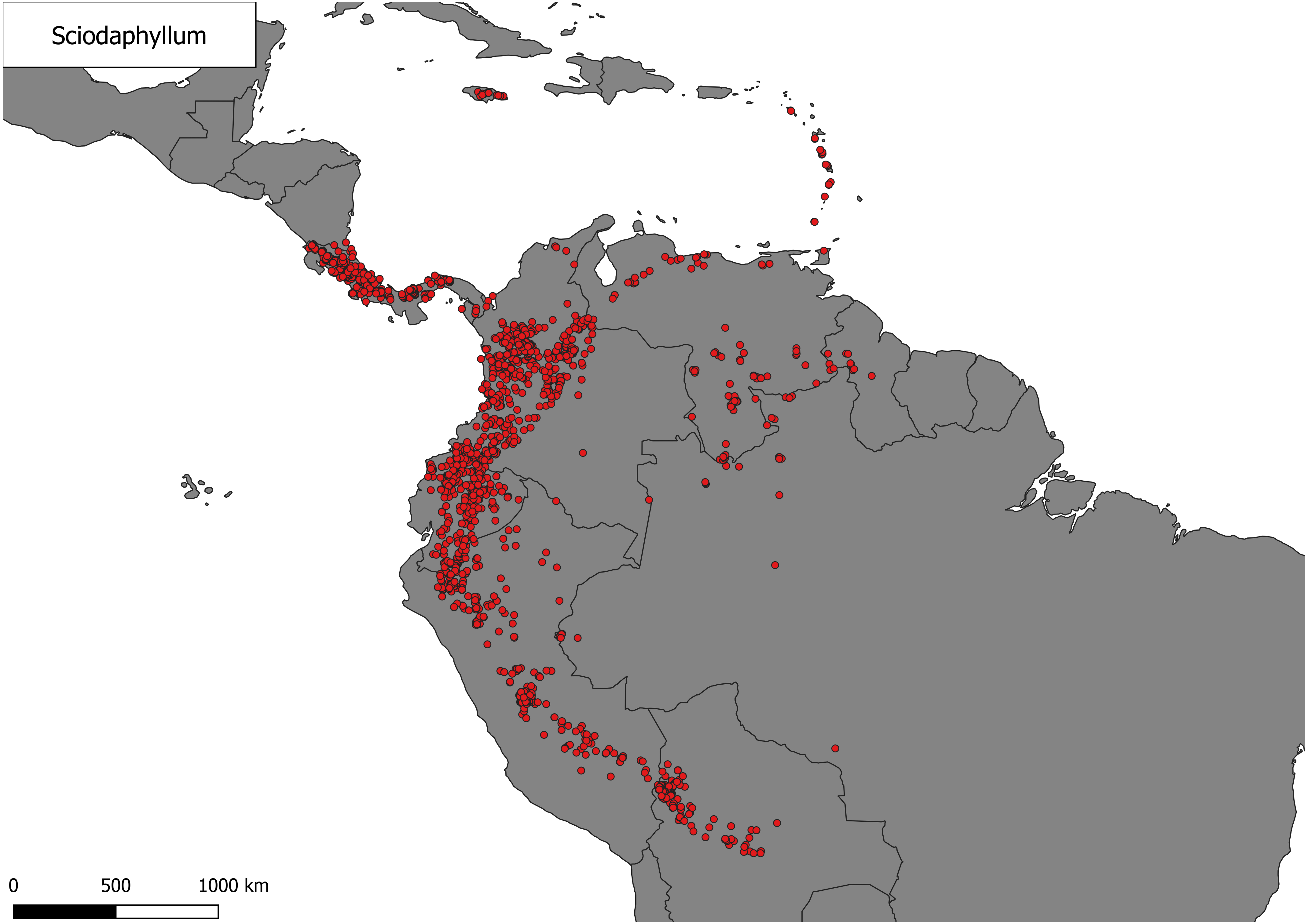
Sciodaphyllum
0
500
1000 km

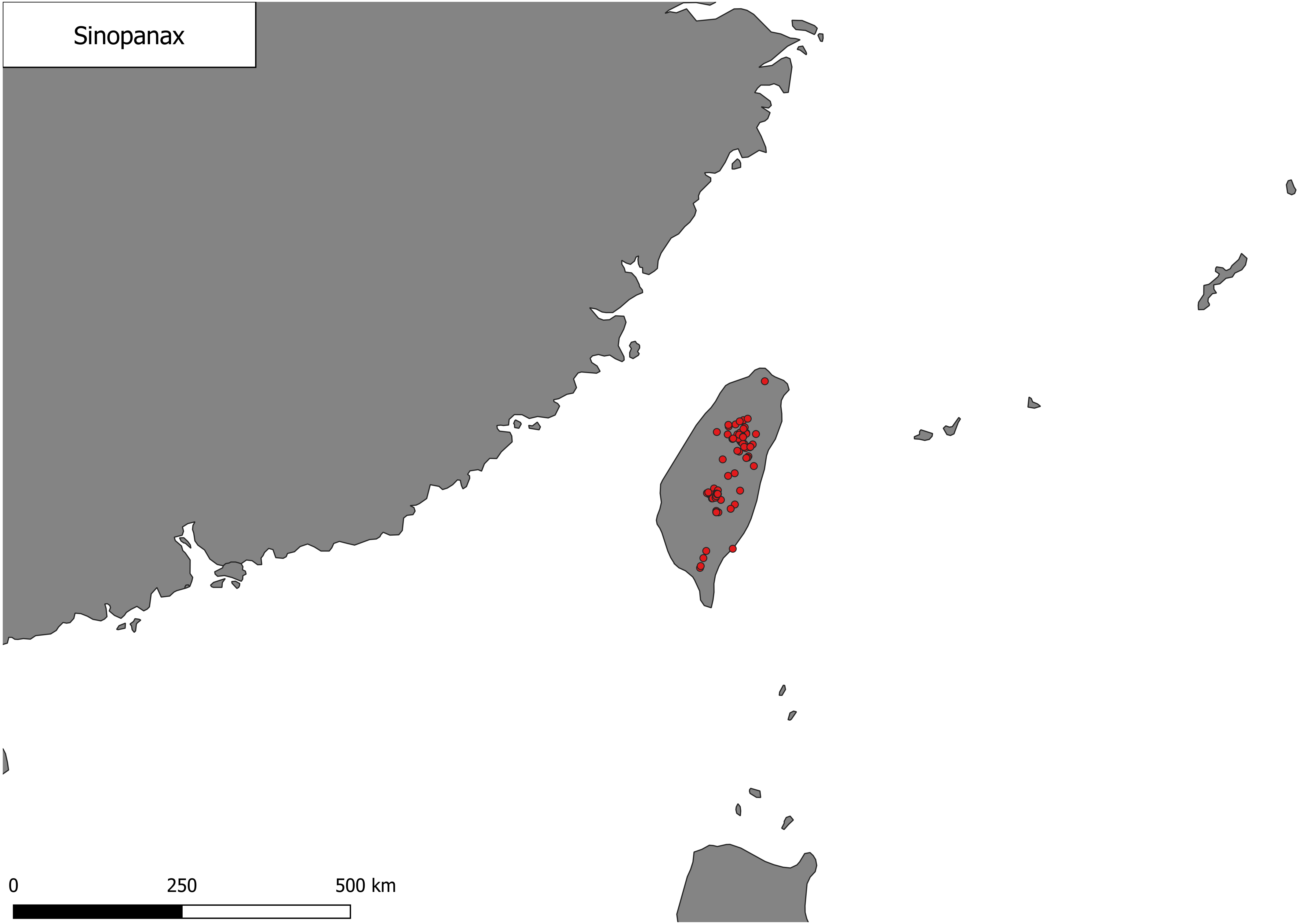

Sinopanax
0
250
500 km

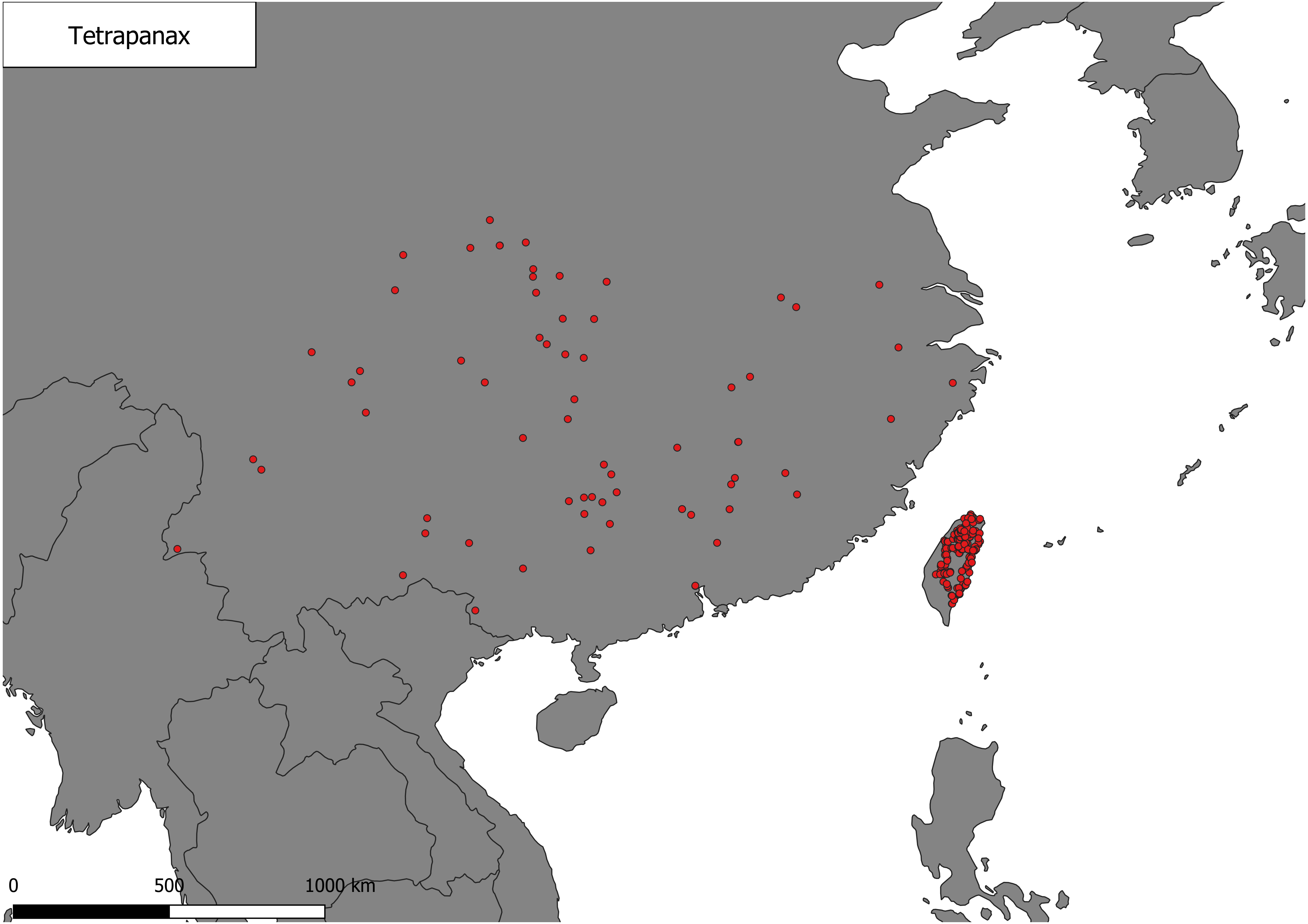
Tetrapanax
0
500
1000 km

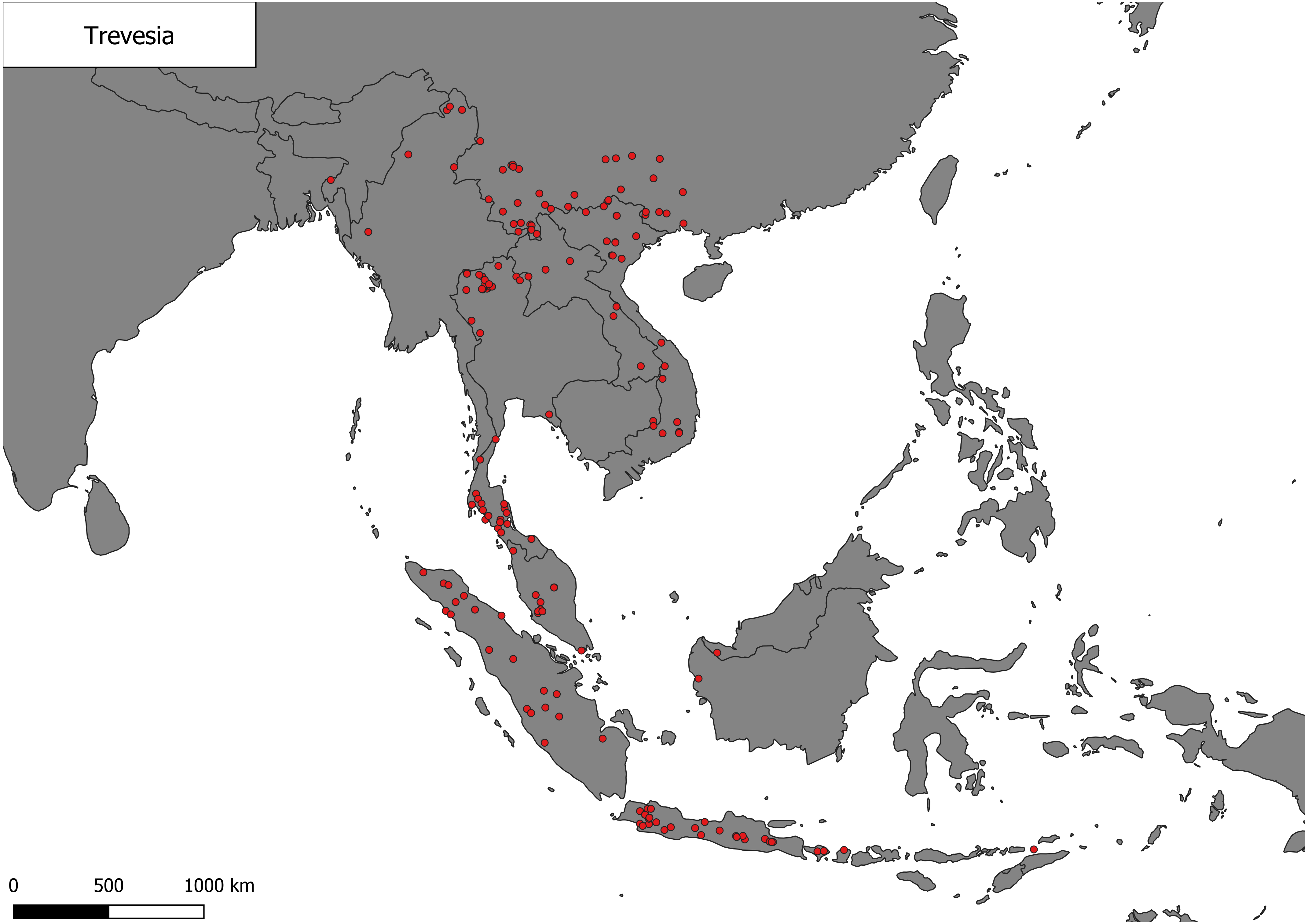
Trevesia
0
500
1000 km
