# Supplementary material for: Evaluation of tropical–temperate transitions: An example of climatic characterization in the Asian Palmate group of Araliaceae
Source: Am J Bot. 2022 Sep 23;109(9):1488–507. doi: 10.1002/ajb2.16059 (PMC9826302; doi:10.1002/ajb2.16059)

Appendix S3. World regionalizations

Appendix S3.1. Latitudinal zonation

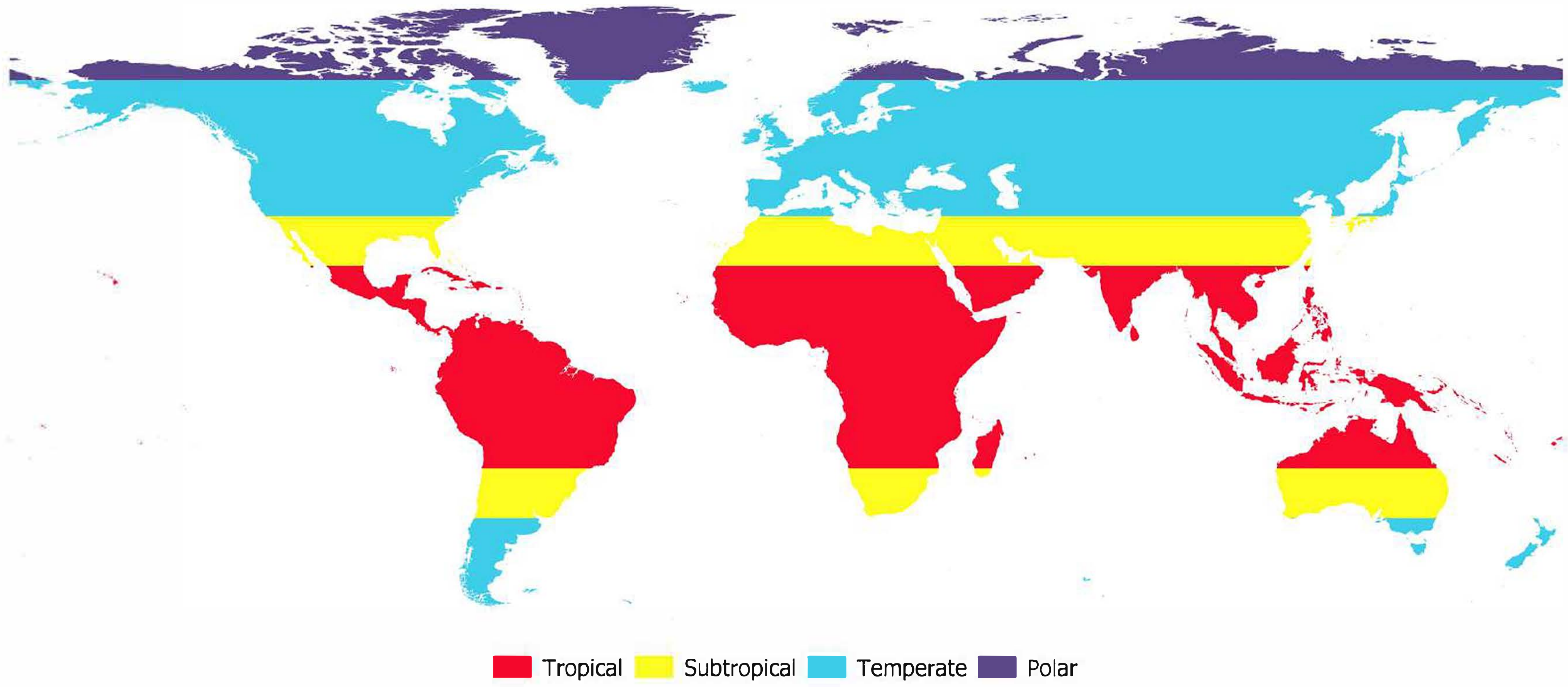

Appendix S3.2. Köppen–Geiger classification (Köppen and Geiger, 1936)

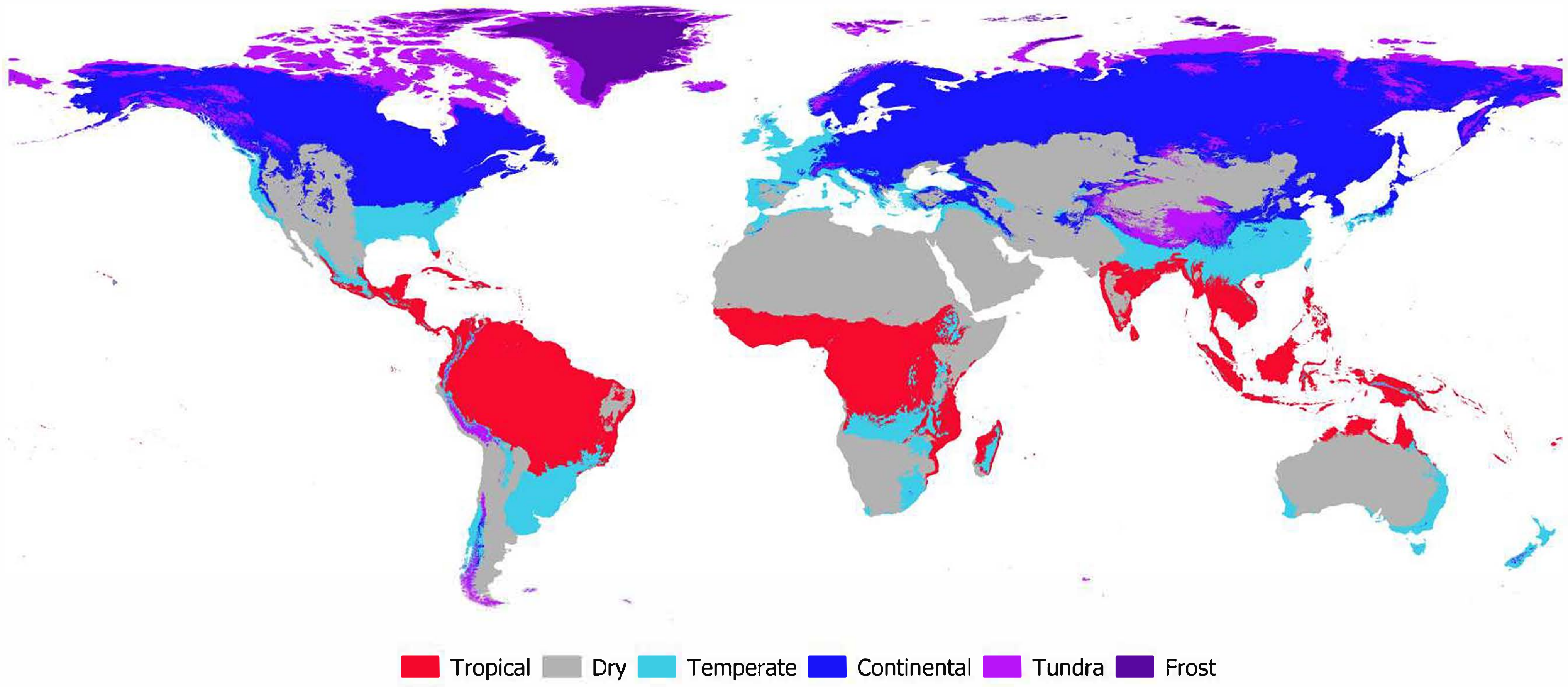

Appendix S2.3. Holdridge classification (Holdridge, 1998)

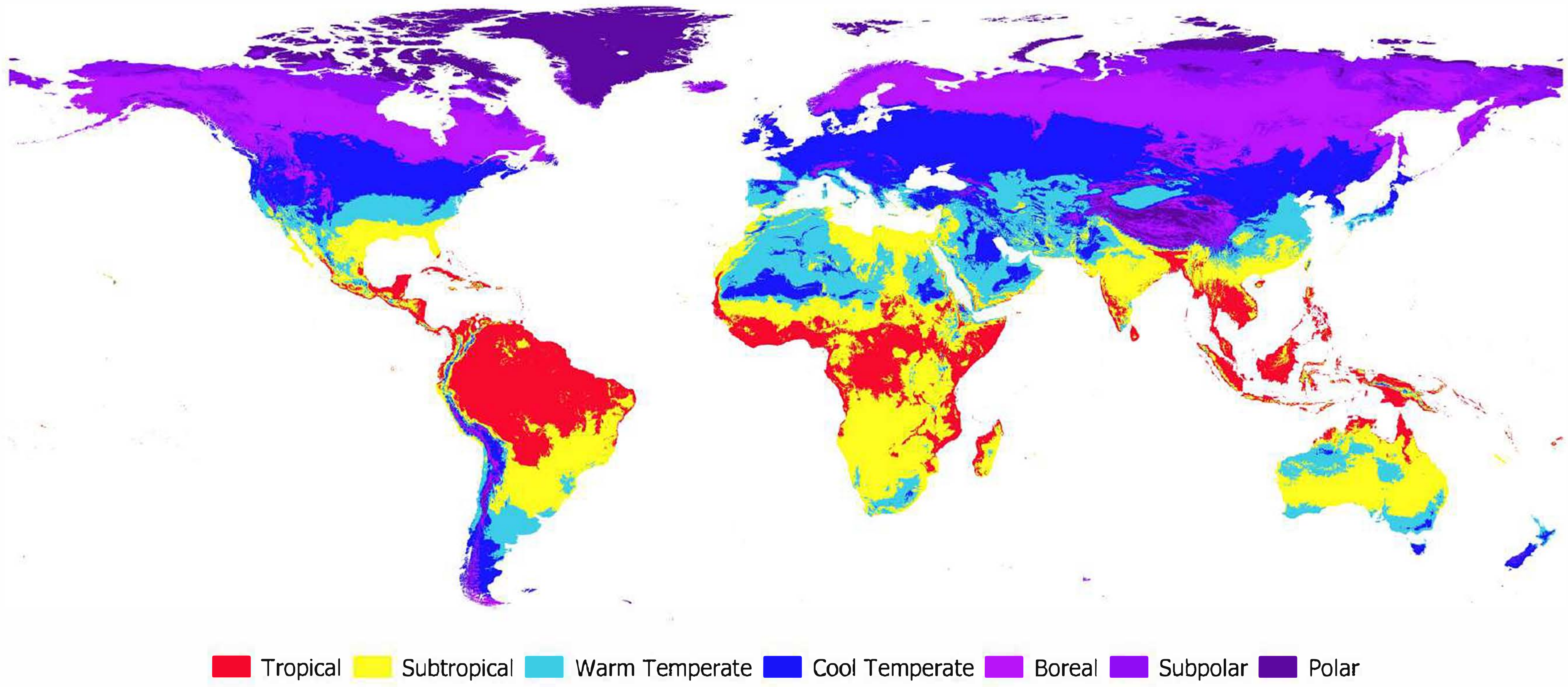

Appendix S3.4. GEnS (Metzger et. al 2012)

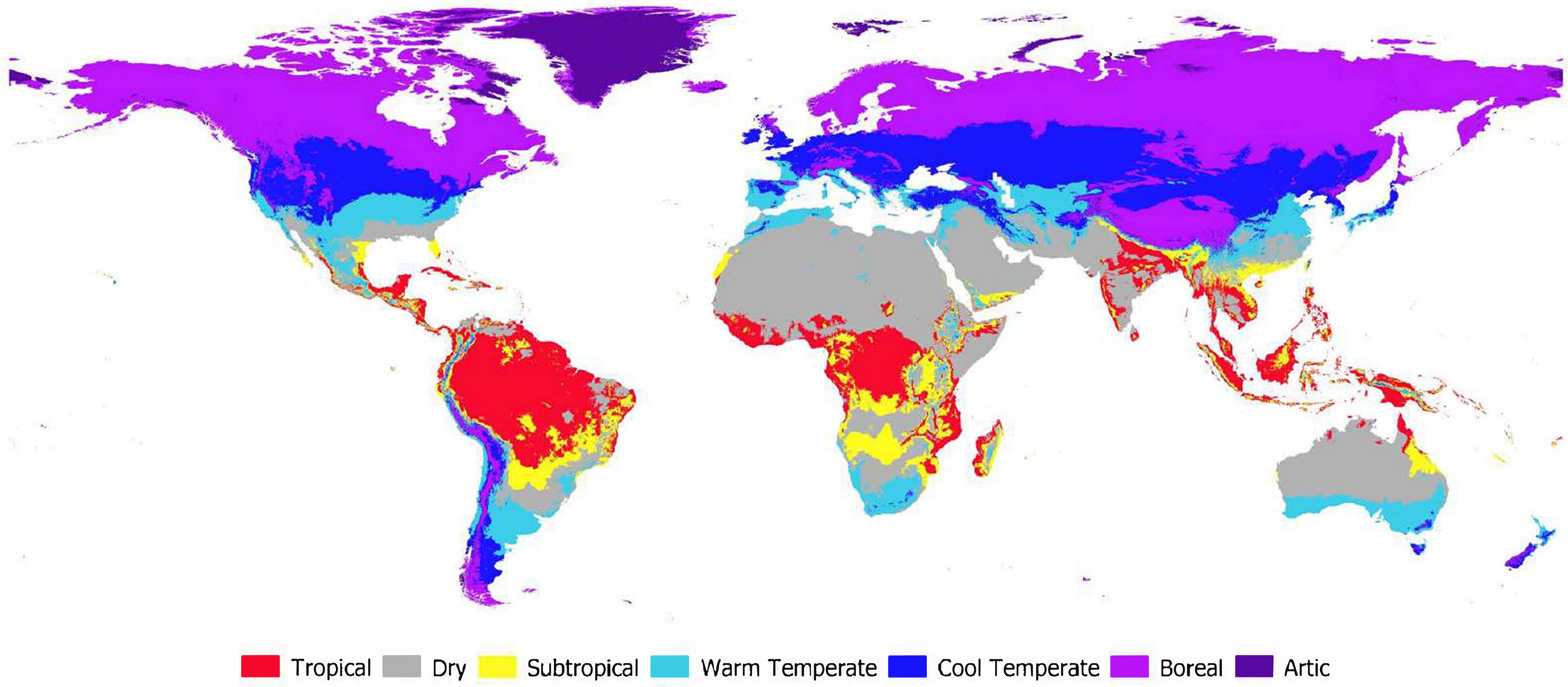

Appendix S3.5. Ecoregions simplified classification (Dinerstein et al., 2017)

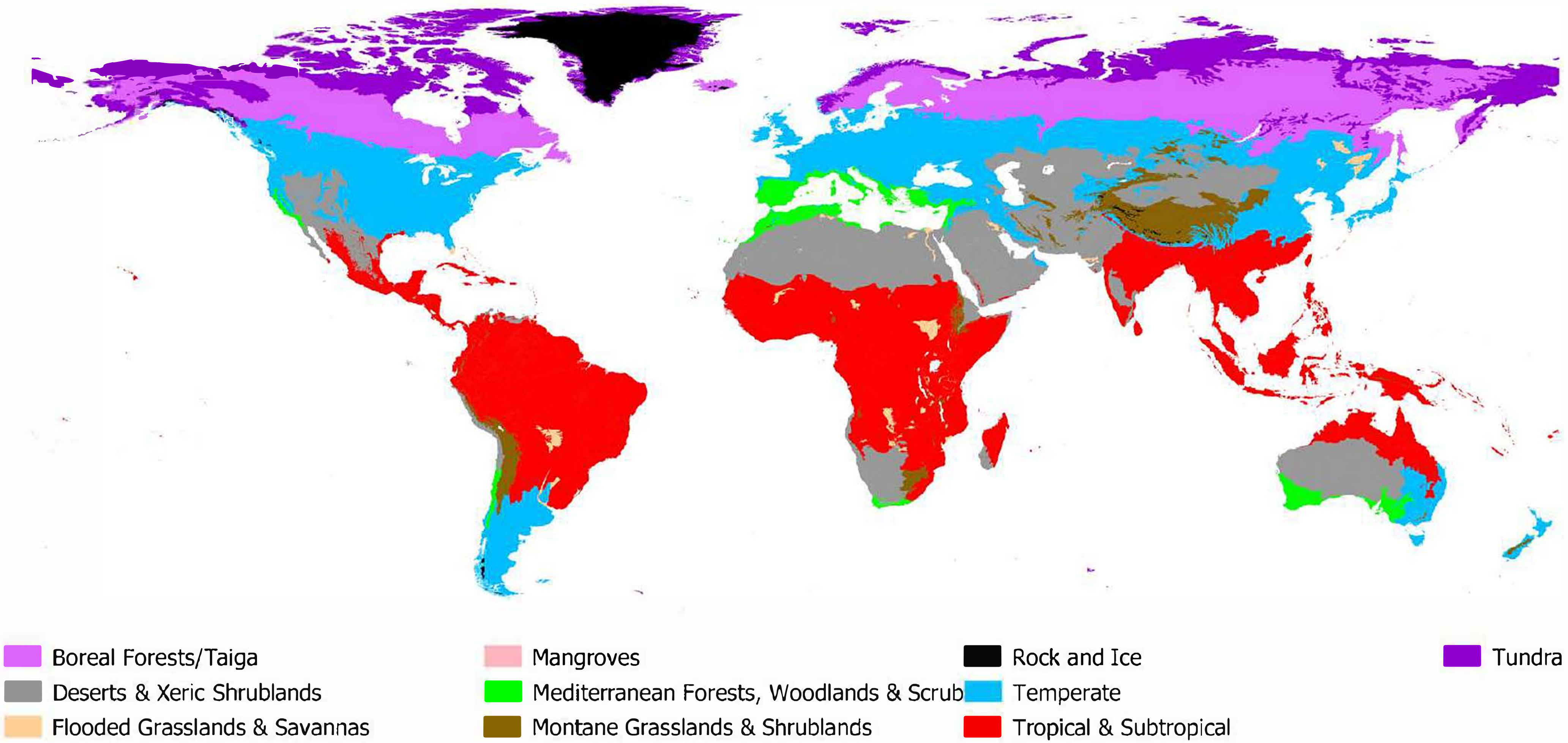

Supplement: Supplementary file 3 — Appendix S3. World regionalizations. [file AJB2-109-1488-s002.pdf]
