# Supplementary material for: Evaluation of tropical–temperate transitions: An example of climatic characterization in the Asian Palmate group of Araliaceae
Source: Am J Bot. 2022 Sep 23;109(9):1488–507. doi: 10.1002/ajb2.16059 (PMC9826302; doi:10.1002/ajb2.16059)
Supplement: Supplementary file 1 — Appendix S1. Heat maps of Asian Palmate Group genera. [file AJB2-109-1488-s003.pdf]

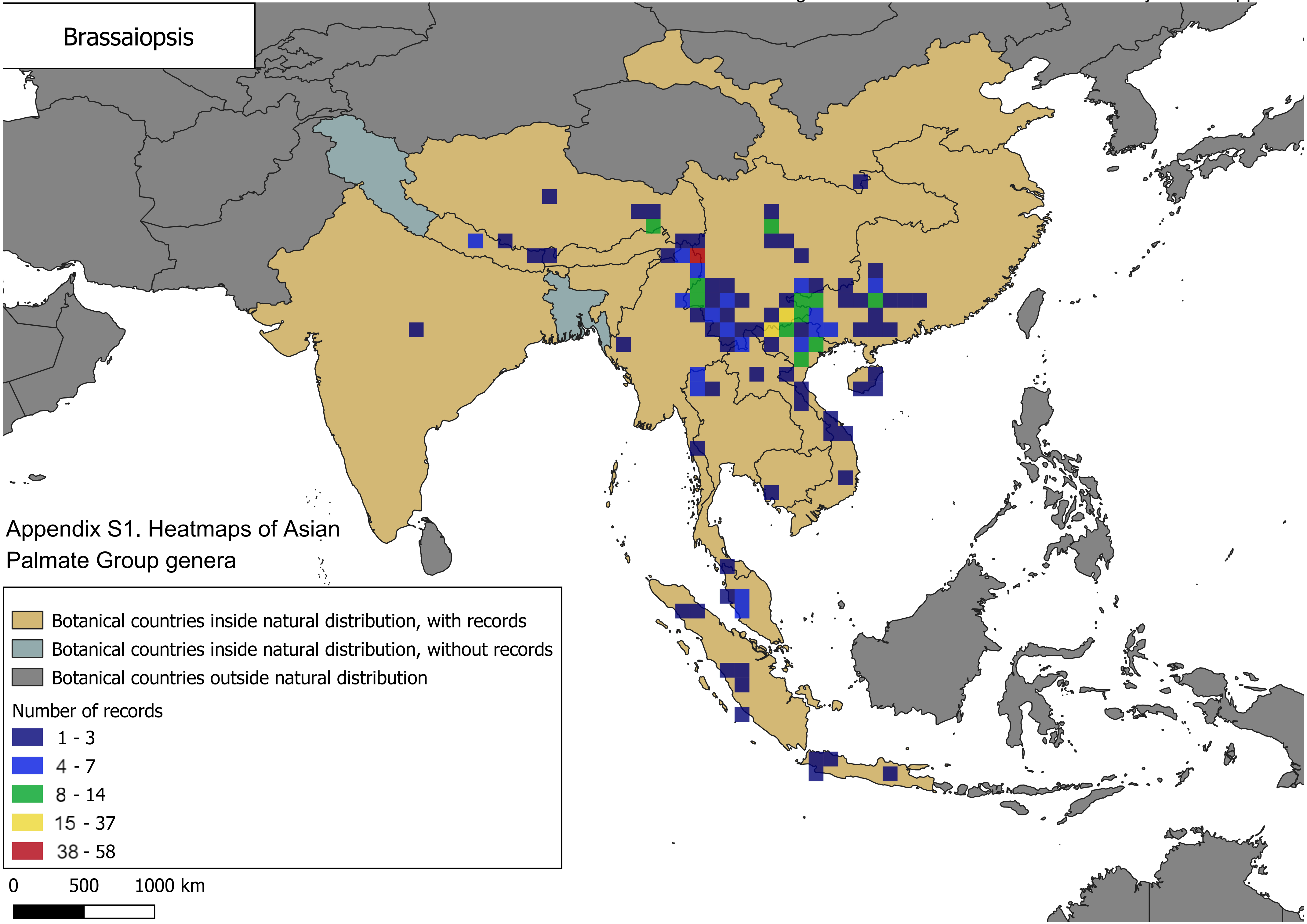
Brassaiopsis
Appendix S1. Heatmaps of Asian Palmate Group genera
Botanical countries inside natural distribution, with records
Botanical countries inside natural distribution, without records
Botanical countries outside natural distribution
Number of records
1 - 3
4 - 7
8 - 14
15 - 37
38 - 58
0
500
1000 km

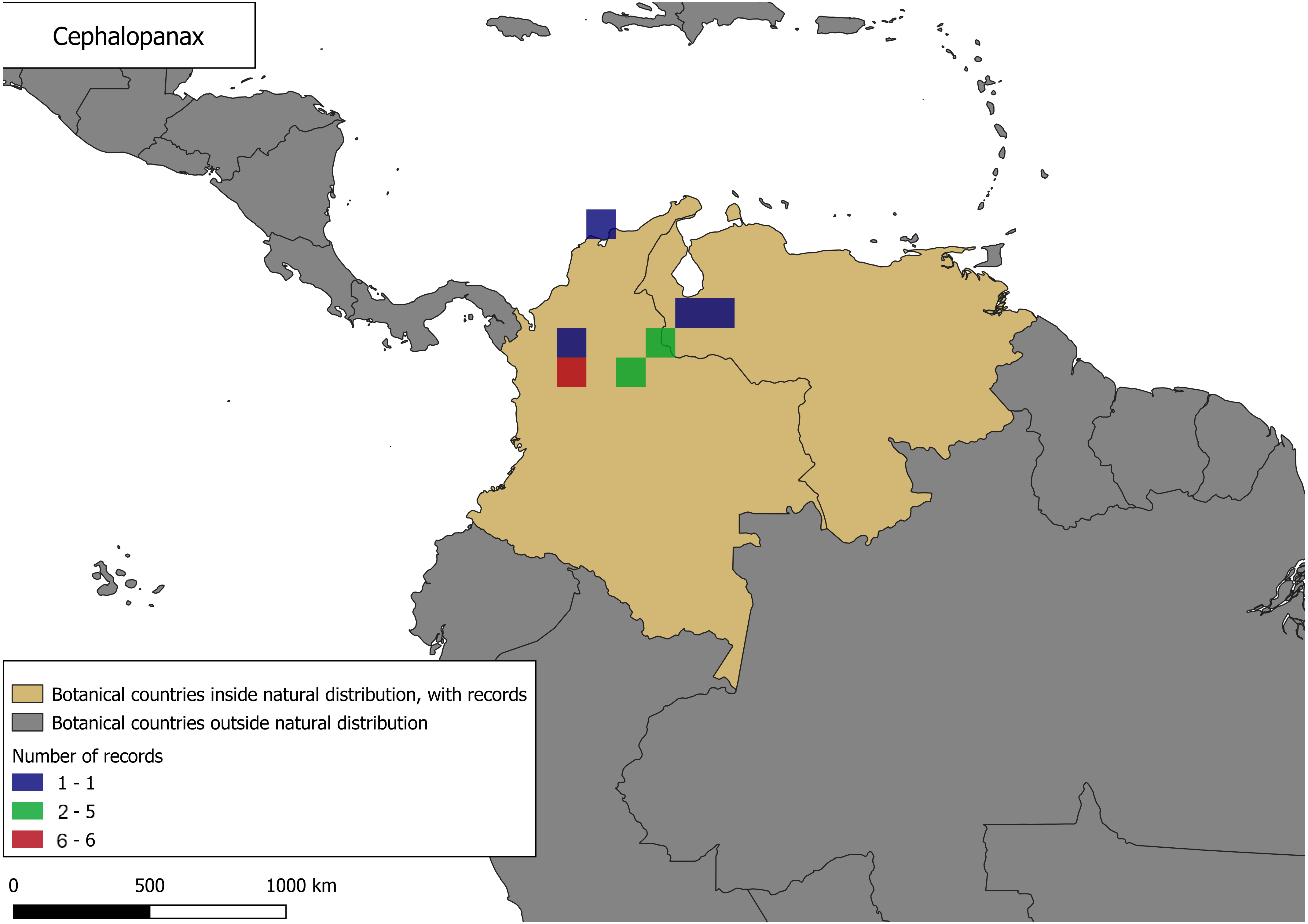

Cephalopanax
Botanical countries inside natural distribution, with records
Botanical countries outside natural distribution
Number of records
1 - 1
2 - 5
6 - 6
0
500
1000 km

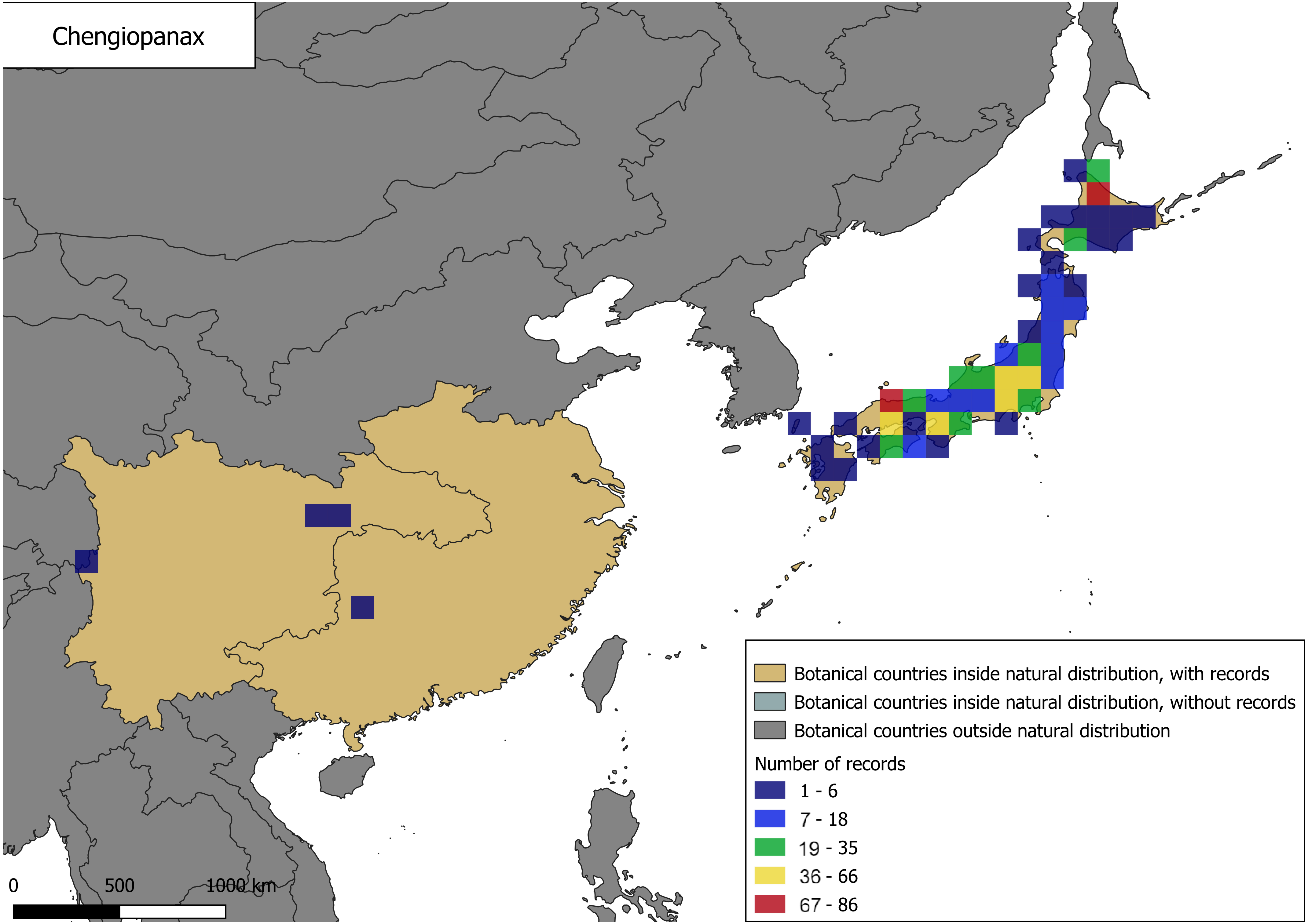
Chengiopanax
Botanical countries inside natural distribution, with records
Botanical countries inside natural distribution, without records
Botanical countries outside natural distribution
Number of records
1 - 6
7 - 18
19 - 35
36 - 66
67 - 86
0
500
1000 km

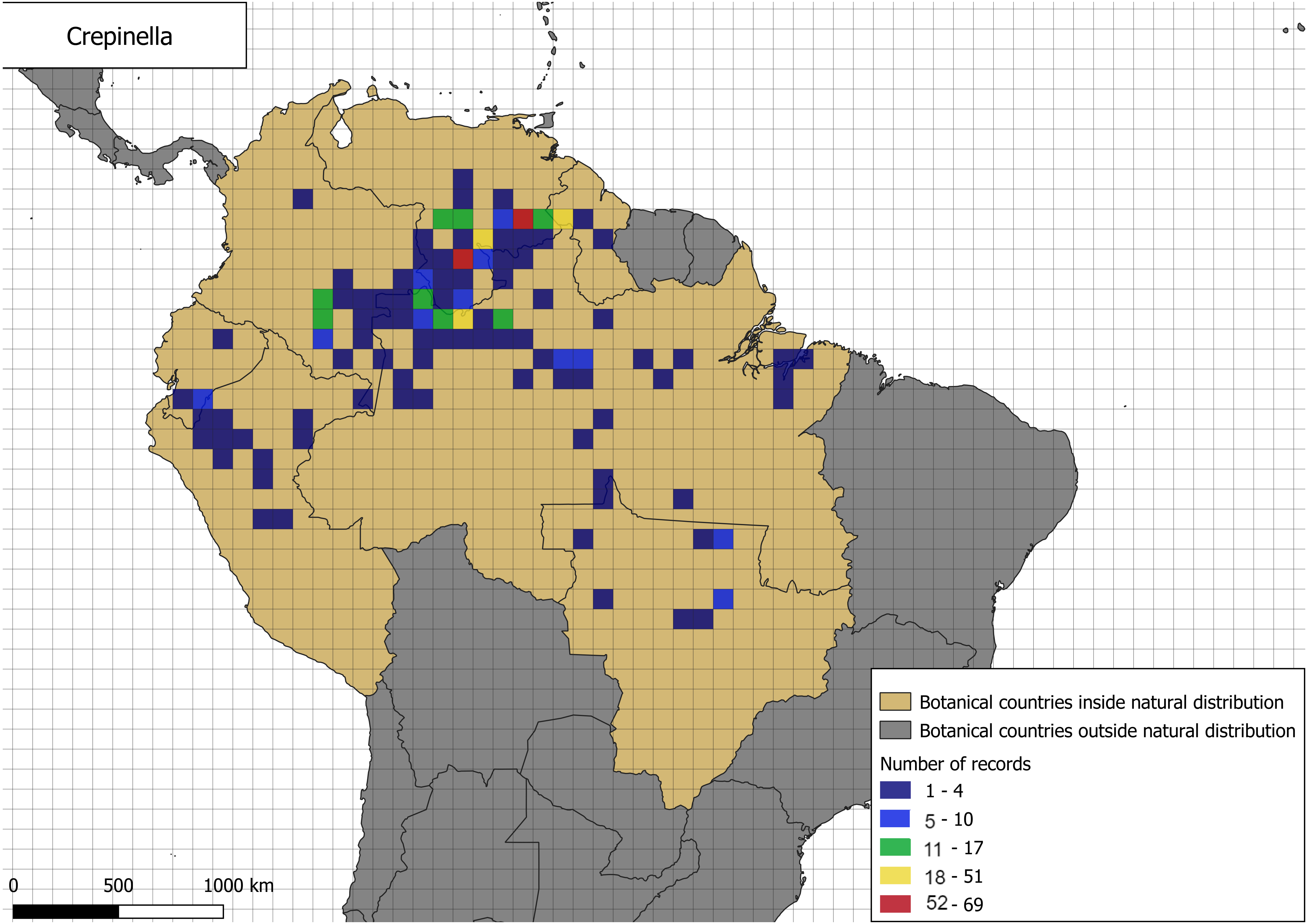
Crepinella
Botanical countries inside natural distribution
Botanical countries outside natural distribution
Number of records
1 - 4
5 - 10
11 - 17
18 - 51
52 - 69
0
500
1000 km

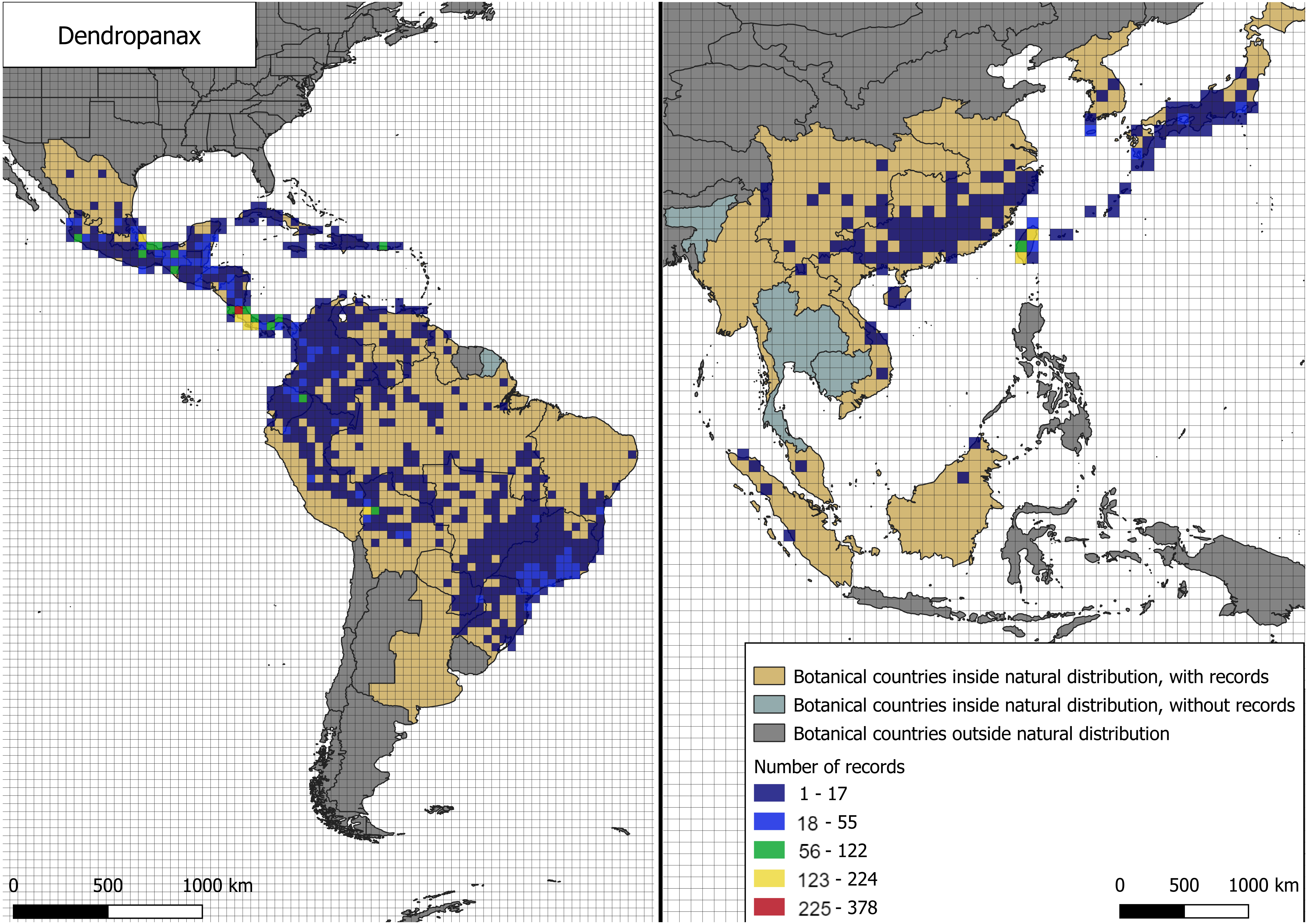

Dendropanax
Botanical countries inside natural distribution, with records
Botanical countries inside natural distribution, without records
Botanical countries outside natural distribution
Number of records
1 - 17
18 - 55
56 - 122
123 - 224
225 - 378
0
500
1000 km
0
500
1000 km

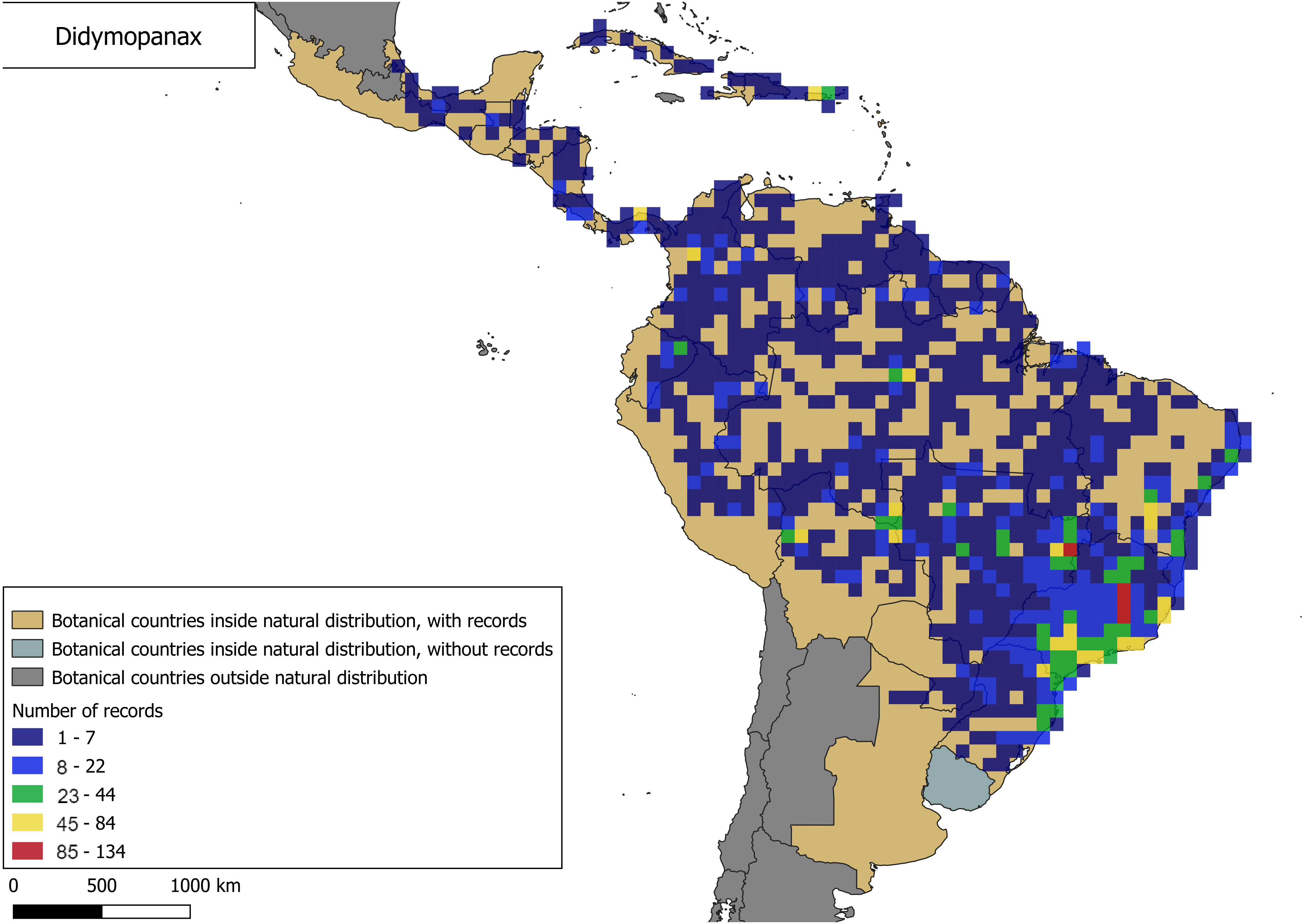
Didymopanax
Botanical countries inside natural distribution, with records
Botanical countries inside natural distribution, without records
Botanical countries outside natural distribution
Number of records
1 - 7
8 - 22
23 - 44
45 - 84
85 - 134
0
500
1000 km

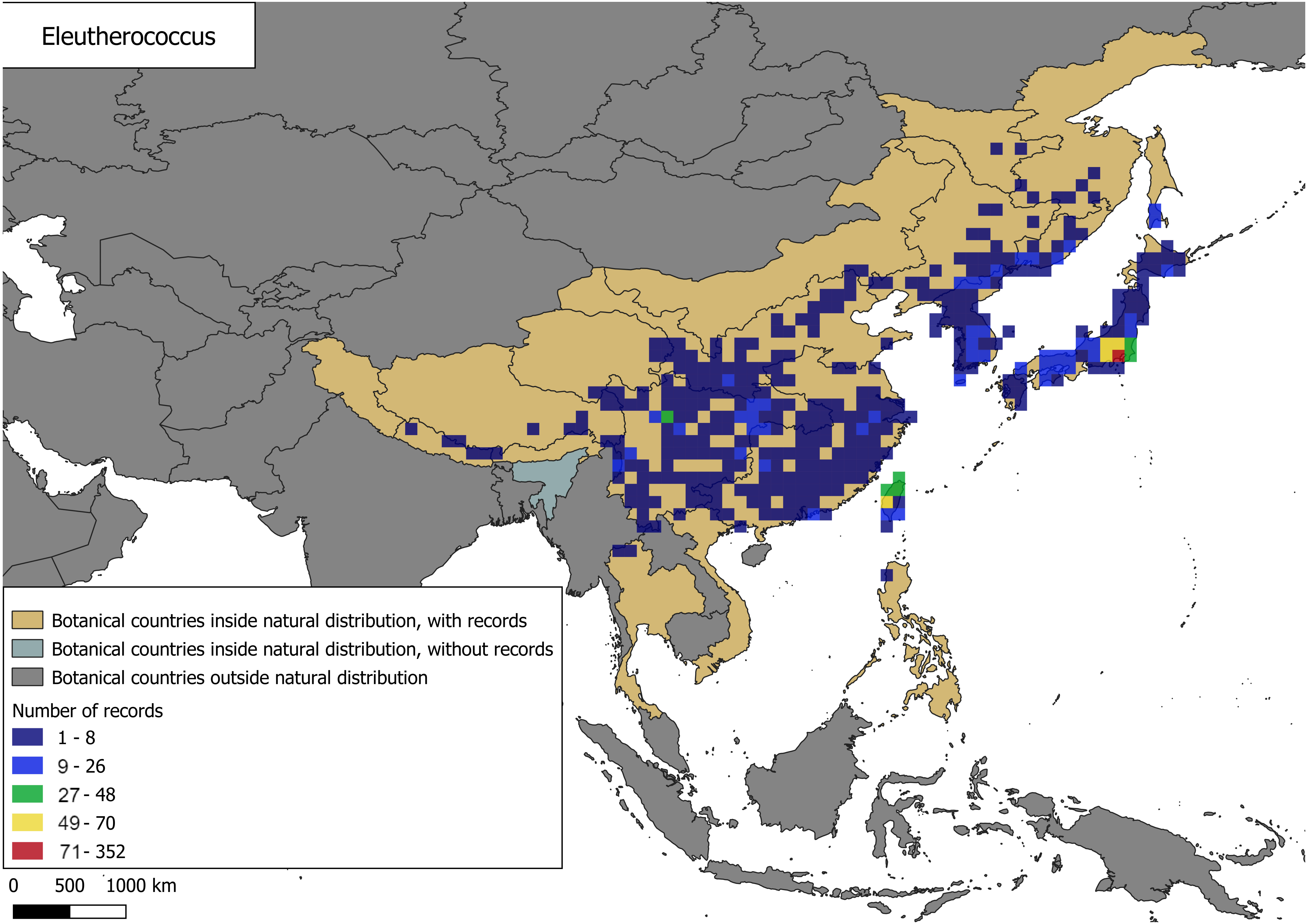
Eleutherococcus
Botanical countries inside natural distribution, with records
Botanical countries inside natural distribution, without records
Botanical countries outside natural distribution
Number of records
1 - 8
9 - 26
27 - 48
49 - 70
71 - 352
0 500 1000 km

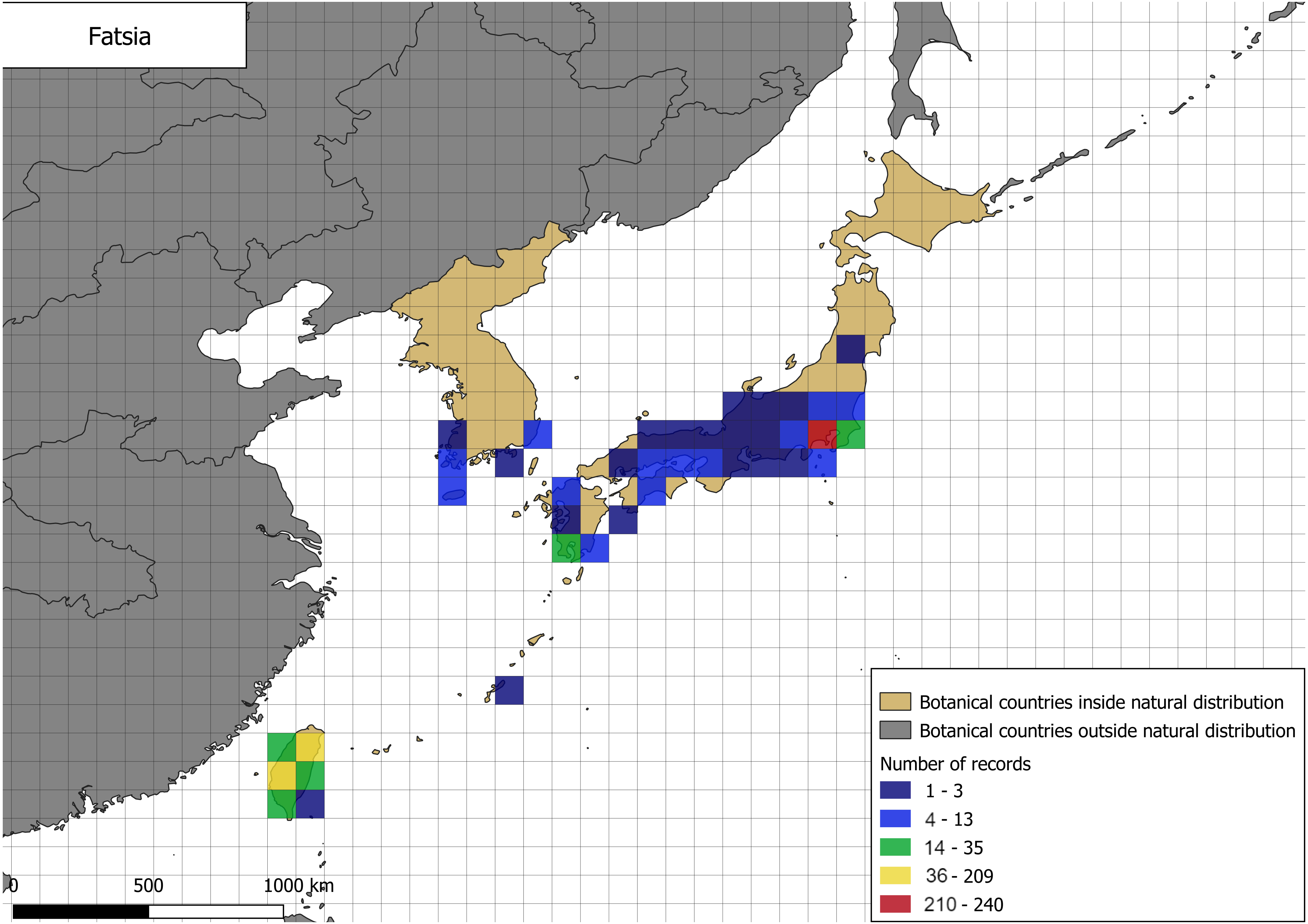
Fatsia
Botanical countries inside natural distribution
Botanical countries outside natural distribution
Number of records
1 - 3
4 - 13
14 - 35
36 - 209
210 - 240
0
500
1000 km

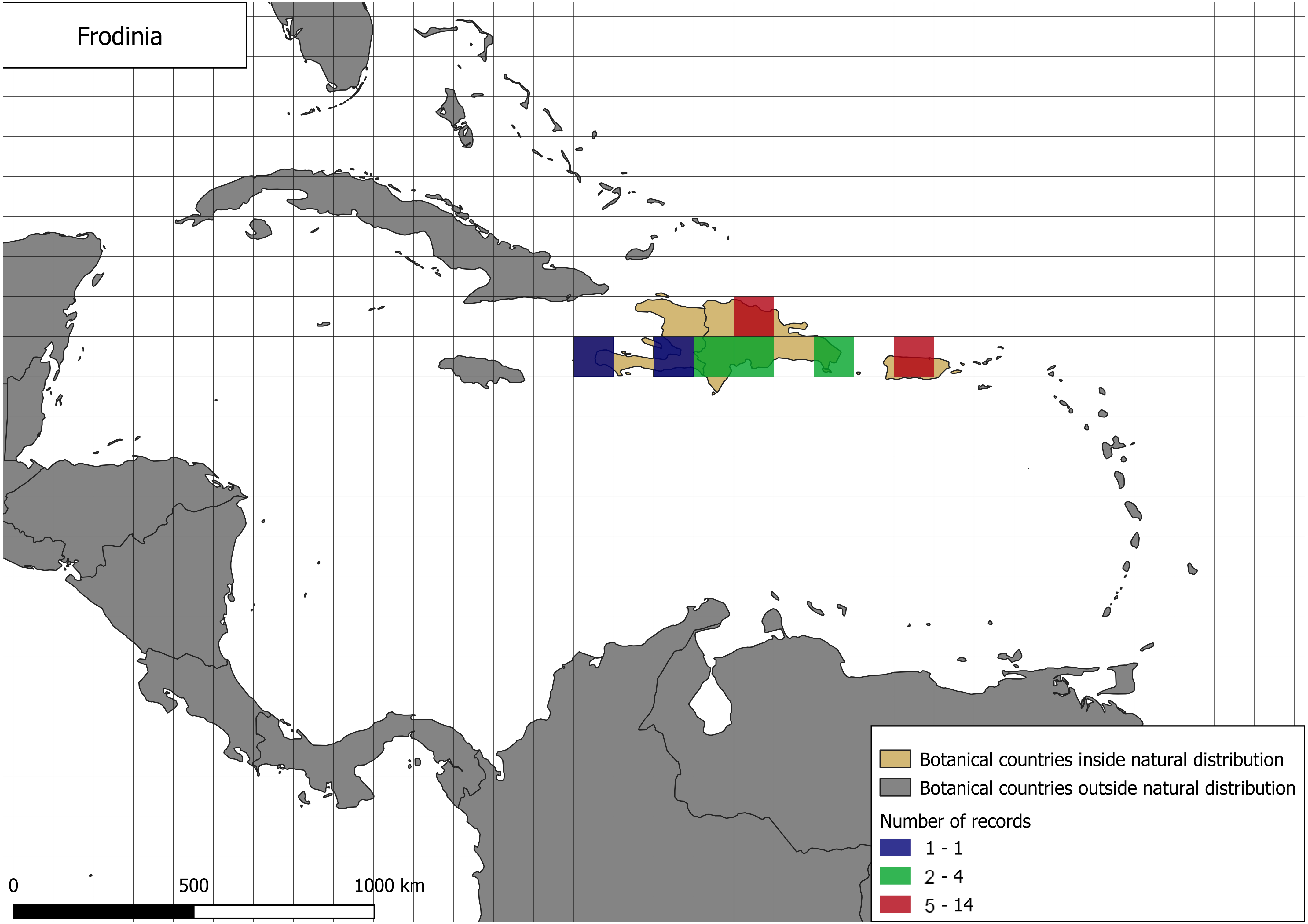
Frodinia
Botanical countries inside natural distribution
Botanical countries outside natural distribution
Number of records
1 - 1
2 - 4
5 - 14
0
500
1000 km

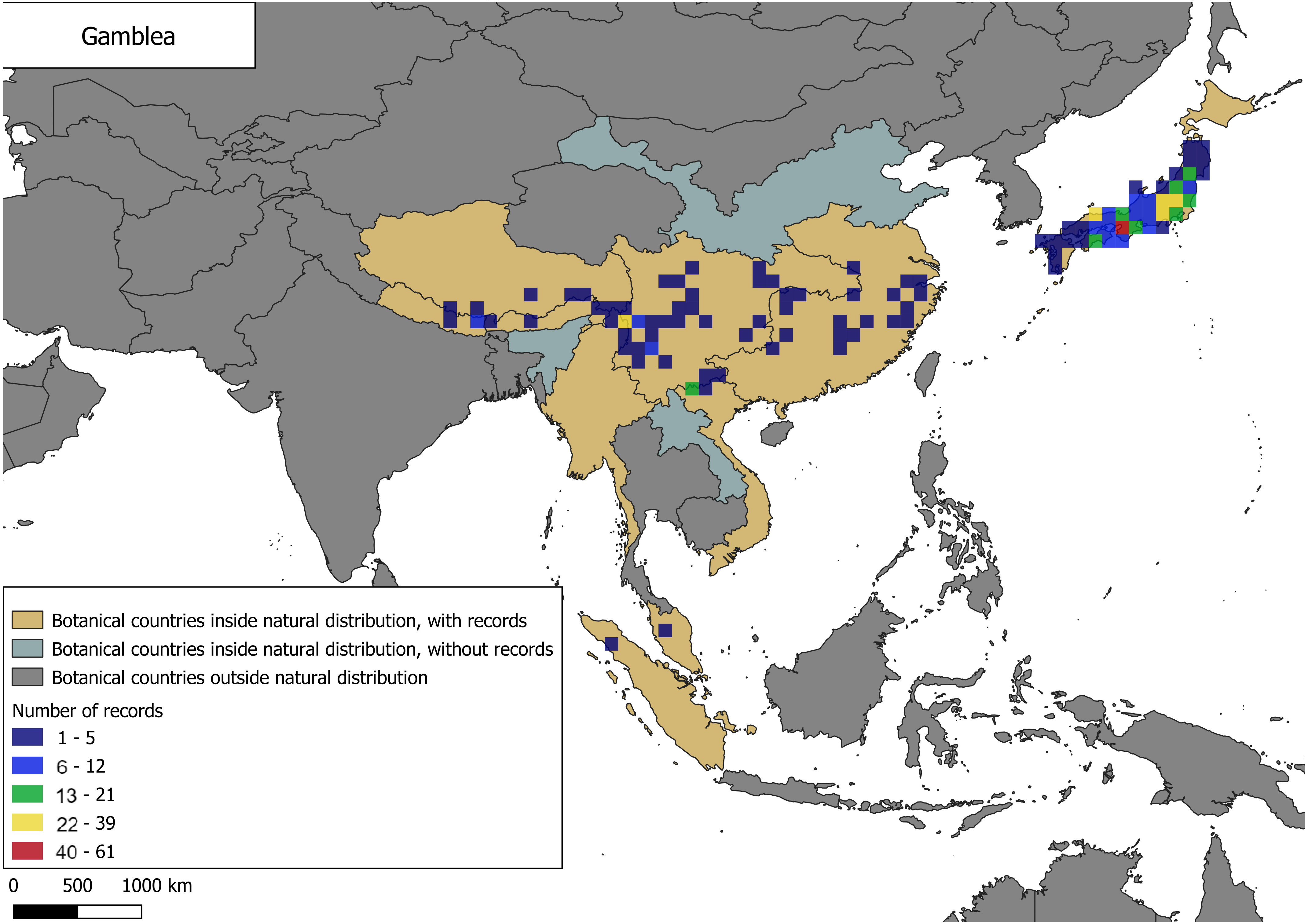
Gamblea
Botanical countries inside natural distribution, with records
Botanical countries inside natural distribution, without records
Botanical countries outside natural distribution
Number of records
1 - 5
6 - 12
13 - 21
22 - 39
40 - 61
0
500
1000 km

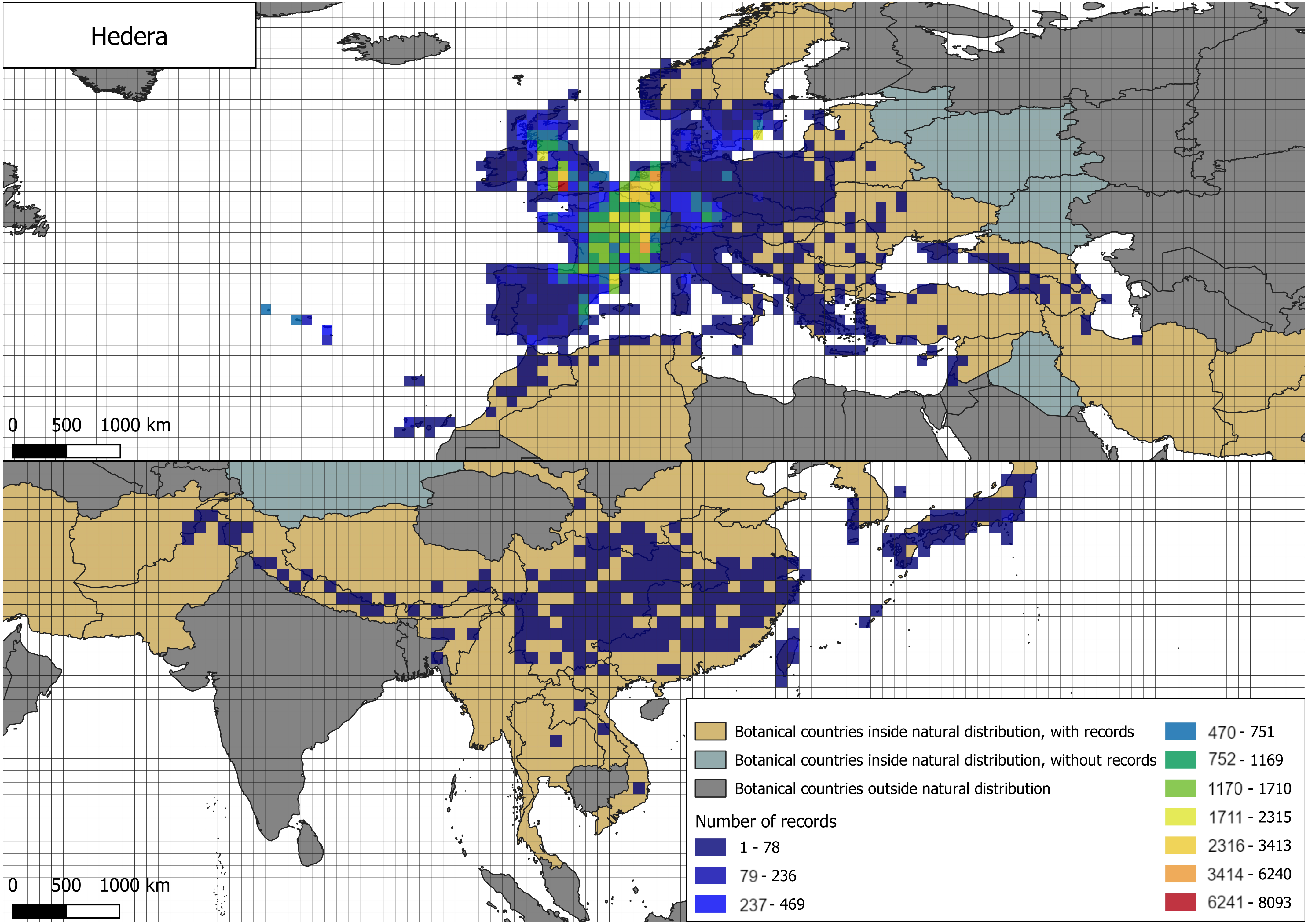
Hedera
0 500 1000 km
0 500 1000 km
Botanical countries inside natural distribution, with records
Botanical countries inside natural distribution, without records
Botanical countries outside natural distribution
Number of records
1 - 78
79 - 236
237 - 469
470 - 751
752 - 1169
1170 - 1710
1711 - 2315
2316 - 3413
3414 - 6240
6241 - 8093

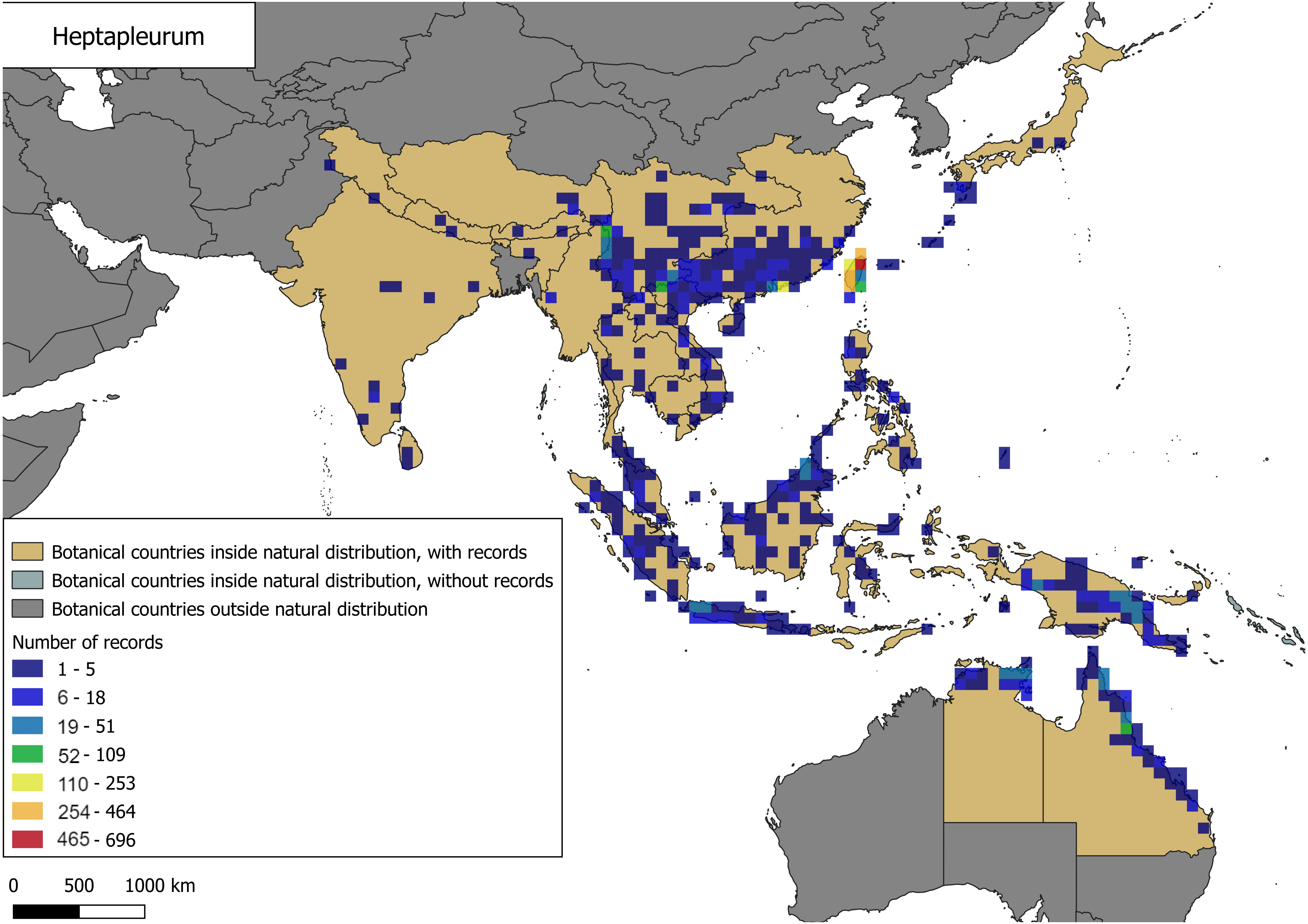
Heptapleurum
Botanical countries inside natural distribution, with records
Botanical countries inside natural distribution, without records
Botanical countries outside natural distribution
Number of records
1 - 5
6 - 18
19 - 51
52 - 109
110 - 253
254 - 464
465 - 696
0
500
1000 km

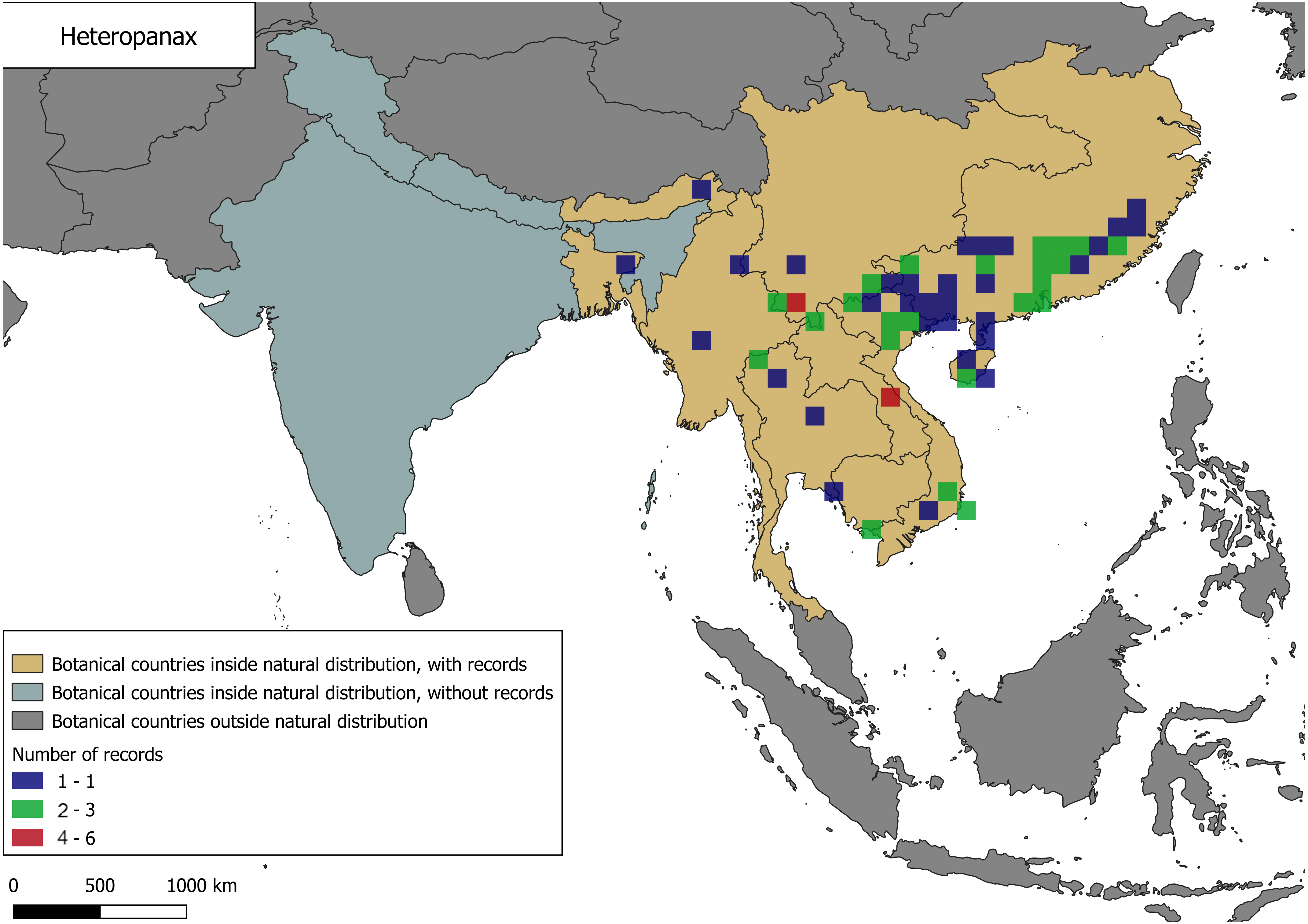
Heteropanax
Botanical countries inside natural distribution, with records
Botanical countries inside natural distribution, without records
Botanical countries outside natural distribution
Number of records
1 - 1
2 - 3
4 - 6
0
500
1000 km

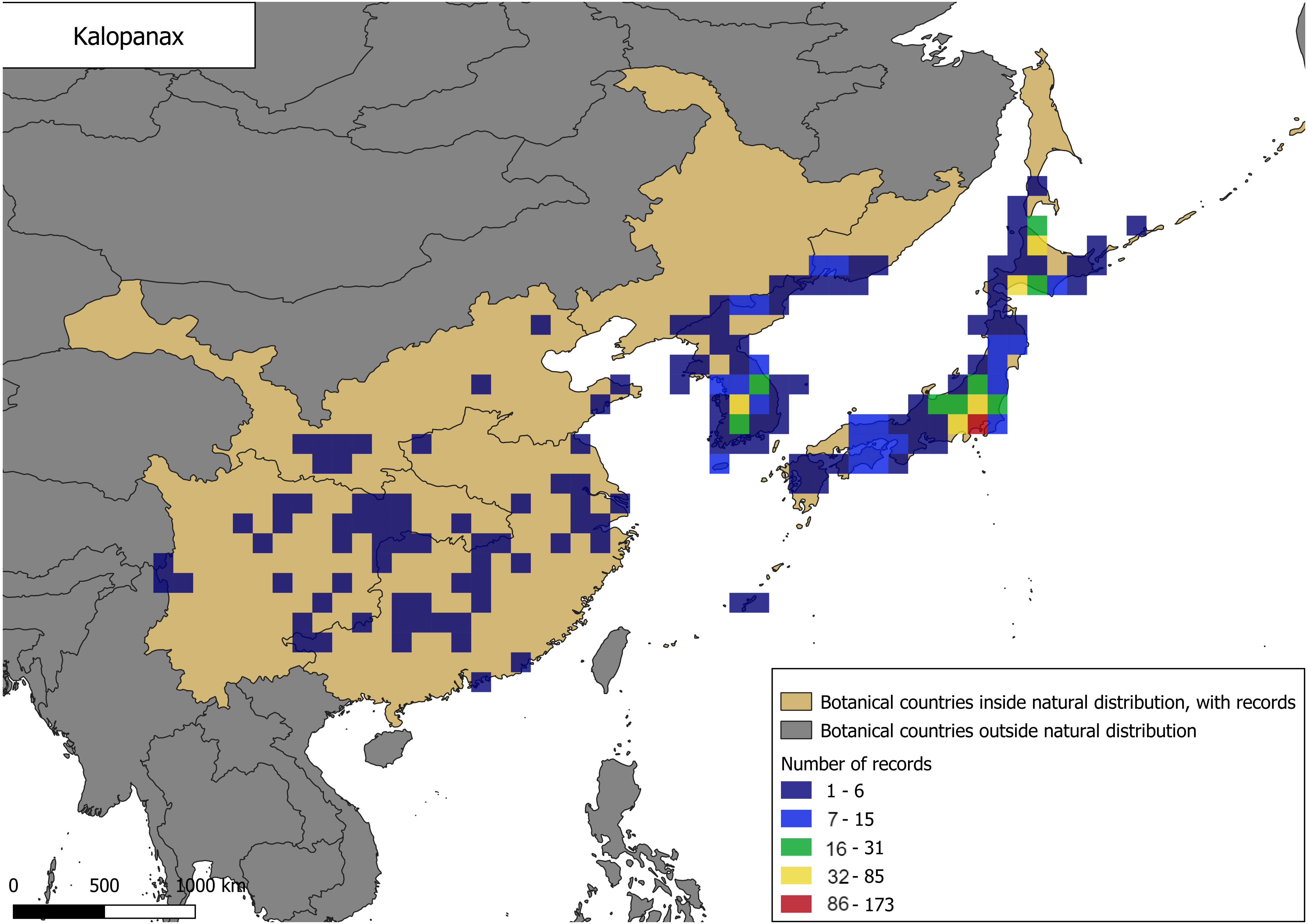
Kalopanax
Botanical countries inside natural distribution, with records
Botanical countries outside natural distribution
Number of records
1 - 6
7 - 15
16 - 31
32 - 85
86 - 173
0
500
1000 km

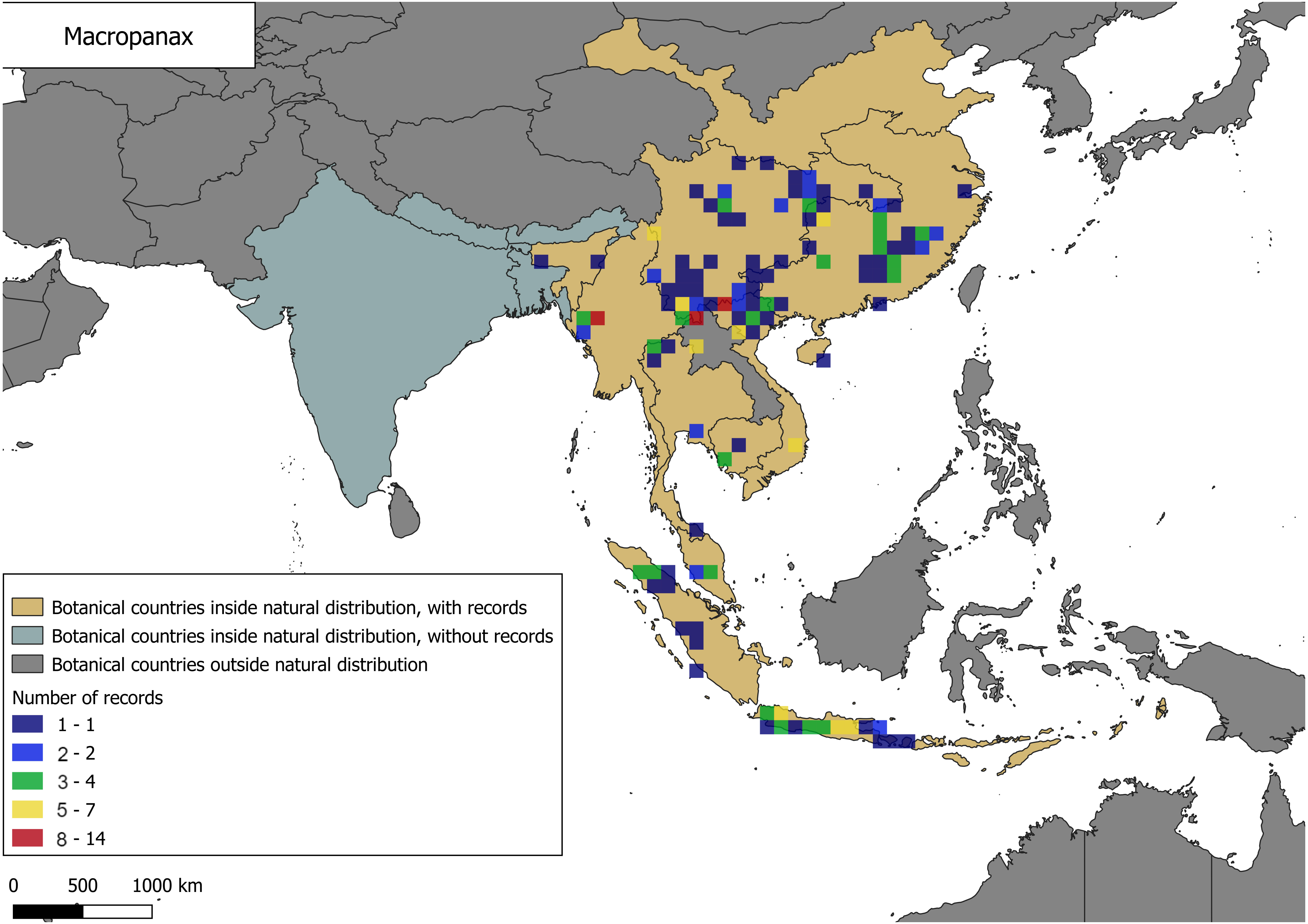
Macropanax
Botanical countries inside natural distribution, with records
Botanical countries inside natural distribution, without records
Botanical countries outside natural distribution
Number of records
1 - 1
2 - 2
3 - 4
5 - 7
8 - 14
0
500
1000 km

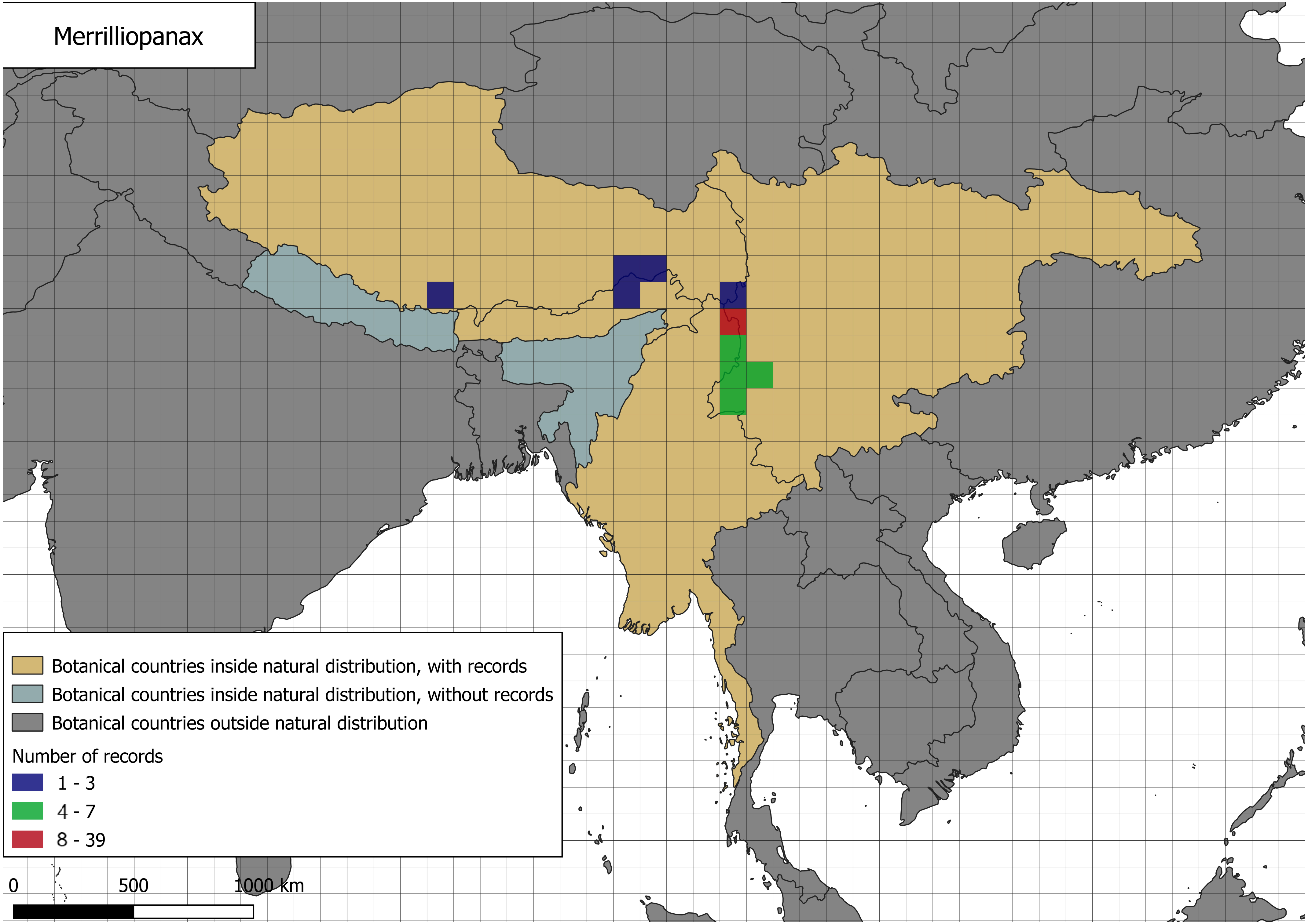
Merrilliopanax
Botanical countries inside natural distribution, with records
Botanical countries inside natural distribution, without records
Botanical countries outside natural distribution
Number of records
1 - 3
4 - 7
8 - 39
0
500
1000 km

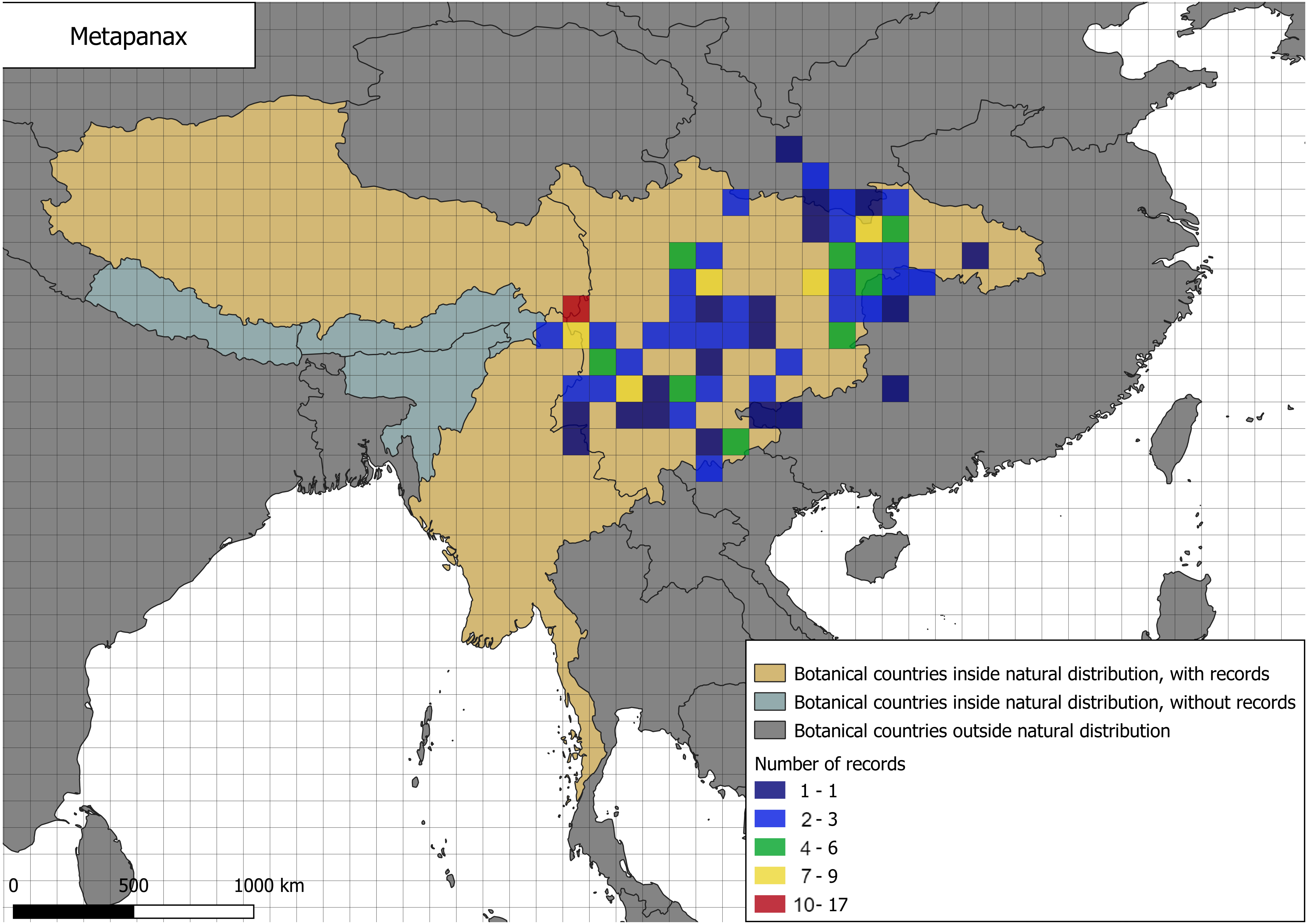
Metapanax
Botanical countries inside natural distribution, with records
Botanical countries inside natural distribution, without records
Botanical countries outside natural distribution
Number of records
1 - 1
2 - 3
4 - 6
7 - 9
10- 17
0
500
1000 km

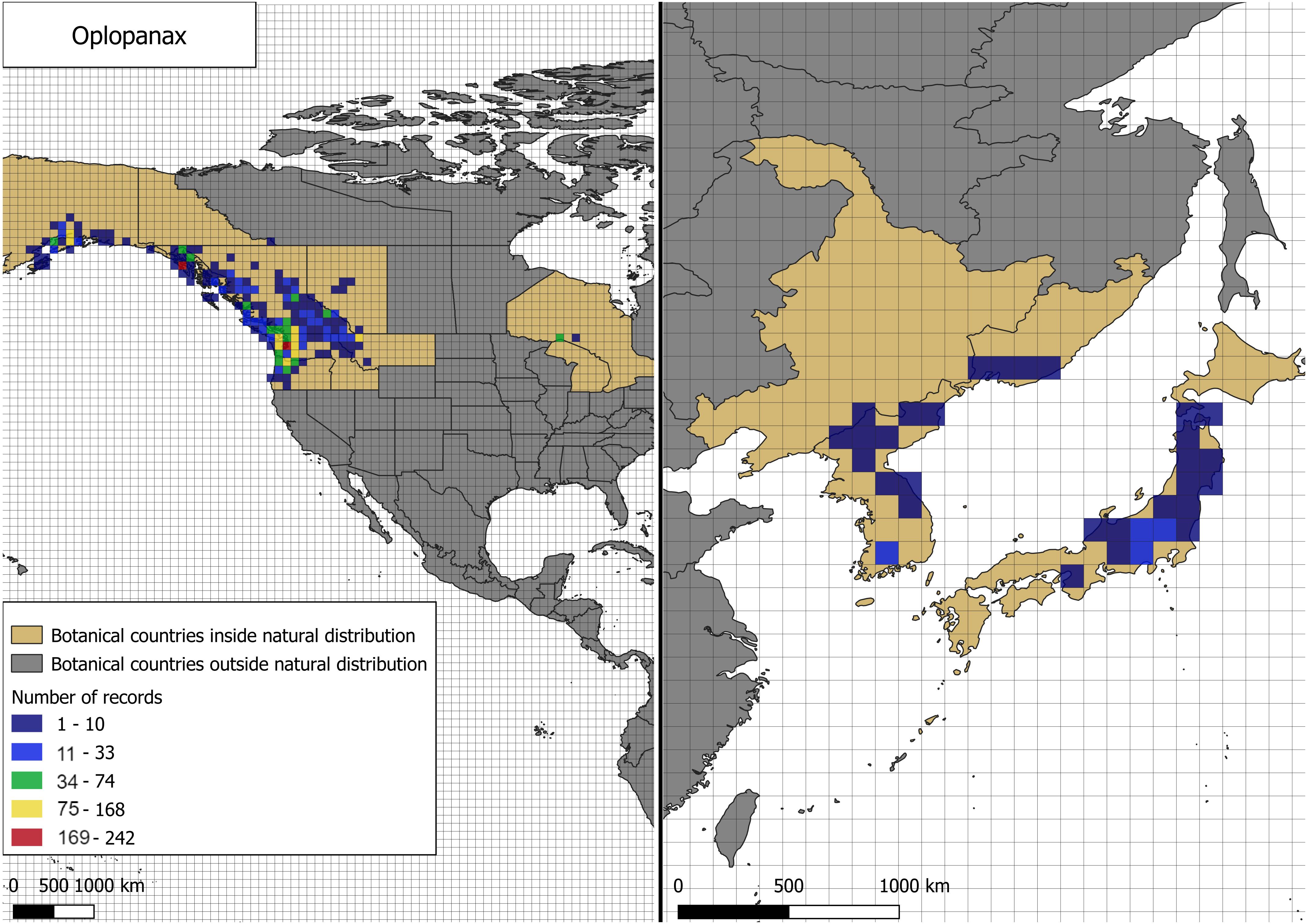

Oplopanax
Botanical countries inside natural distribution
Botanical countries outside natural distribution
Number of records
1 - 10
11 - 33
34 - 74
75 - 168
169 - 242
0 500 1000 km
0 500 1000 km

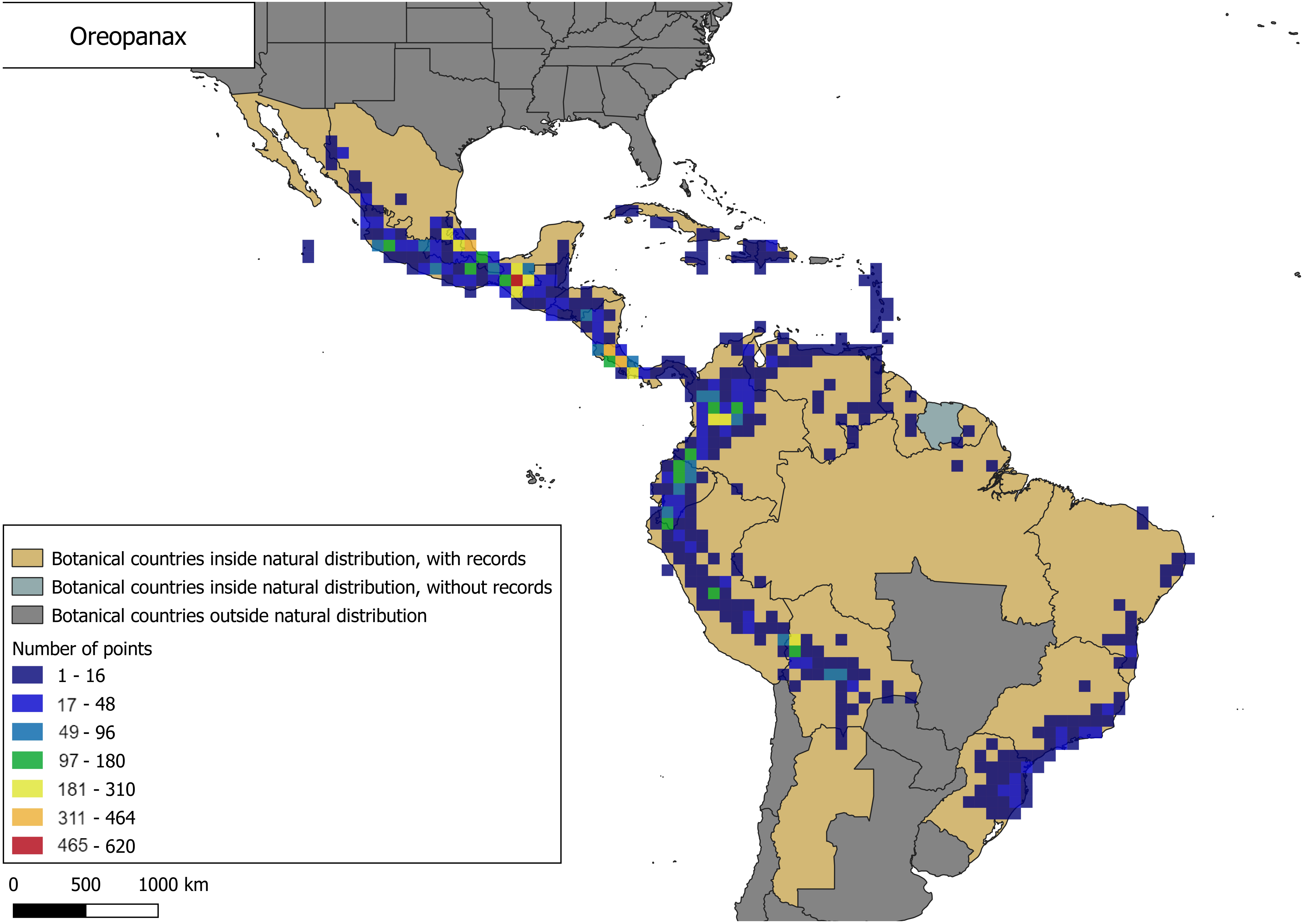
Oreopanax
Botanical countries inside natural distribution, with records
Botanical countries inside natural distribution, without records
Botanical countries outside natural distribution
Number of points
1 - 16
17 - 48
49 - 96
97 - 180
181 - 310
311 - 464
465 - 620
0
500
1000 km

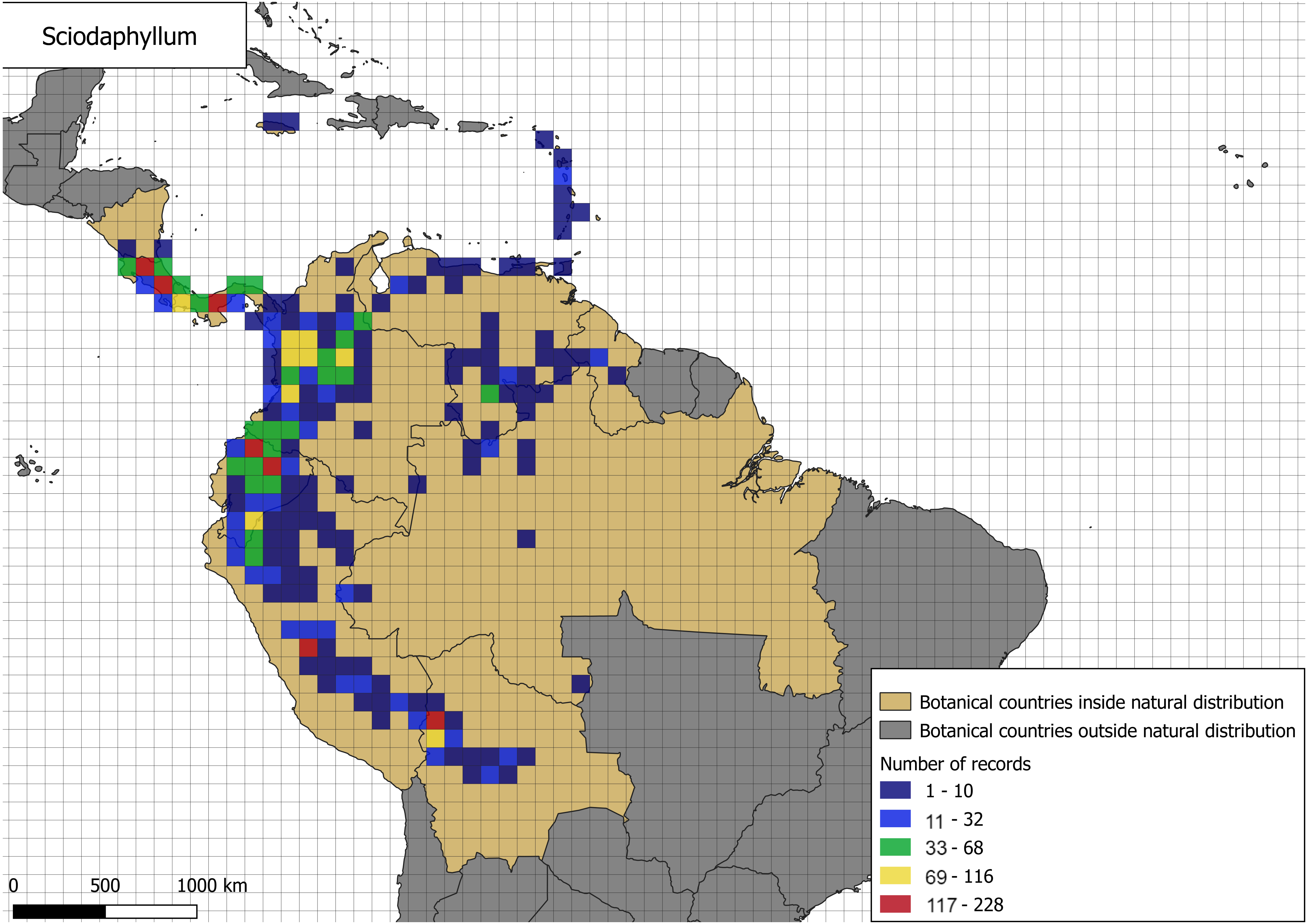
Sciodaphyllum
Botanical countries inside natural distribution
Botanical countries outside natural distribution
Number of records
1 - 10
11 - 32
33 - 68
69 - 116
117 - 228
0
500
1000 km

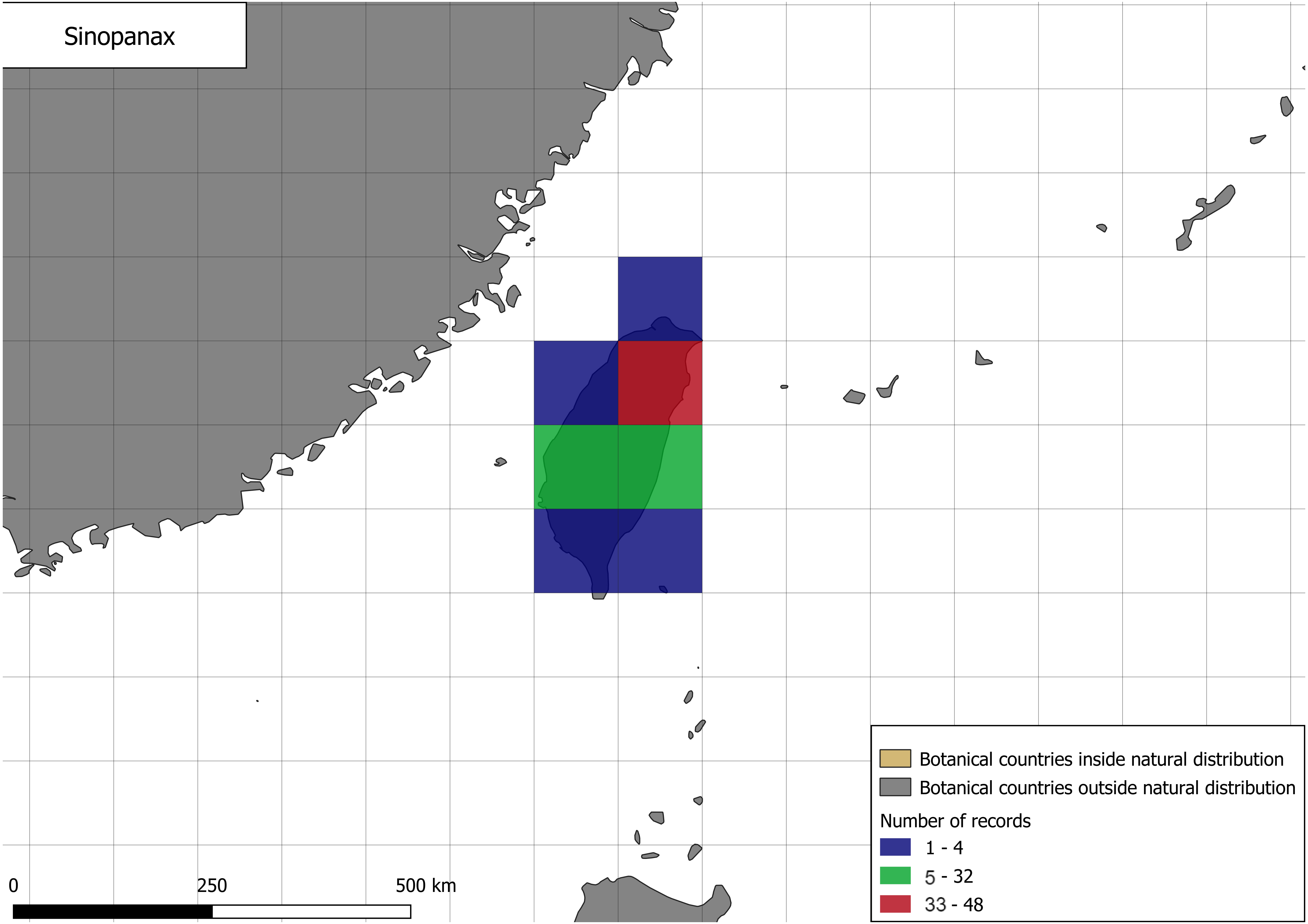
Sinopanax
0
250
500 km
Botanical countries inside natural distribution
Botanical countries outside natural distribution
Number of records
1 - 4
5 - 32
33 - 48

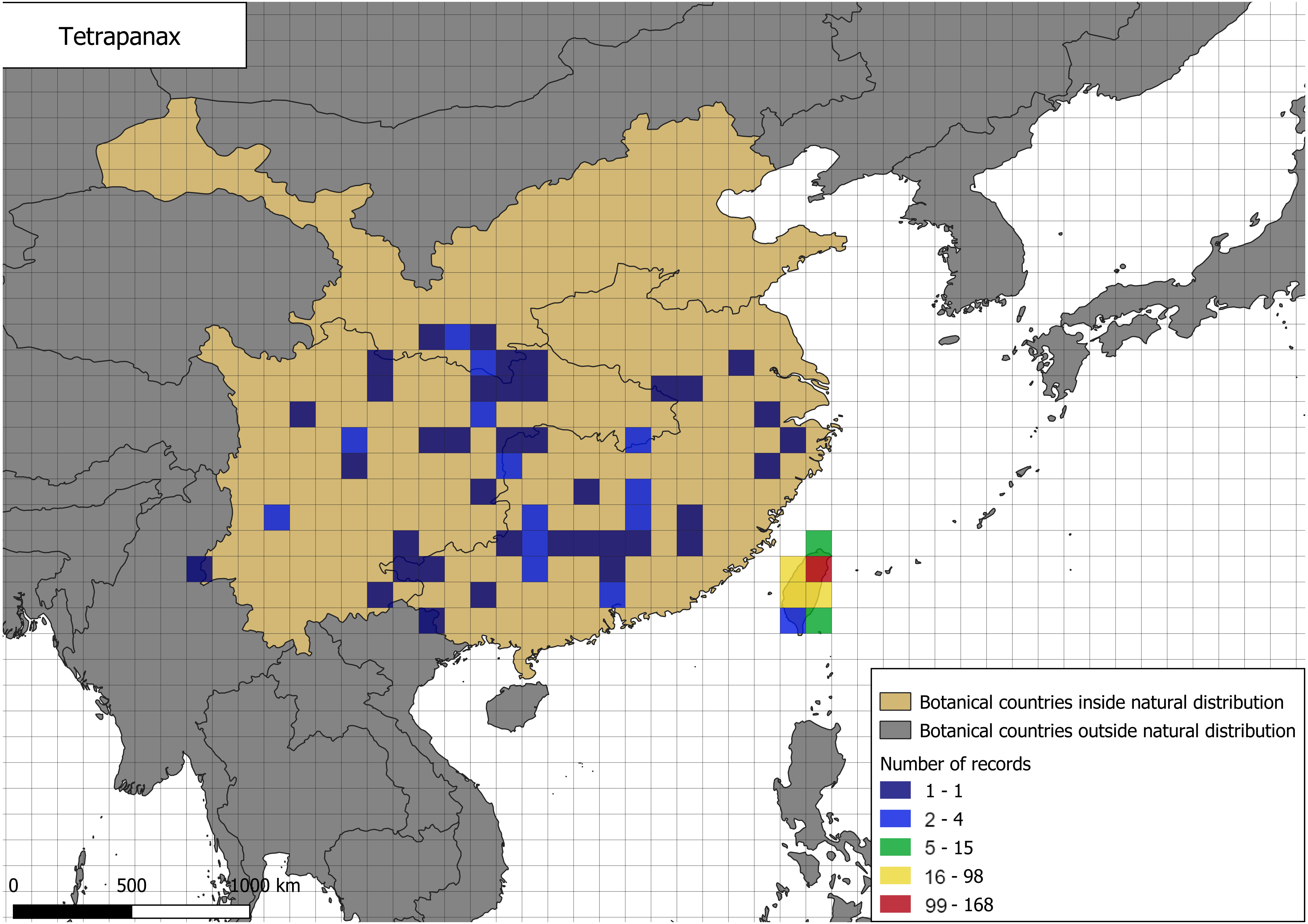
Tetrapanax
Botanical countries inside natural distribution
Botanical countries outside natural distribution
Number of records
1 - 1
2 - 4
5 - 15
16 - 98
99 - 168
0
500
1000 km

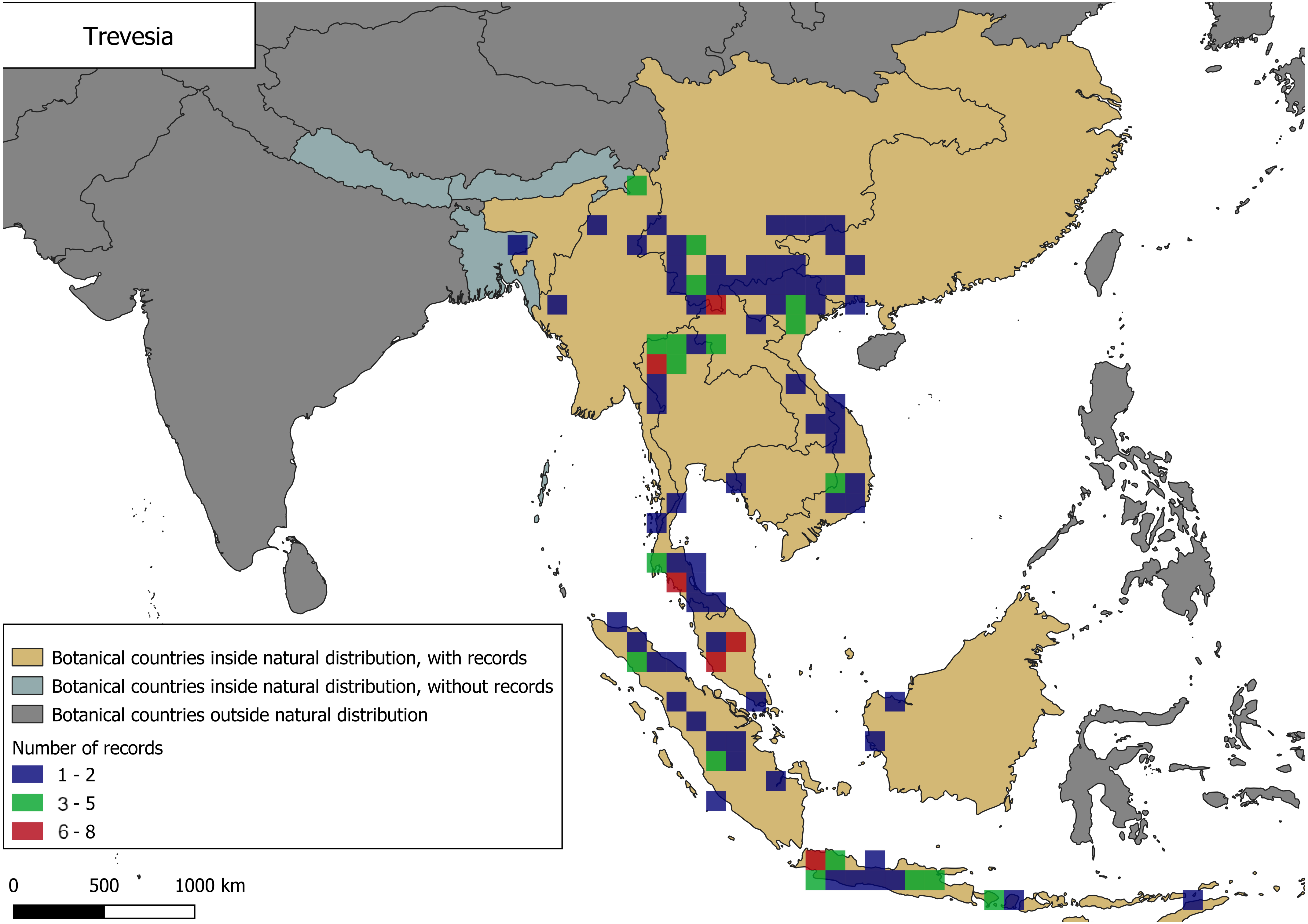
Trevesia
Botanical countries inside natural distribution, with records
Botanical countries inside natural distribution, without records
Botanical countries outside natural distribution
Number of records
1 - 2
3 - 5
6 - 8
0
500
1000 km
